# Supplementary material for: Conductive Mediators in Oxidation Based on Ferrocene Functionalized Phosphonium Ionic Liquids
Source: Int J Mol Sci. 2022 Dec 8;23(24):15534. doi: 10.3390/ijms232415534 (PMC9779220; doi:10.3390/ijms232415534)
Supplement: Supplementary file 1 [file ijms-23-15534-s001.zip › ijms-2059035-supplementary.pdf]

Supplementary Information for:

Conductive mediators in oxidation based on ferrocene functionalized phosphonium ionic liquids

Vadim V. Ermolaev [1], Liliya R. Kadyrgulova [1], Mikhail N. Khrizanforov [1,2,*], Tatiana P. Gerasimova [1], Gulnaz R. Baembitova [1,2], Anna A. Lazareva [1,2] and Vasili A. Miluykov [1]

[1] Arbuzov Institute of Organic and Physical Chemistry, FRC Kazan Scientific Center, Russian Academy of Sciences, 8 Arbuzov Street, 420088 Kazan, Russian Federation

[2] Kazan Federal University, Kremlevskaya Str. 18, 420008 Kazan, Russian Federation

* Correspondence: khrizanforov@iopc.ru or khrizanforov@gmail.com

Experimental section

Materials and Methods

NMR experiments

NMR spectra were recorded with multi-nuclear spectrometer Bruker AVANCE-400 (400.1 MHz (^{1}H), 100.6 MHz (^{13}C) and 162.0 MHz (^{31}P)). Chemical shifts are given in parts per million relative to $SiMe_4$ (1H, internal solvent) and 85% H_3PO_4 (^{31}P, external).

Electrochemical measurements

Electrochemical measurements were taken on a BASi Epsilon EClipse electrochemical analyzer (West Lafayette, IN, USA). The program concerned Epsilon-ECUSB-V200 waves at potential scan rate t = 100 mV·s^{-1} in CH_3CN / without an electrolyte (Except clear ascorbic acid – for ascorbic acid, a 0.1M solution of Bu_4NBF_4 was used.) at 295 K. A glassy carbon working electrode (ð = 2 mm) embedded in Teflon and Pt wire as counter electrode were used in electrochemical cell. Before each measurement the surface of the working electrode was mechanically polished.

TG-DSC

Thermogravimetric analysis was performed on the NETZSCH STA 449F1 with a heating rate 10 K per minute up to 400°C in an argon atmosphere.

Mass-spectra

Mass Electrospray ionization mass spectrometry (ESI-MS) was performedon the AmazonX mass spectrometer (Bruker Daltonik GmbH, Bremen, Germany). The measurements were carried out in the positive/negative ion detection mode in the m/z range from 100 to 1000. The voltage on the capillary was 140 V. Data was processed using the DataAnalysis 4.0 program (Bruker Daltonik GmbH, Bremen, Germany)

IR-spectra

IR-spectra were recorded in KBr o ras emulsions in vaseline oil (sample concentration 0.25 %) on a Bruker Tensor-27 spectrometer in the range 400-4000 cm^{-1}.

Reagents and research subjects

All the work related to the preparation of the starting substrates, the synthesis and the workup of products, was carried out in an inert atmosphere using standard Schlenk apparatus. All solvents and purchased reagents were absolute by the appropriate methods, mainly by distillation in an inert atmosphere.

General procedure for the synthesis of 3-bromopropanoyl chloride 1a-1d. To a 50 ml Schlenk vessel with stirrer, halogenated acid (1 eq.) and $SOCl_2$ (4.2 eq.) were added at room temperature and gradually warmed to reflux. The apparatus should be connected to a bubbler and an exhaust gas absorbing device. The reaction mixture was reflux within 4 h and then was stirred within 12 h at room temperature. The crude product was purified by vacuum distillation.

General procedure for the synthesis of 2a-2d.

Under an inert gas atmosphere, the ω-bromoalkanoyl chloride (1 eq.) and anhydrous $AlCl_3$ (1eq.) were added to dry CH_2Cl_2 (50 ml) at 0 °C. The mixture was stirred for ten minutes before addition of ferrocene (1.2 eq.) at the same temperature. The appearance of a purple colour signalizes the beginning of the acylation process. Stirring was continued after addition overnight while the mixture was allowed to warm up to room temperature. The mixture was again cooled to 0 °C by an ice bath and water

(40 ml) was carefully added under an inert gas atmosphere to quench the reaction. After quenching more H_2O (50 ml) was added and the mixture was stirred for 30 minutes at 0 °C. The aqueous phase was extracted four times with CH_2Cl_2 (4 x 60 ml). The combined organic extracts were washed sequentially with sat. $NaHCO_3$ (2 x 100 ml), H_2O (100 ml) and brine (100 ml). The organic phase was dried over $MgSO_4$, concentrated and the residue purified by column chromatography (SiO_2; petroleum ether/ethyl acetate). All products were dried in vacuo after purification.

1-(3-bromopropionyl) ferrocene (2a). Yield 2.043 g (64%). ^1H NMR ($CDCl_3$, δ, ppm) 3.33 (t, $^3J_{HH}$ = 6.58 Hz, 2H, C(O)CH_2), 3.76 (t, $^3J_{HH}$ = 6.57 Hz, 2H, CH_2Br), 4.29 (s, 5H, H_{Cp}), 4.57 (m, 2H, H_{Cp}), 4.83 (m, 2H, H_{Cp}).

1-(5-bromopentanoyl) ferrocene (2b). Yield 3.429 g (57%). ^1H NMR ($CDCl_3$, δ, ppm) 1.92 (m, 2H, CH_2), 1.99 (m, 2H, CH_2), 2.77 (m, 2H, C(O)CH_2), 3.49 (t, $^3J_{HH}$ = 6.59 Hz, 2H, CH_2Br), 4.25 (s, 5H, H_{Cp}), 4.56 (m, 2H, H_{Cp}), 4.84 (m, 2H, H_{Cp}).

1-(6-bromohexanoyl) ferrocene (2c). Yield 3.071 g (69%). ^1H NMR ($CDCl_3$, δ, ppm) 1.56 (m, 2H, CH_2), 1.78 (m, 2H, CH_2), 1.95 (m, 2H, CH_2), 2,75 (m, 2H, C(O)CH_2), 3.47 (t, $^3J_{HH}$ = 6.67 Hz, 2H, CH_2Br), 4.24 (s, 5H, H_{Cp}), 4.54 (m, 2H, H_{Cp}), 4.82 (m, 2H, H_{Cp}).

1-(11-bromoundecanoyl) ferrocene (2d). Yield 3.853 г (85%). ^1H NMR ($CDCl_3$, δ, ppm) 1.34 (m, 10H, CH_2), 1.45 (m, 2H, CH_2), 1.73 (m, 2H, CH_2), 1.88 (m, 2H, CH_2), 2,72 (t, $^3J_{HH}$ = 7.29 Hz, 2H, C(O)CH_2), 3.43 (t, $^3J_{HH}$ = 6.74 Hz, 2H, CH_2Br), 4.22 (s, 5H, H_{Cp}), 4.51 (m, 2H, H_{Cp}), 4.80 (m, 2H, H_{Cp}).

General procedure for the synthesis of 3a-3d. Bromoalkanoyl ferrocene was dissolved in dry MeCN (20 ml) and an equivalent amount of tri-tert-butylphosphine was added. The reaction mixture was stirred at 82 °C for 6 hours. The solvent was removed in vacuo and the product was stirred with 3 portions of 20 ml of petroleum ether and 3 portions of 20 ml of diethyl ether. After each stirring, the solvent was removed with a cannula with a filter nozzle, the solvent residues are removed in vacuo.

Tri-*tert*-butyl(3-ferrocenyl-3-oxopropyl) phosphonium bromide (3a). Light brown viscous oil. Temperature of the decomposition 178°C. Yield 1.472 g (81%). ^1H NMR ($CDCl_3$, δ, ppm) 1.67 (d, $^3J_{HP}$ = 13.98 Hz, 27H, $P(C(CH_3)_3)_3$), 3.24 (m, 2H, P-CH_2), 3.59 (m, 2H, C(O)CH_2), 4.34 (s, 5H, H_{Cp}), 4.58 (s, 2H, H_{Cp}), 4.97 (s, 2H, H_{Cp}). ^{31}P NMR ($CDCl_3$, δ, ppm) 52.00 (s). ^{13}C NMR ($CDCl_3$, δ, ppm) 11.62 (d, $^1J_{PC}$ = 39.91 Hz, P-CH_2),

30.24 (s, C($\underline{C}H_3)_3$), 34.21 (s, P-CH_2-$\underline{C}H_2$), 39.40 (d, $^{1}J_{PC}$ = 38.42 Hz, P-($\underline{C}(CH_3))_3$), , 70.12 (s, C_p), 70.59 (s, C_p), 72.89 (s, C_p), 77.55 (s, C_p) 201.01 (s, C(O)).

Tri-*tert*-butyl(5-ferrocenyl-5-oxopentyl) phosphonium bromide (3b). Brown amorphous tar. Melting point 152 °C. Yield 1.359 g (78%). ^{1}H NMR ($CDCl_3$, δ, ppm) 1.69 (d, $^{3}J_{HP}$ = 13.99 Hz, 27H, $P(C(CH_3)_3)_3$), 2.15 (m, 4H, CH_2), 2.76 (m, 2H, CH_2), 2.95 (m, 2H, C(O)CH_2), 4.21 (s, 5H, H_{Cp}), 4.53 (s, 2H, H_{Cp}), 4.88 (s, 2H, H_{Cp}). ^{31}P NMR ($CDCl_3$, δ) 51.28 (s). ^{13}C NMR ($CDCl_3$, δ, ppm) 18.25 (d, P-CH_2), 24.55 (s,CH_2) 26.42 (m, P-CH_2-CH_2-$\underline{C}H_2$), 26.80 (s, C($\underline{C}H_3$)), 32.51 (d, P-CH_2-$\underline{C}H_2$), 39.04 (s, C(O)$\underline{C}H_2$), 40.39 (d, $^{1}J_{PC}$= 28.91 Hz, P-($\underline{C}(CH_3)_3$), 70.38 (s, C_p), 71.24 (s, C_p), 72.78 (s, C_p), 73.27 (s, C_p), 195.61 (s, C(O)).

Tpri-*tert*-butyl(6-ferrocenyl-6-oxohexyl) phosphonium bromide (3c). Light brown viscous oil. Temperature of the decomposition 159 °C. Yield 1.273 g (72%). ^{1}H NMR ($CDCl_3$, δ, ppm) 1.70 (d, $^{3}J_{HP}$ = 13.95 Hz, 27H, $P(C(CH_3)_3)_3$), 1.84 (m, 2H, CH_2), 1.92 (m, 2H, CH_2), 2.03 (m, 2H, CH_2), 2.85 (m, 4H, CH_2), 4.22 (s, 5H, H_{Cp}), 4.51 (s, 2H, H_{Cp}), 4.83 (s, 2H, H_{Cp}). ^{31}P NMR ($CDCl_3$, δ, ppm) 50.22 (s). ^{13}C NMR ($CDCl_3$, δ, ppm) 21.64 (d, $^{1}J_{PC}$ = 32.81 Hz, P-CH_2), 24.66 (s, CH_2), 25.99 (d, $^{3}J_{PC}$ = 5.24 Hz, P-CH_2- CH_2-$\underline{C}H_2$), 31.78 (s, C($\underline{C}H_3)_3$), 32.58 (d, $^{2}J_{PC}$ = 12.60 Hz, P-CH_2-$\underline{C}H_2$), 39.77 (d, $^{1}J_{PC}$ = 29.38 Hz, P-($\underline{C}(CH_3))_3$), 40.67 (s, C(O)$\underline{C}H_2$), 70.25 (s, C_p), 70.51 (s, C_p), 72.59 (s, C_p), 79.06 (s, C_p), 204.81 (s, C(O)).

Tri-*tert*-butyl(11-ferrocenyl-11-oxoundecyl) phosphonium bromide (3d). Dark orange viscous oil. Temperature of the decomposition 172 °C. Yield 1.394 g (82%). ^{1}H NMR ($CDCl_3$, δ, ppm) 1.35 (m, 12H, CH_2), 1.70 (d, $^{3}J_{HP}$ = 13.94 Hz, 27H, $P(C(CH_3)_3)_3$), 1.76 (s, 2H, CH_2), 1.94 (m, 2H, P-CH_2-$C\underline{H}_2$), 2.66 (m, 2H, P-$C\underline{H}_2$), 2.71 (t, $^{3}J_{HH}$ = 7.47 Hz, 2H, C(O)CH_2), 4.21 (s, 5H, H_{Cp}), 4.51 (s, 2H, H_{Cp}), 4.80 (s, 2H, H_{Cp}). ^{31}P NMR ($CDCl_3$, δ, ppm) 49.60 (s). ^{13}C NMR ($CDCl_3$, δ, ppm) 19.49 (d, $^{1}J_{PC}$ = 34.67 Hz, P-$\underline{C}H_2$), 24.49 (s, CH_2), 25.17 (d, $^{3}J_{PC}$ = 5.14 Hz, P-CH_2-CH_2-$\underline{C}H_2$), 29.28 (s, CH_2), 29.30 (s, CH_2), 29.32 (s, CH_2), 29.33 (s, CH_2), 30.43 (s, C($\underline{C}H_3)_3$), 32.12 (d, $^{2}J_{PC}$ = 11.46 Hz, P-CH_2-$\underline{C}H_2$), 39.31 (d, $^{1}J_{PC}$ = 29.28 Hz, P-($\underline{C}(CH_3))_3$), 39.67 (s, C(O)$\underline{C}H_2$), 69.28 (s, C_p), 69.72 (s, C_p), 72.18 (s, C_p), 79.07 (s, C_p), 204.56 (s, C(O)).

General procedure for the synthesis of 4a-4d. Salts **3a-d** were dissolved in 10 ml of water, a twofold excess of sodium tetrafluoroborate solution in water was added to them. The reaction mixture was stirred for 12 hours. The precipitate was filtered, the solvent residues are removed in vacuo. The salt was dissolved in 20 ml of methylene

chloride and washed with distilled water. The organic phase was separated and dried over magnesium sulfate. The solvent is evaporated, and the salt is dried under vacuum at 40 °C for 8 hours.

Tri-*tert*-butyl(3-ferrocenyl-3-oxopropyl) phosphonium tetrafluoroborate (4a). Orange amorphous mass. Melting point 167 °C. Yield 0.627 g (88%). ^{1}H NMR ($CDCl_3$, δ, ppm) 1.70 (d, $^{3}J_{HP}$ = 13.99 Hz, 27H, $P(C(CH_3)_3)_3$), 2.92 (m, 2H, P-C$\underline{H}_2$), 3.50 (m, 2H, $C(O)CH_2$), 4.32 (s, 5H, H_{Cp}), 4.62 (s, 2H, H_{Cp}), 4.98 (s, 2H, H_{Cp}). ^{31}P NMR ($CDCl_3$, δ, ppm) 51.43 (s). ^{13}C NMR ($CDCl_3$, δ, ppm) 11.26 (d, $^{1}J_{PC}$ = 41.22 Hz, P-($\underline{C}(CH_3))_3$), 30.36 (s, C($\underline{C}H_3)_3$), 33.55 (s, C(O)$\underline{C}H_2$), 39.56 (d, $^{1}J_{PC}$ = 29.12 Hz, P-$\underline{C}H_2$), 70.36 (s, C_p), 70.84 (s, C_p), 73.54 (s, C_p), 77.35 (s, C_p), 200.22 (s, C(O)).

Tri-*tert*-butyl(5-ferrocenyl-5-oxopentyl) phosphonium tetrafluoroborate (4b). Orange viscous oil. Temperature of the decomposition 193 °C. Yield 0.782 g (81%). ^{1}H NMR ($CDCl_3$, δ, ppm) 1.65 (d, $^{3}J_{HP}$ = 13.89 Hz, 27H, $P(C(CH_3)_3)_3$), 2.00 (m, 2H, CH_2), 2.07 (m, 2H, CH_2), 2.42 (m, 2H, CH_2), 2.94 (t, $^{3}J_{HH}$ = 6.35 Hz, 2H, $C(O)CH_2$), 4.22 (s, 5H, H_{Cp}), 4.52 (s, 2H, H_{Cp}), 4.86 (s, 2H, H_{Cp}). ^{31}P NMR ($CDCl_3$, δ, ppm) 49.54 (s). ^{13}C NMR ($CDCl_3$, δ, ppm) 18.84 (d, P-CH_2), 24.65 (s, CH_2), 24.72 (m, P-CH_2-CH_2-$\underline{C}H_2$), 26.63 (s, C($\underline{C}H_3)_3$), 30.46 (d, P-CH_2-$\underline{C}H_2$), 39.68 (d, P-($\underline{C}(CH_3))_3$), 39.98 (s, C(O)$\underline{C}H_2$), 70.01 (s, C_p), 70.32 (s, C_p), 70.47 (s, C_p), 73.00 (s, C_p), 198.10 (s, C(O)).

Tri-*tert*-butyl(6-ferrocenyl-6-oxohexyl) phosphonium tetrafluoroborate (4c). Orange viscous oil. Temperature of the decomposition 218 °C. Yield 0.721 g (87%). ^{1}H NMR ($CDCl_3$, δ, ppm) 1.66 (d, $^{3}J_{HP}$ = 13.88 Hz, 27H, $P(C(CH_3)_3)_3$), 1.77 (m, 2H, CH_2), 1.98 (m, 2H, P-CH_2-C$\underline{H}_2$), 2.41 (m, 2H, P-C$\underline{H}_2$), 2.84 (t, $^{3}J_{HH}$ = 6.91 Hz, 2H, $C(O)CH_2$), 4.23 (s, 5H, H_{Cp}), 4.52 (s, 2H, H_{Cp}), 4.85 (s, 2H, H_{Cp}). ^{31}P NMR ($CDCl_3$, δ, ppm) 49.40 (s). ^{13}C NMR ($CDCl_3$, δ, ppm) 19.01 (d, $^{1}J_{PC}$ = 34.47 Hz, P-CH_2), 23.87 (s, CH_2), 25.14 (m, P-CH_2-CH_2-$\underline{C}H_2$), 30.24 (s, C($\underline{C}H_3)_3$), 31.38 (d, $^{2}J_{PC}$ = 13.15 Hz, P-CH_2-$\underline{C}H_2$), 39.31 (d, $^{1}J_{PC}$ = 29.17 Hz, P-($\underline{C}(CH_3))_3$), 39.50 (s, C(O)$\underline{C}H_2$), 69.77 (s, C_p), 70.17 (s, C_p), 72.54 (s, C_p), 79.16 (s, C_p), 204.87 (s, C(O)).

Tri-*tert*-butyl(11-ferrocenyl-11-oxoundecyl) phosphonium tetrafluoroborate (4d). Pale orange amorphous mass. Temperature of the decomposition 215 °C. Yield. 4.428 g (92%). ^{1}H NMR ($CDCl_3$, δ, ppm) 1.35 (m, 14H, CH_2), 1.66 (d, $^{3}J_{HP}$ = 13.99 Hz, 27H, $P(C(CH_3)_3)_3$), 1.92 (m, 2H, P-CH_2-C$\underline{H}_2$), 2.35 (m, 2H, P-C$\underline{H}_2$), 2.71 (t, $^{3}J_{HH}$ = 7.20 Hz, 2H, $C(O)CH_2$), 4.21 (s, 5H, H_{Cp}), 4.51 (s, 2H, H_{Cp}), 4.80 (s, 2H, H_{Cp}). ^{31}P NMR ($CDCl_3$, δ, ppm) 49.22 (s). ^{13}C NMR ($CDCl_3$, δ, ppm) 18.55 (d, $^{1}J_{PC}$ = 35.26 Hz, P-CH_2),

24.50 (s, CH_2), 24.90 (d, $^3J_{PC}$ = 6.41 Hz, P-CH_2-CH_2-$\underline{C}H_2$), 29.02 (s, CH_2), 29.19 (s, CH_2), 29.25 (s, CH_2), 29.33 (s, CH_2), 29.79 (s, C($\underline{C}H_3$)$_3$), 31.63 (d, $^2J_{PC}$ = 12.66 Hz, P-CH_2-$\underline{C}H_2$), 39.13 (d, $^1J_{PC}$ = 29.17 Hz, P-($\underline{C}$(CH_3))$_3$), 39.59 (s, C(O)$\underline{C}H_2$), 69.26 (s, C_p), 69.70 (s, C_p), 72.21 (s, C_p), 79.08 (s, C_p), 204.61 (s, C(O)).

General procedure for the synthesis of 5a-5d. Salts **3a-d** were dissolved in 10 ml of water, a one and a half excess of lithium bis(trifluoromethanesulfonyl)imide solution in water was added to them. The reaction mixture was stirred for 1 hour. The product was extracted with CH_2Cl_2. The extract was washed with distilled water. The organic phase was separated and dried over magnesium sulfate. The solvent is evaporated, and the salt is dried under vacuum at 50 °C for 8 hours.

Tri-*tert*-butyl(3-ferrocenyl-3-oxopropyl) phosphonium bis(trifluoromethanesulfonyl)imide (5a). Yield 0.376 g (74%). ^{1}H NMR ($CDCl_3$, δ, ppm) 1.71 (d, $^3J_{HP}$ = 13.97 Hz, 27H, P(C(CH_3)$_3$)$_3$), 2.79 (m, 2H, P-C$\underline{H_2}$), 3.44 (m, 2H, C(O)CH_2), 4.27 (s, 5H, H_{Cp}), 4.65 (s, 2H, H_{Cp}), 4.97 (s, 2H, H_{Cp}). ^{31}P NMR ($CDCl_3$, δ, ppm) 51.12 (s). MS (ESI): m/z= 443.1 [M^+].

Tri-*tert*-butyl(5-ferrocenyl-5-oxopentyl) phosphonium bis(trifluoromethanesulfonyl)imide (5b). Yield 0.427 g (71%). ^{1}H NMR ($CDCl_3$, δ, ppm) 1.65 (d, $^3J_{HP}$ = 13.66 Hz, 27H, P(C(CH_3)$_3$)$_3$), 1.97 (m, 2H, CH_2), 2.07 (m, 2H, CH_2), 2.30 (m, 2H, P-CH_2), 2.92 (m, 2H, C(O)CH_2), 4.22 (s, 5H, H_{Cp}), 4.54 (s, 2H, H_{Cp}), 4.84 (s, 2H, H_{Cp}). ^{31}P NMR ($CDCl_3$, δ, ppm) 49.35 (s). MS (ESI): m/z= 471.2 [M^+].

Tri-*tert*-butyl(6-ferrocenyl-6-oxohexyl) phosphonium bis(trifluoromethanesulfonyl)imide (5c). Yield 0.394 g (80%). ^{1}H NMR ($CDCl_3$, δ, ppm) 1.67 (d, $^3J_{HP}$ = 13.41 Hz, 27H, P(C(CH_3)$_3$)$_3$), 1.81 (m, 4H, CH_2), 2.01 (m, 2H, CH_2), 2.32 (m, 2H, P-C$\underline{H_2}$), 2.83 (m, 2H, C(O)CH_2), 4.27 (s, 5H, H_{Cp}), 4.56 (s, 2H, H_{Cp}), 4.87 (s, 2H, H_{Cp}). ^{31}P NMR ($CDCl_3$, δ, ppm) 49.49 (c). MS (ESI): m/z= 485.2 [M^+].

Tri-*tert*-butyl(11-ferrocenyl-11-oxoundecyl) phosphonium bis(trifluoromethanesulfonyl)imide (5d). Yield. 0.579 g (82%). ^{1}H NMR ($CDCl_3$, δ, ppm) 1.35 (m, 12H, CH_2), 1.57 (m, 2H, CH_2), 1.65 (d, $^3J_{HP}$ = 13.86 Hz, 27H, P(C(CH_3)$_3$)$_3$), 1.91 (m, 2H, CH_2), 2.22 (m, 2H, P-CH_2), 2.72 (t, $^3J_{HH}$ = 7.17 Hz, 2H, C(O)CH_2), 4.22 (s, 5H, H_{Cp}), 4.52 (s, 2H, H_{Cp}), 4.80 (s, 2H, H_{Cp}). ^{31}P NMR ($CDCl_3$, δ, ppm) 49.64 (s). MS (ESI): m/z= 555.3 [M^+].

General procedure for the synthesis of 6a-6d.

Tert-Butylamine borane (6 eq.) was added to a suspension of anhydrous $AlCl_3$ (3 eq.) in dry CH_2Cl_2 (40 ml) at 0 °C under an inert gas atmosphere. The resulting mixture was stirred at the same temperature for 1 h. The solution of bromoalkanoyl ferrocene (1 eq.) in CH_2Cl_2 was added dropwise. The resulting solution was stirred overnight. The initially purple colour of the solution changes to brown during the reduction process. This indicates the end of the reaction. The reaction mixture was cooled to 0 °C and water (20 ml) was added dropwise to remove excess of the reducing agents. More H_2O (20 ml) was added and the solution was stirred for 30 minutes. The phases were separated and the aqueous phase was extracted with CH_2Cl_2 until the organic extract was colourless. The combined organic extracts were sequentially washed with sat. $NaHCO_3$ (2 x 20 ml), H_2O (20 ml) and brine (20 ml). The organic phase was dried over $MgSO_4$, all products were dried in vacuo after purification.

1-(3-bromopropyl)-ferrocene (6a). ^1H NMR ($CDCl_3$, δ, ppm) 2.04 (t, $^3J_{HP}$ = 7.50 Hz, 2H, CH_2), 2.50 (t, 2H, $^3J_{HP}$ = 7.50 Hz, 2H, CH_2), 3.42 (t, $^3J_{HH}$ = 6.50 Hz, 2H, CH_2Br), 4.10 (s, 9H, H_{Cp}).

1-(5-bromopentyl)-ferrocene (6b). ^1H NMR ($CDCl_3$, δ, ppm) 1.51 (m, 4H, CH_2), 1.90 (m, 2H, CH_2), 2.37 (m, 2H, CH_2), 3.43 (t, $^3J_{HH}$ = 6.59 Hz, 2H, CH_2Br), 4.11 (s, 9H, H_{Cp}).

1-(6-bromohexyl)-ferrocene (6c). ^1H NMR ($CDCl_3$, δ, ppm) δ = 1.40 (m, 2H, CH_2), 1.48 (m, 4H, CH_2), 1.89 (t, $^3J_{HH}$ = 6.44 Hz, 2H, CH_2), 2,15 (m, 2H, CH_2), 3.44 (t, $^3J_{HH}$ = 6.53 Hz, 2H, CH_2Br), 4.42 (s, 9H, H_{Cp}).

1-(11-bromoundecyl)-ferrocene (6d). ^1H NMR ($CDCl_3$, δ, ppm) 1.47 (m, 16H, CH_2), 1.87 (t, $^3J_{HH}$ = 7.43 Hz, 2H, CH_2), 2.34 (t, $^3J_{HH}$ = 8.05 Hz, 2H, CH_2), 3.43 (t, $^3J_{HH}$ = 6.81 Hz, 2H, CH_2Br), 4.11 (m, 9H, H_{Cp}).

General procedure for the synthesis of 7a-7d.

Tri-tert-butylphosphine was dissolved in dry DMF (5 ml) and an equivalent amount of bromoalkyl ferrocene was added. The reaction mixture was stirred at 55 °C for 5 - 20 hours. The solvent was removed in vacuo and the product was stirred with 3 portions of 20 ml of petroleum ether and 3 portions of 20 ml of diethyl ether. The precipitate was filtered out and the solvent residues were removed in vacuo.

Tri-*tert*-butyl(3-ferrocenylpropyl) phosphonium bromide (7a). ^1H NMR ($CDCl_3$, δ, ppm) 1.62 (d, $^3J_{HP}$ = 13.63 Hz, 27H, $P(C(CH_3)_3)_3$), 2.10 (s, 2H, CH_2), 2.73 (m,

2H, CH_2), 2.83 (m, 2H, CH_2), 4.09 (s, 5H, H_{Cp}), 4.13 (s, 2H, H_{Cp}), 4.16 (s, 2H, H_{Cp}). ^{31}P NMR ($CDCl_3$, δ, ppm) 50.13 (s). ^{13}C NMR ($CDCl_3$, δ, ppm) 18.10 (d, $^{1}J_{PC}$ = 35.63 Hz, P-CH_2), 27.54 (d, $^{3}J_{PC}$ = 5.77 Hz, P-CH_2-CH_2-$\underline{C}H_2$), 30.57 (s, C($\underline{C}H_3$)$_3$), 31.41 (d, $^{2}J_{PC}$ = 14.53 Hz, P-CH_2-$\underline{C}H_2$), 39.81 (d, $^{1}J_{PC}$ = 29.48 Hz, P-($\underline{C}$(CH_3))$_3$), 68.29 (s, C_p), 69.14 (s, C_p), 69.29 (s, C_p), 87.02 (s, C_p). MS (ESI): m/z = 429.32 [M^+].

Tri-*tert*-butyl(5-ferrocenylpentyl) phosphonium bromide (7b). ^{1}H NMR ($CDCl_3$, δ, ppm) 1.41 (m, 2H, CH_2), δ = 1.65 (d, $^{3}J_{HP}$ = 14.12 Hz, 27H, P(C(CH_3)$_3$)$_3$), 1.86 (m, 4H, CH_2), 2.23 (s, 2H, CH_2), 2.56 (s, 2H, CH_2), 4.33 (m, 9H, H_{Cp}). ^{31}P NMR ($CDCl_3$, δ, ppm) 49.53 (s). ^{13}C NMR ($CDCl_3$, δ, ppm) 19.51 (d, $^{1}J_{PC}$ = 34.47 Hz, P-CH_2), 25.79 (d, $^{3}J_{PC}$ = 6.42 Hz, P-CH_2-CH_2-$\underline{C}H_2$), 30.63 (s, C($\underline{C}H_3$)$_3$), 32.20 (d, $^{2}J_{PC}$ = 12.66 Hz, P-CH_2-$\underline{C}H_2$), 39.84 (d, $^{1}J_{PC}$ = 29.40 Hz, P-($\underline{C}$(CH_3))$_3$), 67.64 (s, C_p), 68.68 (s, C_p), 69.03 (s, C_p), 87.30 (s, C_p). MS (ESI): m/z = 457.36 [M^+].

Tri-*tert*-butyl(6-ferrocenylhexyl) phosphonium bromide (7c). ^{1}H NMR ($CDCl_3$, δ, ppm) 1.43 (m, 4H, CH_2), 1.62 (d, $^{3}J_{HP}$ = 13.92 Hz, 27H, P(C(CH_3)$_3$)$_3$), 1.86 (m, 4H, CH_2), 2.11 (m, 2H, CH_2), 2.21 (m, 2H, CH_2), 4.40 (s, 9H, H_{Cp}). ^{31}P NMR ($CDCl_3$, δ, ppm) 49.64 (s). ^{13}C NMR ($CDCl_3$, δ, ppm) 19.50 (d, $^{1}J_{PC}$ = 34.48 Hz, P-CH_2), 25.60 (d, $^{3}J_{PC}$ = 6.23 Hz, P-CH_2-CH_2-$\underline{C}H_2$), 29.54 (s, CH_2), 29.99 (s, CH_2), 30.61 (s, C($\underline{C}H_3$)$_3$), 32.1 (d, $^{2}J_{PC}$ = 12.47 Hz, P-CH_2-$\underline{C}H_2$), 39.80 (d, $^{1}J_{PC}$ = 29.34 Hz, P-($\underline{C}$(CH_3))$_3$), 67.56 (s, C_p), 68.64 (s, C_p), 69.01 (s, C_p), 89.61 (s, C_p). MS (ESI): m/z = 471.38 [M^+].

Tri-*tert*-butyl(11-ferrocenylundecyl) phosphonium bromide (7d). ^{1}H NMR ($CDCl_3$, δ, ppm) 1.31 (m, 12H, CH_2), 1.46 (m, 2H, CH_2), 1.72 (d, $^{3}J_{HP}$ = 12.99 Hz, 27H, P(C(CH_3)$_3$)$_3$), 1.99 (m, 2H, CH_2), 2.19 (m, 2H, P-C$\underline{H_2}$), 2.62 (m, 2H, CH_2), 4.29 (m, 9H, H_{Cp}). ^{31}P NMR ($CDCl_3$, δ, ppm) 49.13 (s). ^{13}C NMR ($CDCl_3$, δ, ppm) 18.54 (d, P-CH_2), 24.50 (s, CH_2), 24.90 (d, $^{3}J_{PC}$ = 6.41 Hz, P-CH_2-CH_2-$\underline{C}H_2$), 29.02 (s, CH_2), 29.19 (s, CH_2), 29.26 (s, CH_2), 29.33 (s, CH_2), 29.79 (s, C($\underline{C}H_3$)$_3$), 31.63 (d, P-CH_2-$\underline{C}H_2$), 39.14 (d, P-($\underline{C}$(CH_3))$_3$), 69.26 (s, C_p), 69.71 (s, C_p), 72.21 (s, C_p), 79.09 (s, C_p). MS (ESI): m/z = 541.49 [M^+].

General procedure for the synthesis of 8a-8d.

Tri-*tert*-butyl(3-ferrocenylpropyl) phosphonium tetrafluoroborate (8a). ^{1}H NMR ($CDCl_3$, δ, ppm) 1.36 (s, 2H, CH_2), 1.58 (d, $^{3}J_{HP}$ = 12.36 Hz, 27H, P(C(CH_3)$_3$)$_3$), 2.08 (m, 2H, CH_2), 2.25 (m, 2H, CH_2), 4.59 (m, 9H, H_{Cp}). ^{31}P NMR ($CDCl_3$, δ, ppm) 49.84 (s). ^{13}C NMR ($CDCl_3$, δ, ppm) 17.67 (d, $^{1}J_{PC}$ = 36.20 Hz, P-CH_2), 27.42 (d, $^{3}J_{PC}$ =

6.57 Hz, P-CH_2-CH_2-$\underline{C}H_2$), 3.29 (s, C($\underline{C}H_3$)$_3$), 31.24 (d, $^2J_{PC}$ = 13.23 Hz, P-CH_2-$\underline{C}H_2$), 39.76 (d, $^1J_{PC}$ = 29.47 Hz, P-($\underline{C}$(CH_3))$_3$), 68.36 (s, C_p), 69.13 (s, C_p), 69.34 (s, C_p), 89.90 (s, C_p).

Tri-*tert*-butyl(5-ferrocenylpentyl) phosphonium tetrafluoroborate (8b). ^{1}H NMR ($CDCl_3$, δ, ppm) 1.41 (m, 4H, CH_2), 1.60 (d, $^3J_{HP}$ = 13.19 Hz, 27H, P(C(CH_3)$_3$)$_3$), 1.86 (m, 2H, CH_2), 2.08 (m, 2H, CH_2), 2.20 (m, 2H, CH_2), 4.43 (m, 9H, H_{Cp}). ^{31}P NMR ($CDCl_3$, δ, ppm) 49.7 (s). ^{13}C NMR ($CDCl_3$, δ, ppm) 18.96 (d, $^1J_{PC}$ = 34.92 Hz, P-CH_2), 25.59 (d, $^3J_{PC}$ = 6.60 Hz, P-CH_2-CH_2-$\underline{C}H_2$), 30.36 (s, C($\underline{C}H_3$)$_3$), 31.92 (d, $^2J_{PC}$ = 12.80 Hz, P-CH_2-$\underline{C}H_2$), 39.79 (d, $^1J_{PC}$ = 29.33 Hz, P-($\underline{C}$(CH_3))$_3$), 69.07 (s, C_p), 70.51 (s, C_p), 70.76 (s, C_p), 87.20 (s, C_p).

Tri-*tert*-butyl(6-ferrocenylhexyl) phosphonium tetrafluoroborate (8c). ^{1}H NMR ($CDCl_3$, δ, ppm) 1.41 (m, 4H, CH_2), 1.60 (d, $^3J_{HP}$ = 13.19 Hz, 27H, P(C(CH_3)$_3$)$_3$), 1.86 (m, 4H, CH_2), 2.08 (m, 2H, CH_2), 2.20 (m, 2H, CH_2), 4.45 (m, 9H, H_{Cp}). ^{31}P NMR ($CDCl_3$, δ, ppm) 49.75 (s). ^{13}C NMR ($CDCl_3$, δ, ppm) 18.76 (d, $^1J_{PC}$ = 35.10 Hz, P-CH_2), 25.01 (d, $^3J_{PC}$ = 5.71 Hz, P-CH_2-CH_2-$\underline{C}H_2$), 28.89 (s, CH_2), 29.22 (s, CH_2), 30.02 (s, C($\underline{C}H_3$)$_3$), 30.49 (s, CH_2), 31.54 (d, $^2J_{PC}$ = 11.70 Hz, P-CH_2-$\underline{C}H_2$), 39.31 (d, $^1J_{PC}$ = 29.47 Hz, P-($\underline{C}$(CH_3))$_3$), 69.94 (s, C_p), 71.00 (s, C_p), 72.00 (s, C_p)

Tri-*tert*-butyl(11-ferrocenyundecyl) phosphonium tetrafluoroborate (8d). ^{1}H NMR ($CDCl_3$, δ, ppm) 1.29 (m, 14H, CH_2), 1.67 (d, $^3J_{HP}$ = 13.24 Hz, 27H, P(C(CH_3)$_3$)$_3$) m, 2H, CH_2) 1.89 (m, 2H, CH_2), 2.30 (m, 2H, CH_2), 2.57 (m, 2H, CH_2), 4.07 (m, 9H, H_{Cp}) ^{31}P NMR ($CDCl_3$, δ, ppm) 49.5 (s). ^{13}C NMR ($CDCl_3$, δ, ppm) 19.56 (d, $^1J_{PC}$ = 34.06 Hz, P-CH_2), 25.78 (d, $^3J_{PC}$ = 6.44 Hz, P-CH_2-CH_2-$\underline{C}H_2$), 28.55 (s, CH_2), 29.88 (s, CH_2), 30.65 (s, C($\underline{C}H_3$)$_3$), 31.60 (s, CH_2), 32.42 (d, $^2J_{PC}$ = 12.37 Hz, P-CH_2-$\underline{C}H_2$), 39.87 (d, $^1J_{PC}$ = 29.39 Hz, P-($\underline{C}$(CH_3))$_3$), 67.51 (s, C_p), 68.60 (s, C_p), 69.01 (s, C_p), 90.16 (s, C_p).

$$Br(CH_2)_nCOOH \xrightarrow[n\,=\,2,\,4,\,5,\,10]{SOCl_2,\ CH_2Cl_2} \underset{\mathbf{1a\text{-}d}}{Br(CH_2)_nCOCl}$$

Scheme 1. ω-bromoacylchlorides synthesis

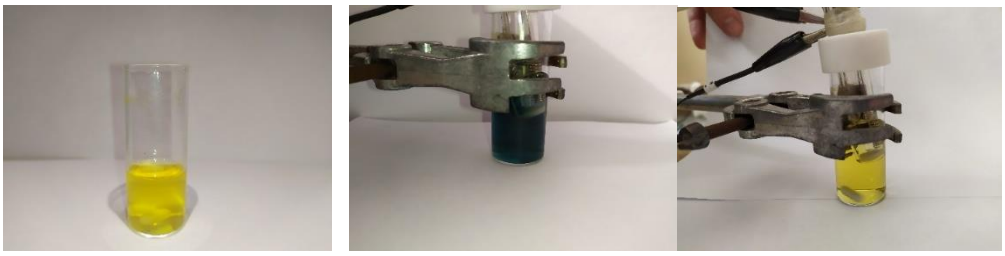

a b c

Figure S1. Photo of reversible redox process of solution of salt **7d WITHOUT an additional electrolyte**. Foto (a) CH_3CN with 0.1 M of 7d; Foto (b) CH_3CN with 0.1 M of 7d at the Ep=0.45V vs Ag/AgCl; Foto (c) CH_3CN with 0.1 M of 7d at the Ep=0.39V vs Ag/AgCl

Acquisition Parameter

Ion Source Type	ESI	Ion Polarity	Positive	Alternating Ion Polarity	off
Mass Range Mode	UltraScan	Scan Begin	50 m/z	Scan End	2800 m/z
Capillary Exit	140.0 V	n/a	n/a	Trap Drive	50.6
Accumulation Time	2563 µs	Averages	5 Spectra	n/a	n/a

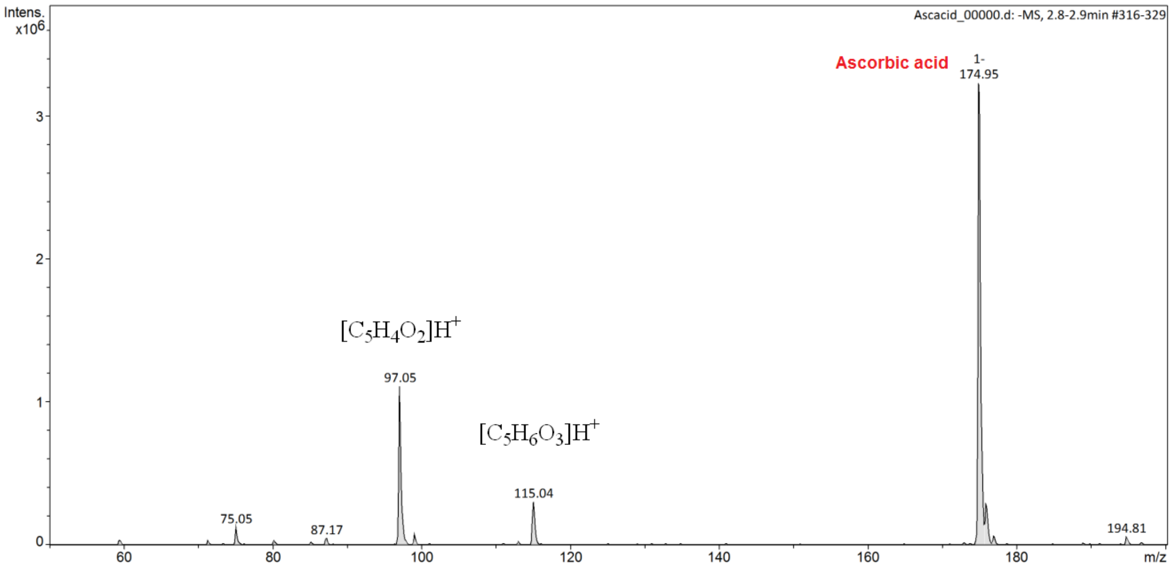

Acquisition Parameter

Ion Source Type	ESI	Ion Polarity	Positive	Alternating Ion Polarity	off
Mass Range Mode	UltraScan	Scan Begin	50 m/z	Scan End	2800 m/z
Capillary Exit	140.0 V	n/a	n/a	Trap Drive	50.6
Accumulation Time	2663 µs	Averages	5 Spectra	n/a	n/a

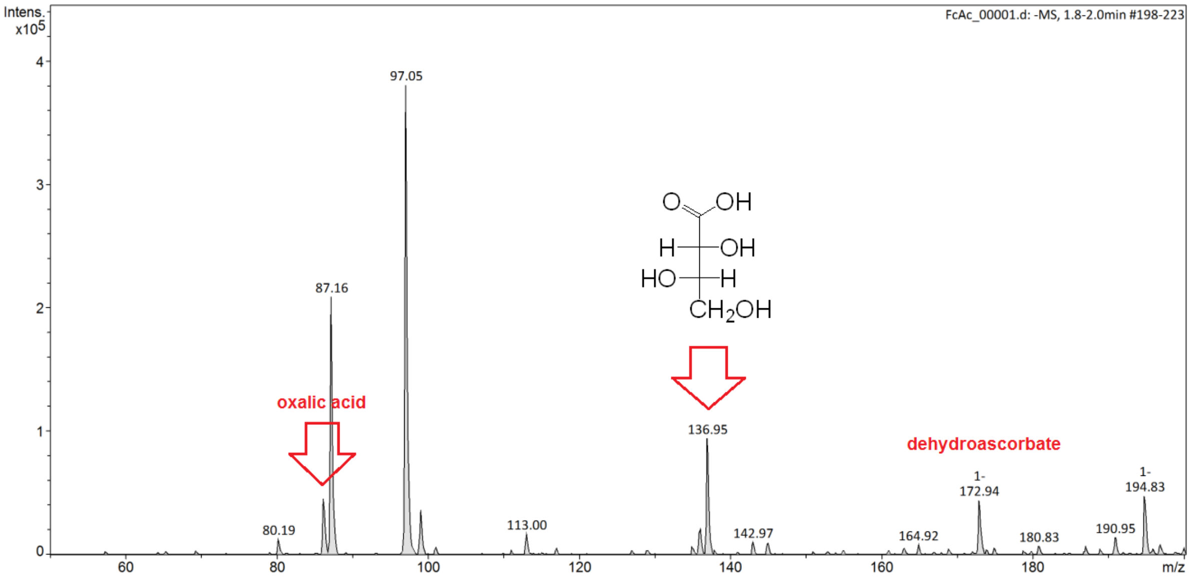

Figure S2. ESI(-) mass spectrum of an ascorbic acid solution, 10^{-5} M in EtOH before (top) and after (bottom) reaction.

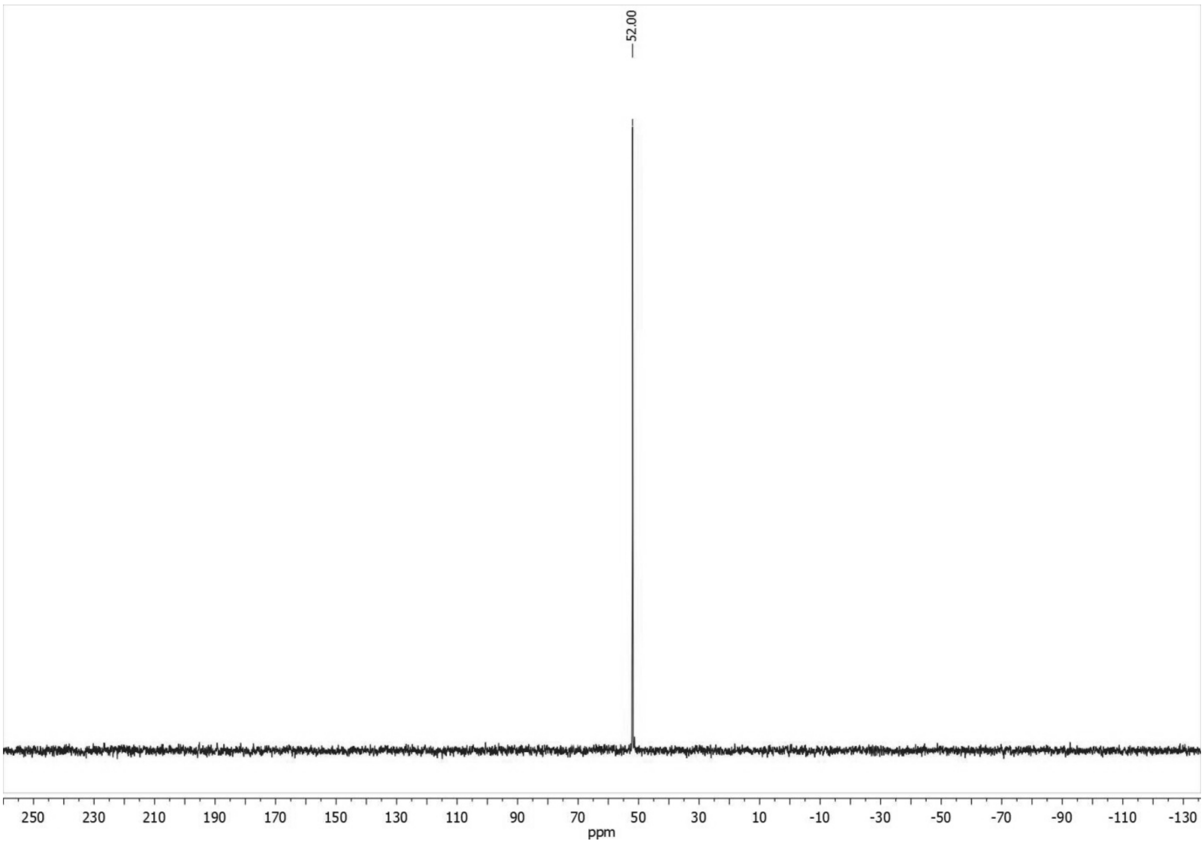

Figure S3. ^{31}P NMR spectra of the tri-*tert*-butyl(3-ferrocenyl-3-oxopropyl)phosphonium bromide **3a**

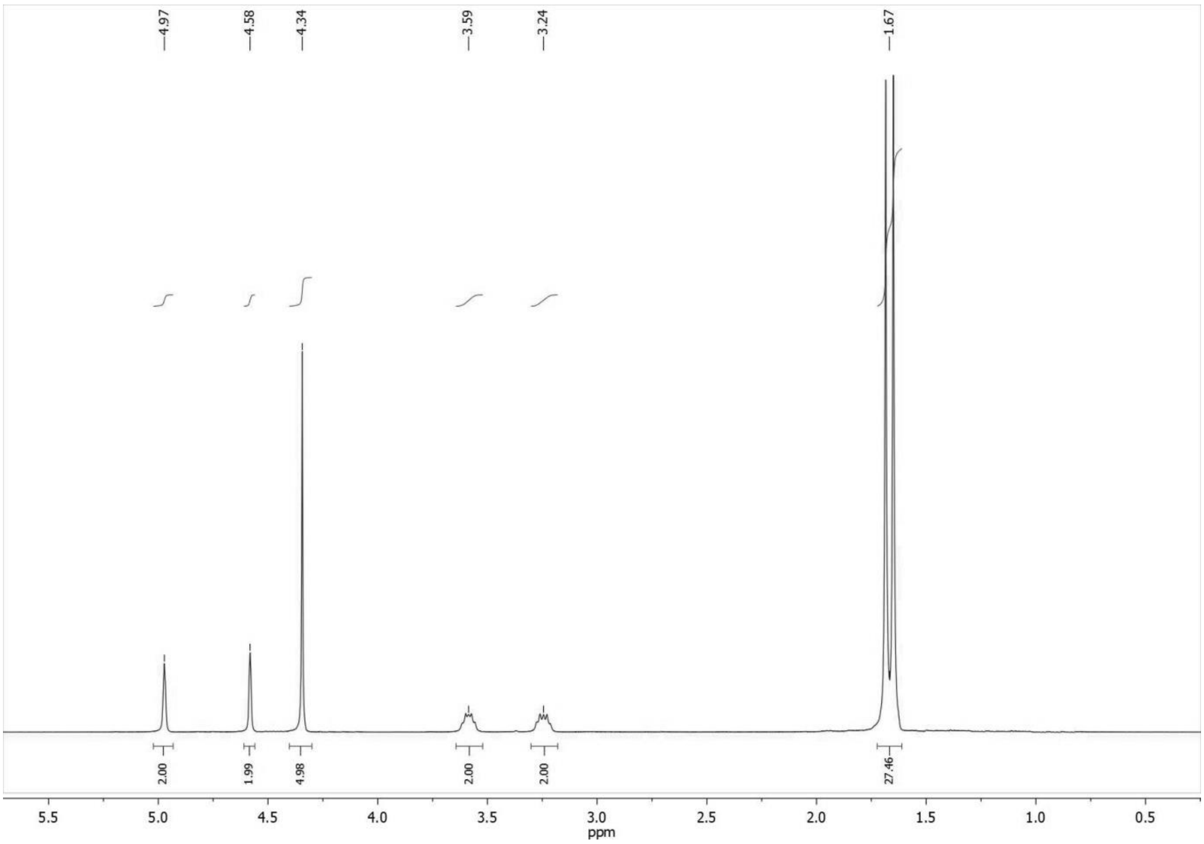

Figure S4. ^{1}H NMR spectra of the tri-*tert*-butyl(3-ferrocenyl-3-oxopropyl)phosphonium bromide **3a**

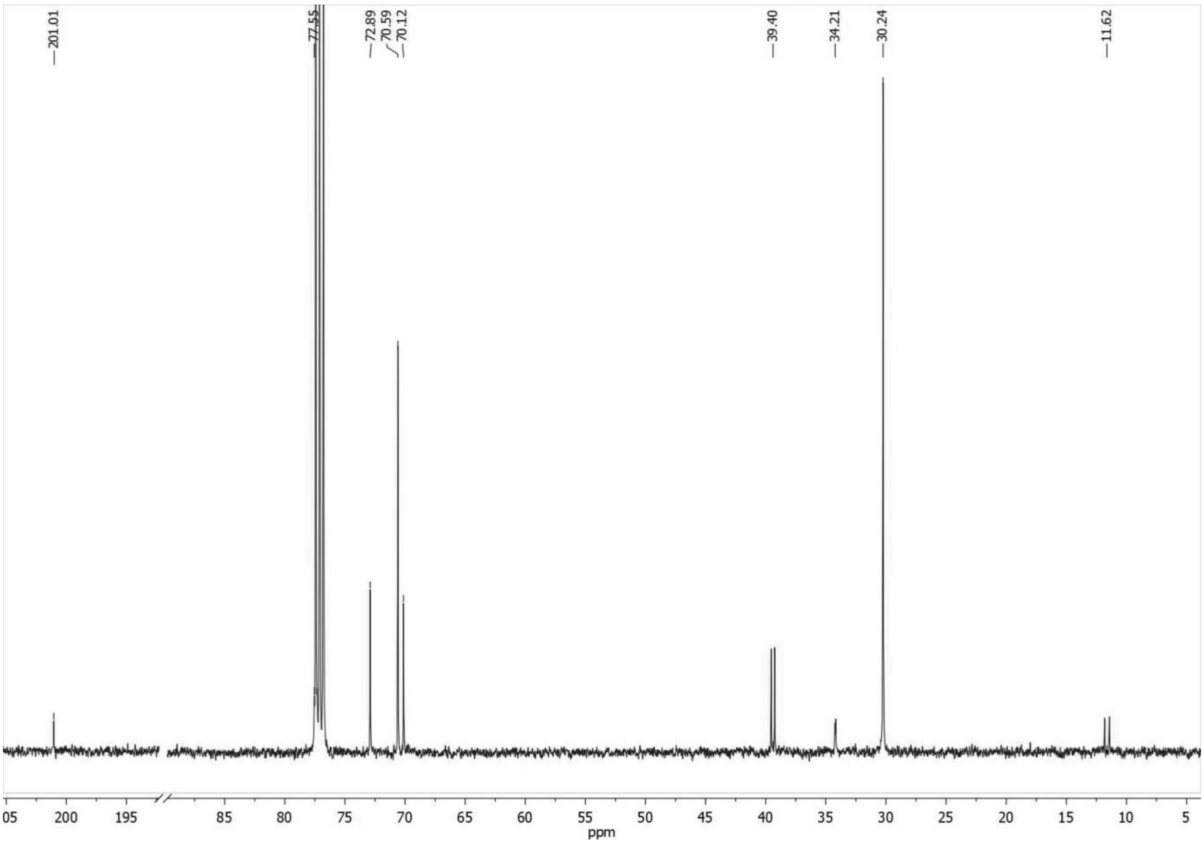

Figure S5. $^{13}C\{^{1}H\}$ NMR spectra of the tri-*tert*-butyl(3-ferrocenyl-3-oxopropyl)phosphonium bromide **3a**

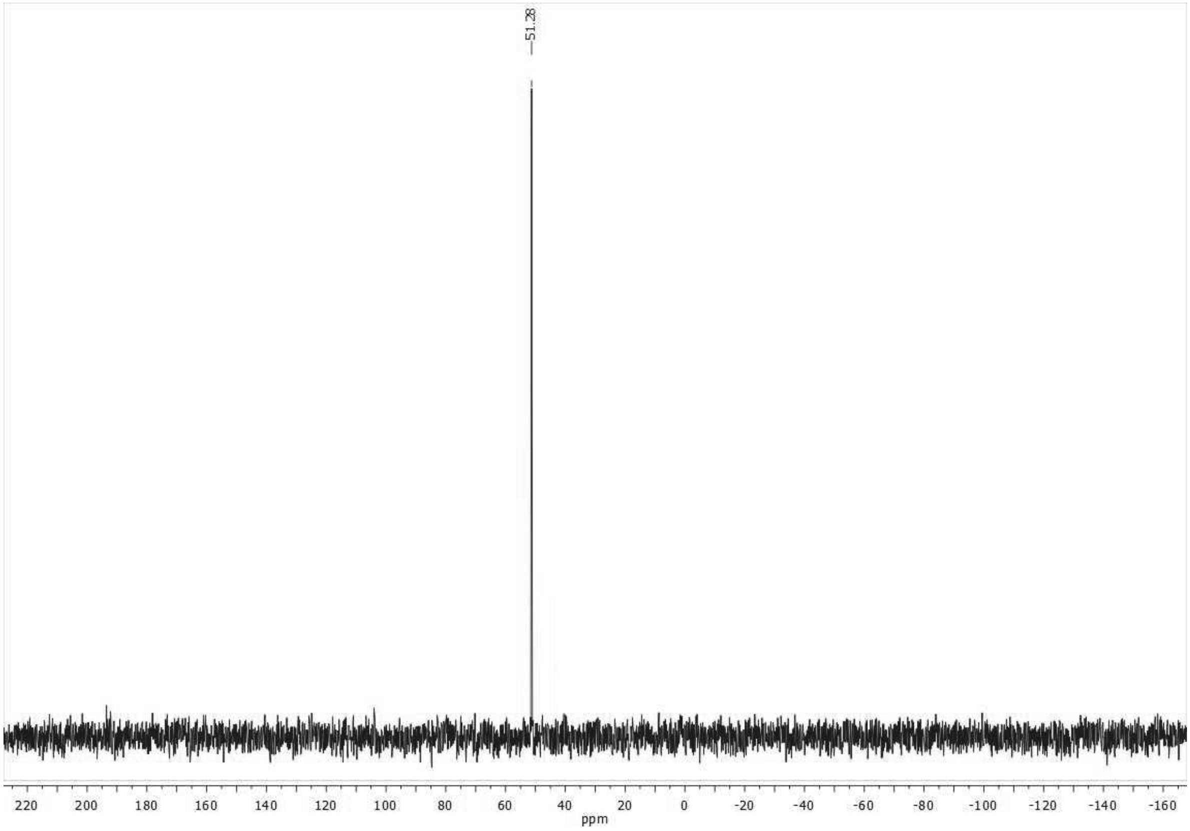

Figure S6. ^{31}P NMR spectra of the tri-*tert*-butyl(5-ferrocenyl-5-oxopentyl)phosphonium bromide **3b**

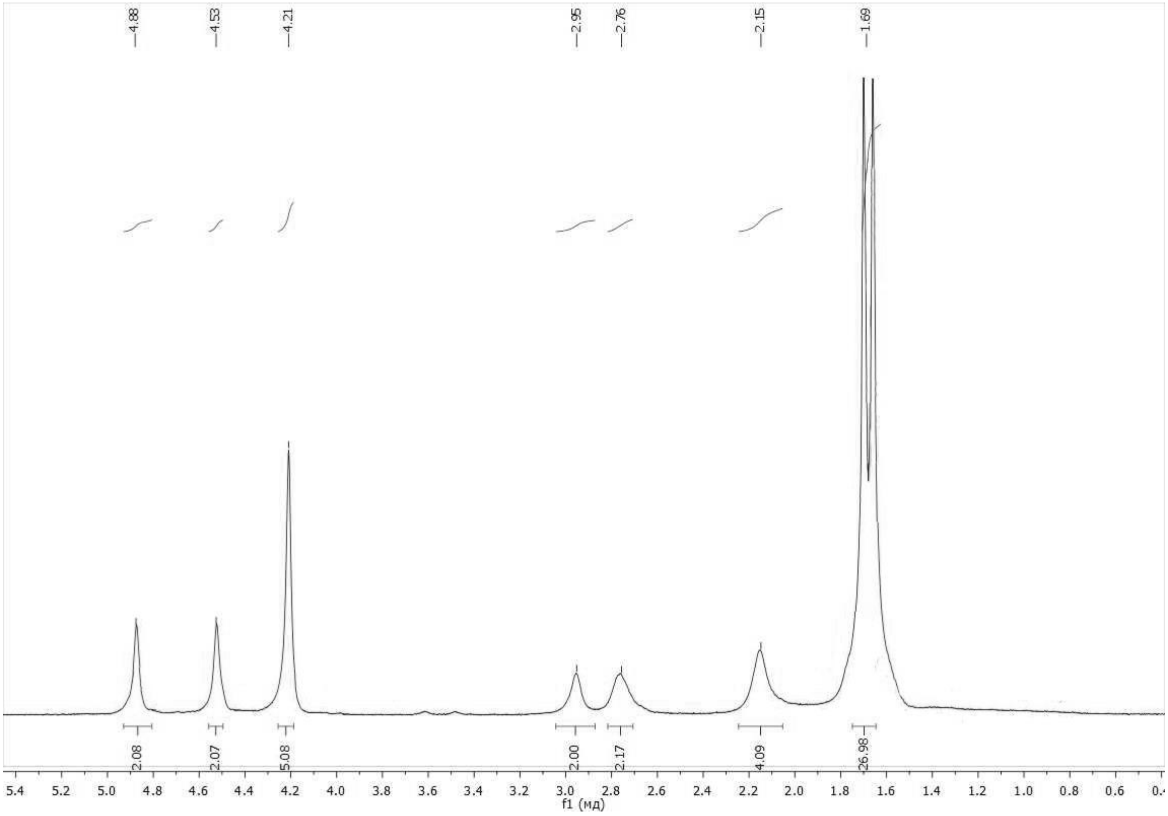

Figure S7. ^{1}H NMR spectra of the tri-*tert*-butyl(5-ferrocenyl-5-oxopentyl)phosphonium bromide **3b**

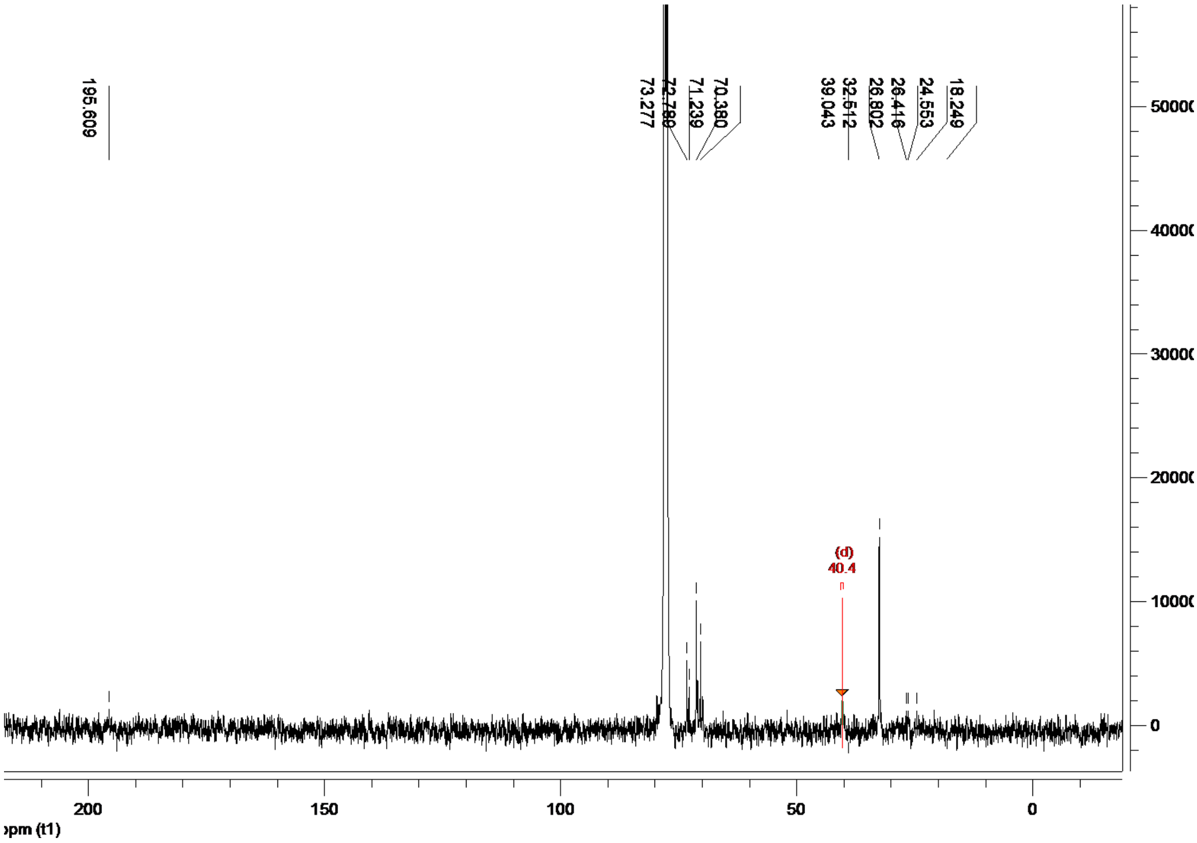

Figure S8. ^{13}C {^{1}H} NMR spectra of the tri-*tert*-butyl(5-ferrocenyl-5-oxopentyl)phosphonium bromide **3b**

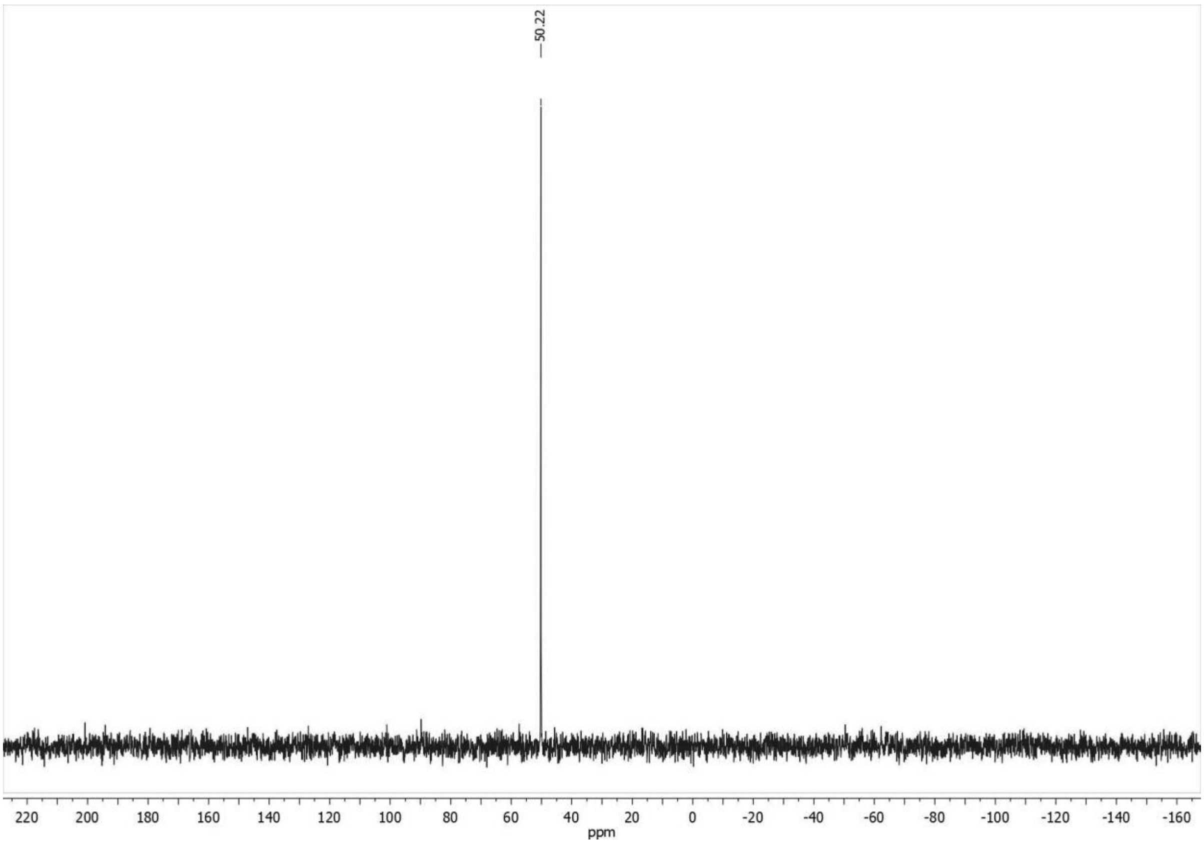

Figure S9. ^{31}P NMR spectra of the tri-*tert*-butyl(6-ferrocenyl-6-oxohexyl)phosphonium bromide **3c**

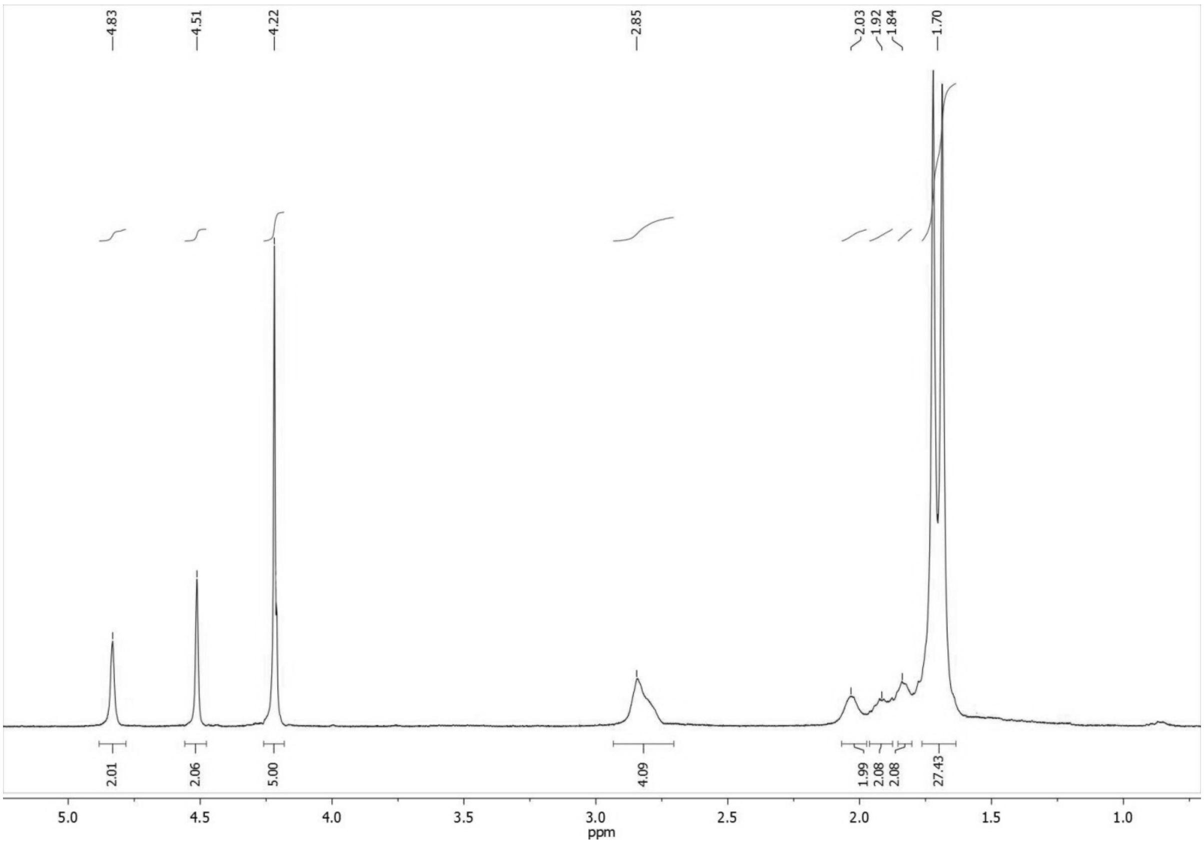

Figure S10. ^{1}H NMR spectra of the tri-*tert*-butyl(6-ferrocenyl-6-oxohexyl)phosphonium bromide **3c**

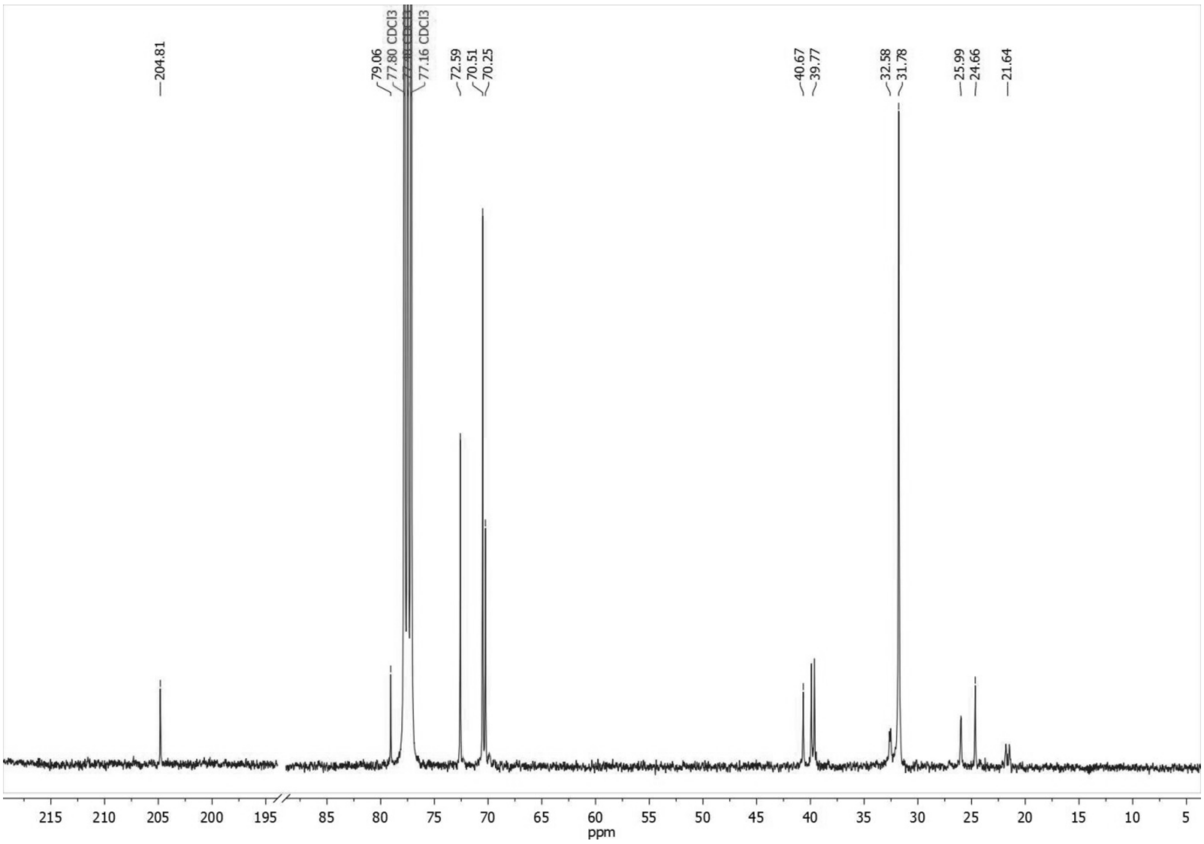

Figure S11. $^{13}C\{^1H\}$ NMR spectra of the tri-*tert*-butyl(6-ferrocenyl-6-oxohexyl)phosphonium bromide **3c**

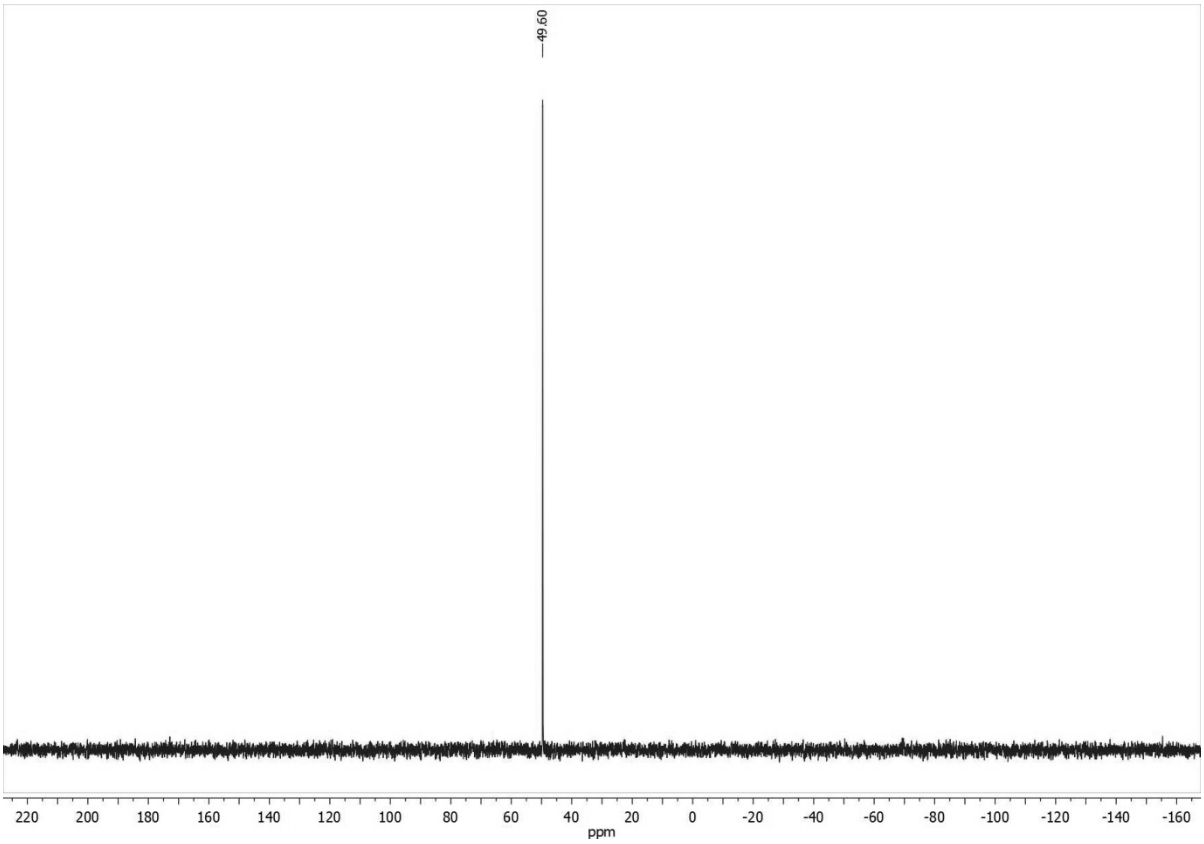

Figure S12. ^{31}P NMR spectra of the tri-*tert*-butyl(11-ferrocenyl-11-oxoundecyl)phosphonium bromide **3d**

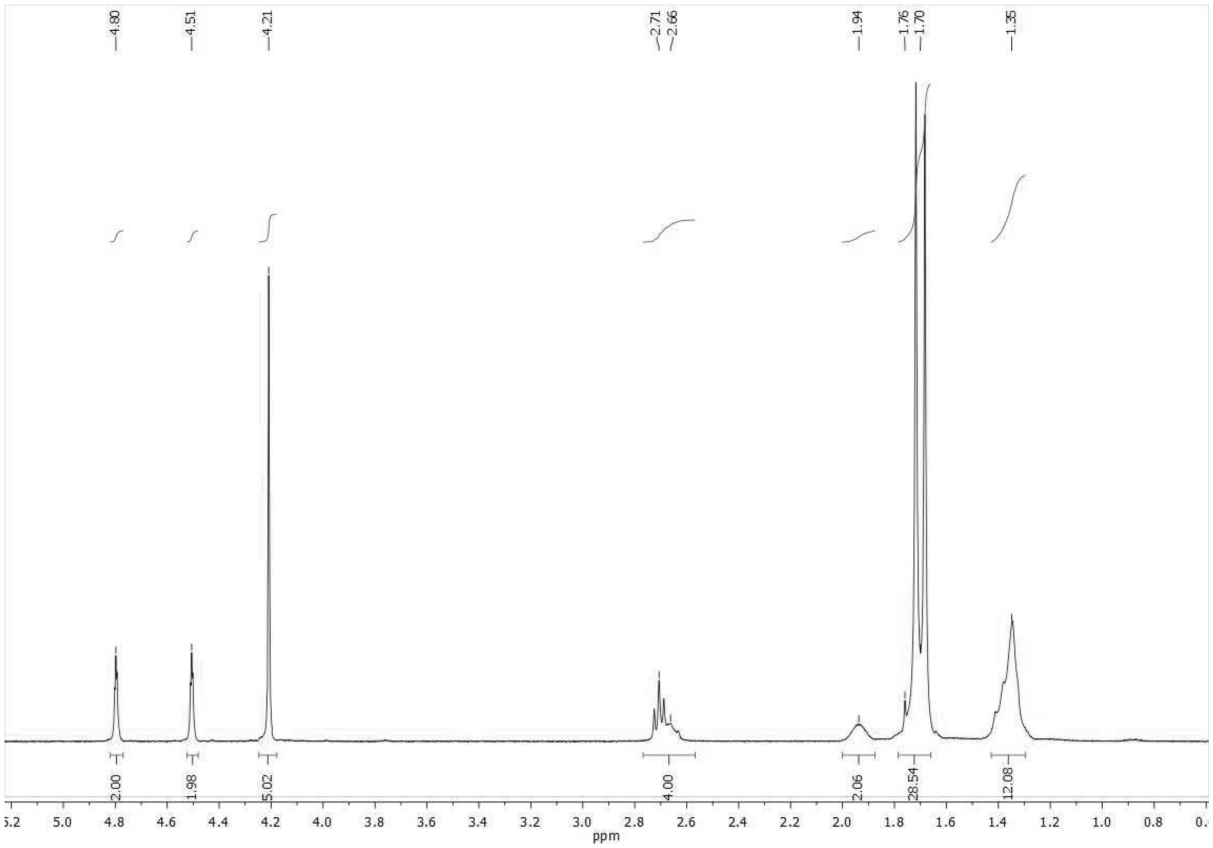

Figure S13. ^{1}H NMR spectra of the tri-*tert*-butyl(11-ferrocenyl-11-oxoundecyl)phosphonium bromide **3d**

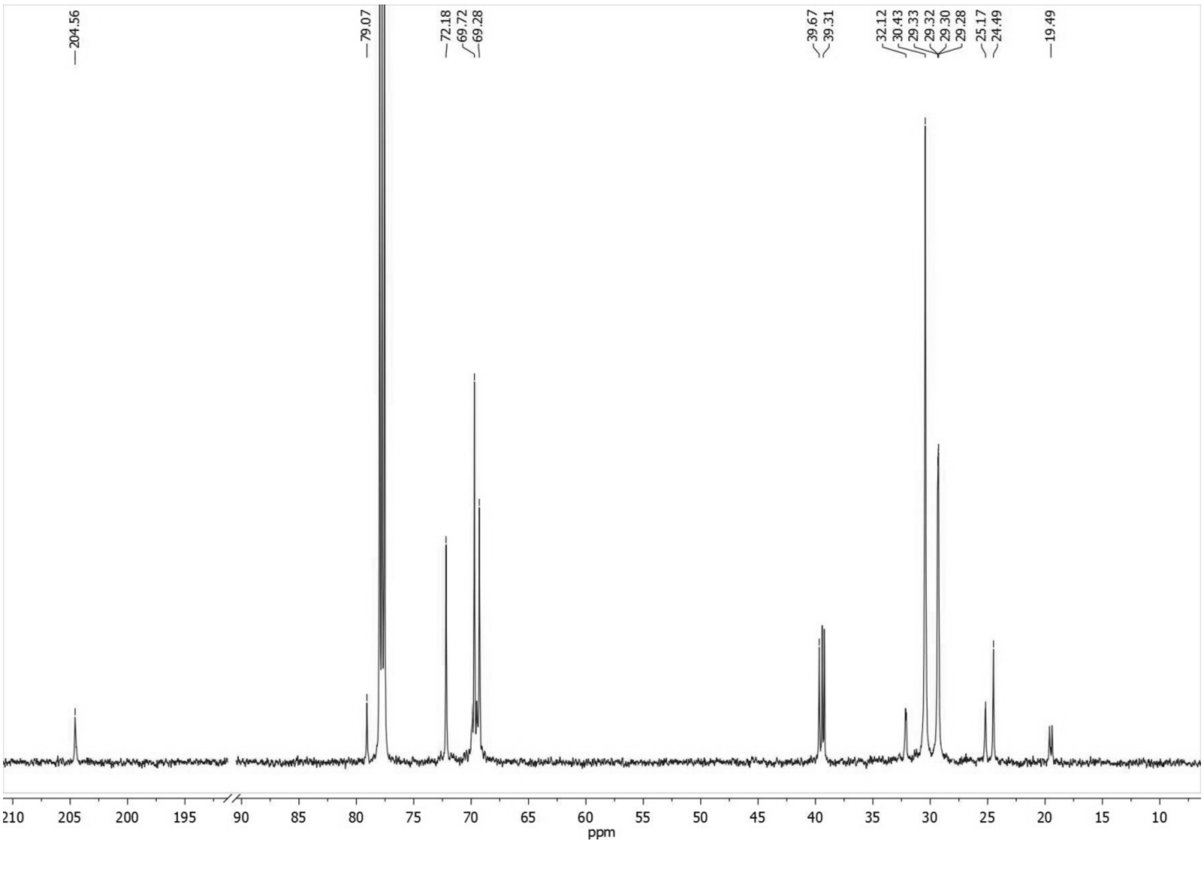

Figure S14. ^{13}C{^{1}H} NMR spectra of the tri-*tert*-butyl(11-ferrocenyl-11-oxoundecyl)phosphonium bromide **3d**

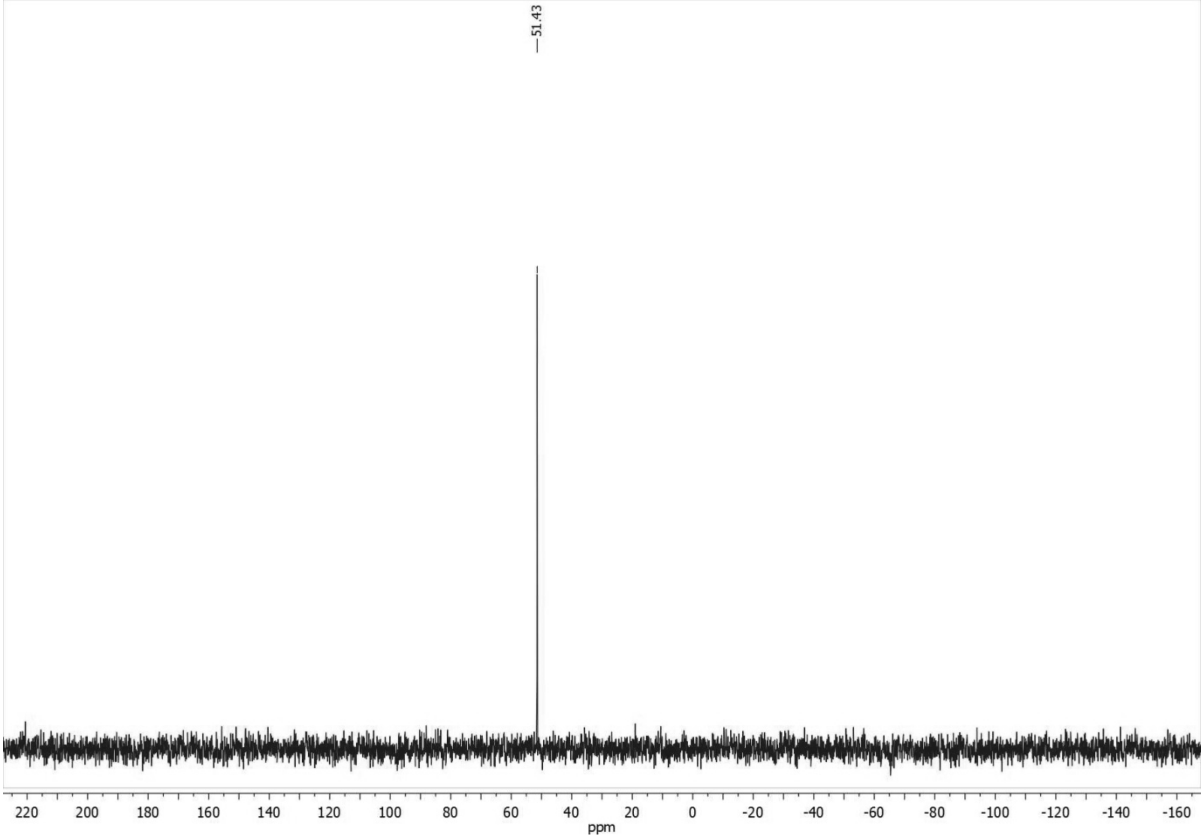

Figure S15. ^{31}P NMR spectra of the tri-*tert*-butyl(3-ferrocenyl-3-oxopropyl)phosphonium tetrafluoroborate **4a**

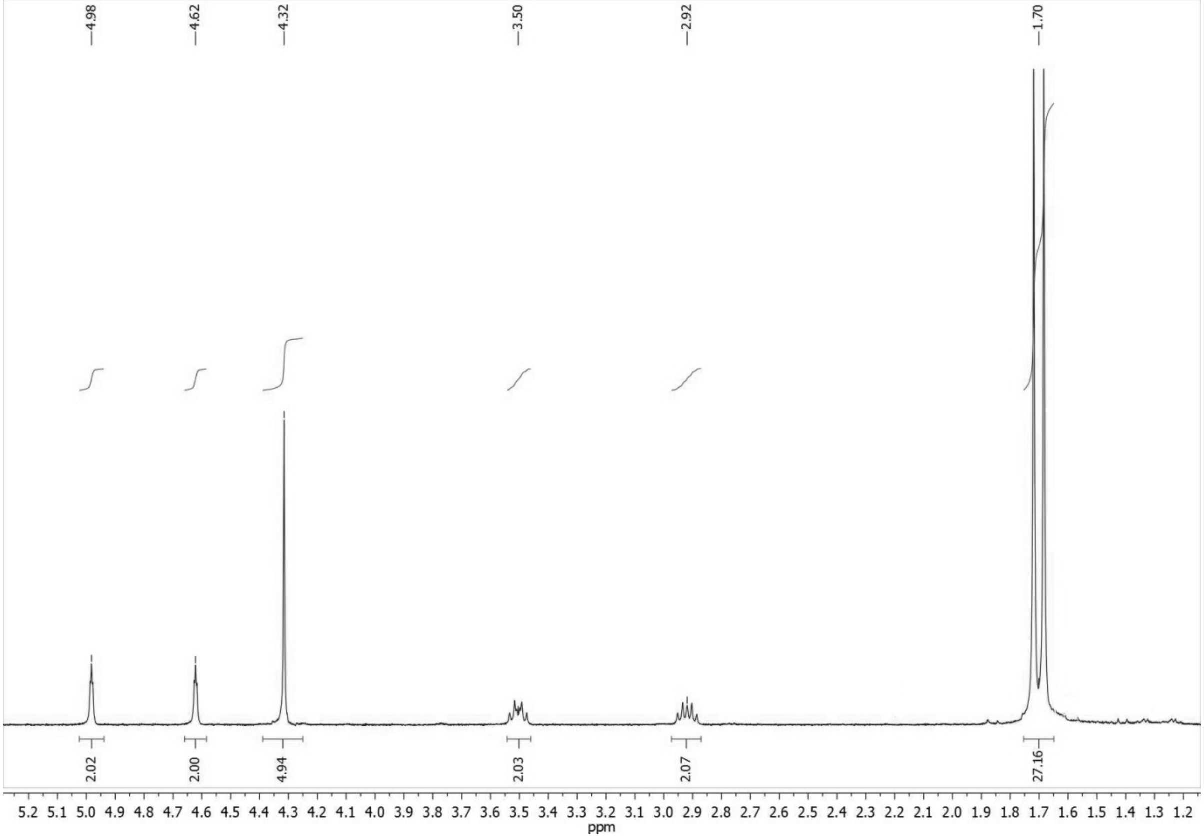

Figure S16. ^{1}H NMR spectra of the tri-*tert*-butyl(3-ferrocenyl-3-oxopropyl)phosphonium tetrafluoroborate **4a**

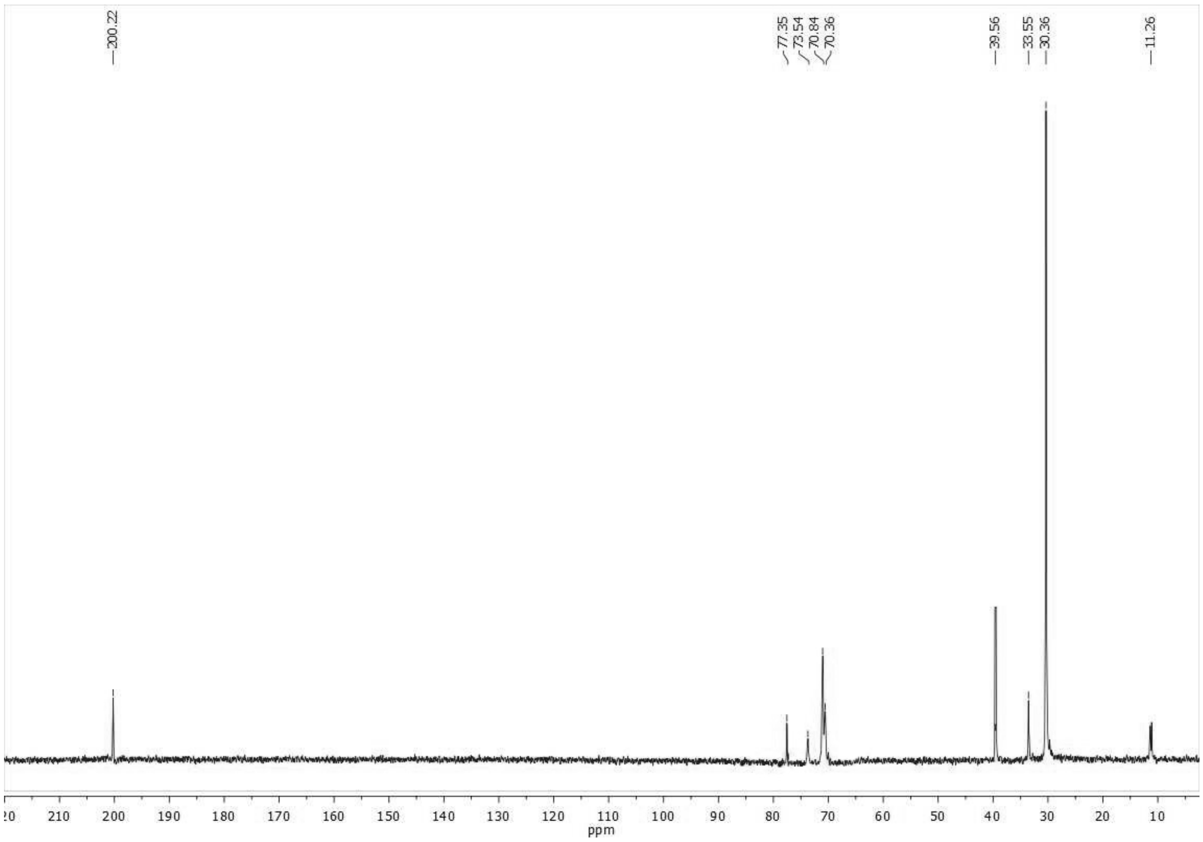

Figure S17. $^{13}C\{^{1}H\}$ NMR spectra of the tri-*tert*-butyl(3-ferrocenyl-3-oxopropyl)phosphonium tetrafluoroborate **4a**

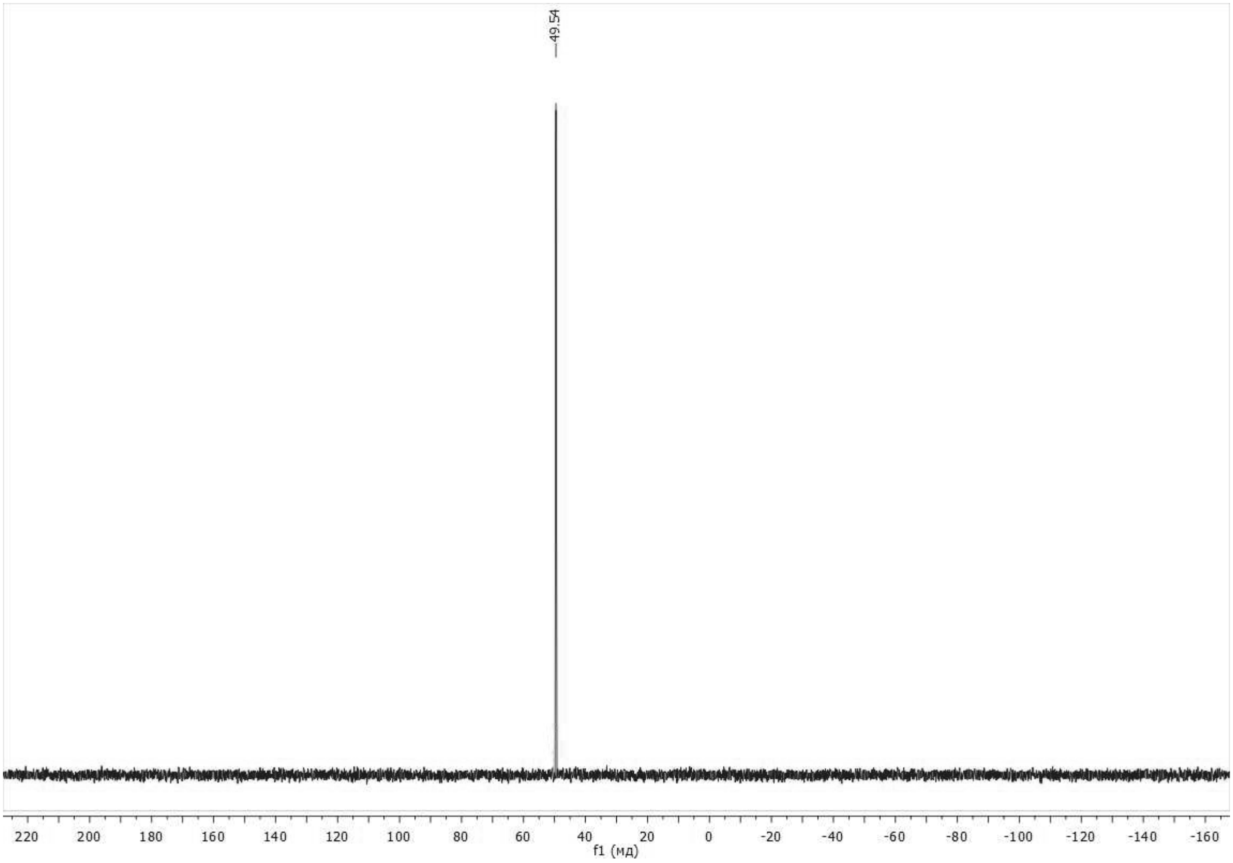

Figure S18. ^{31}P NMR spectra of the tri-*tert*-butyl(5-ferrocenyl-5-oxopentyl)phosphonium tetrafluoroborate **4b**

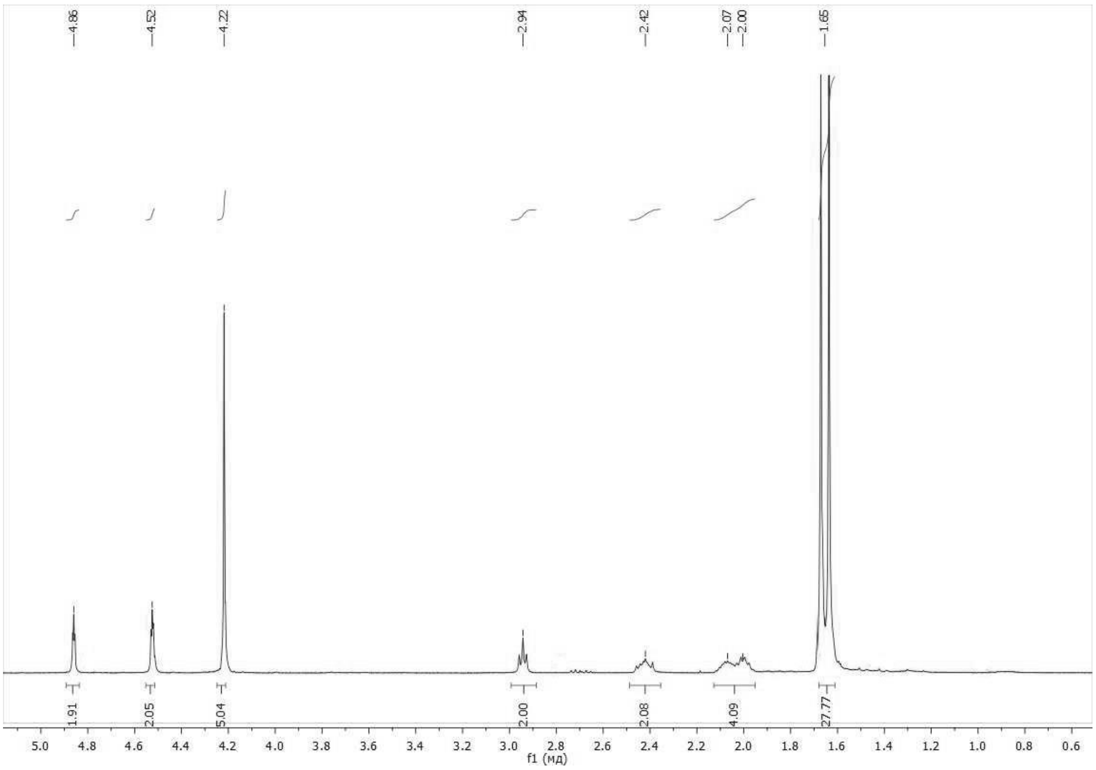

Figure S19. ^{1}H NMR spectra of the tri-*tert*-butyl(5-ferrocenyl-5-oxopentyl)phosphonium tetrafluoroborate **4b**

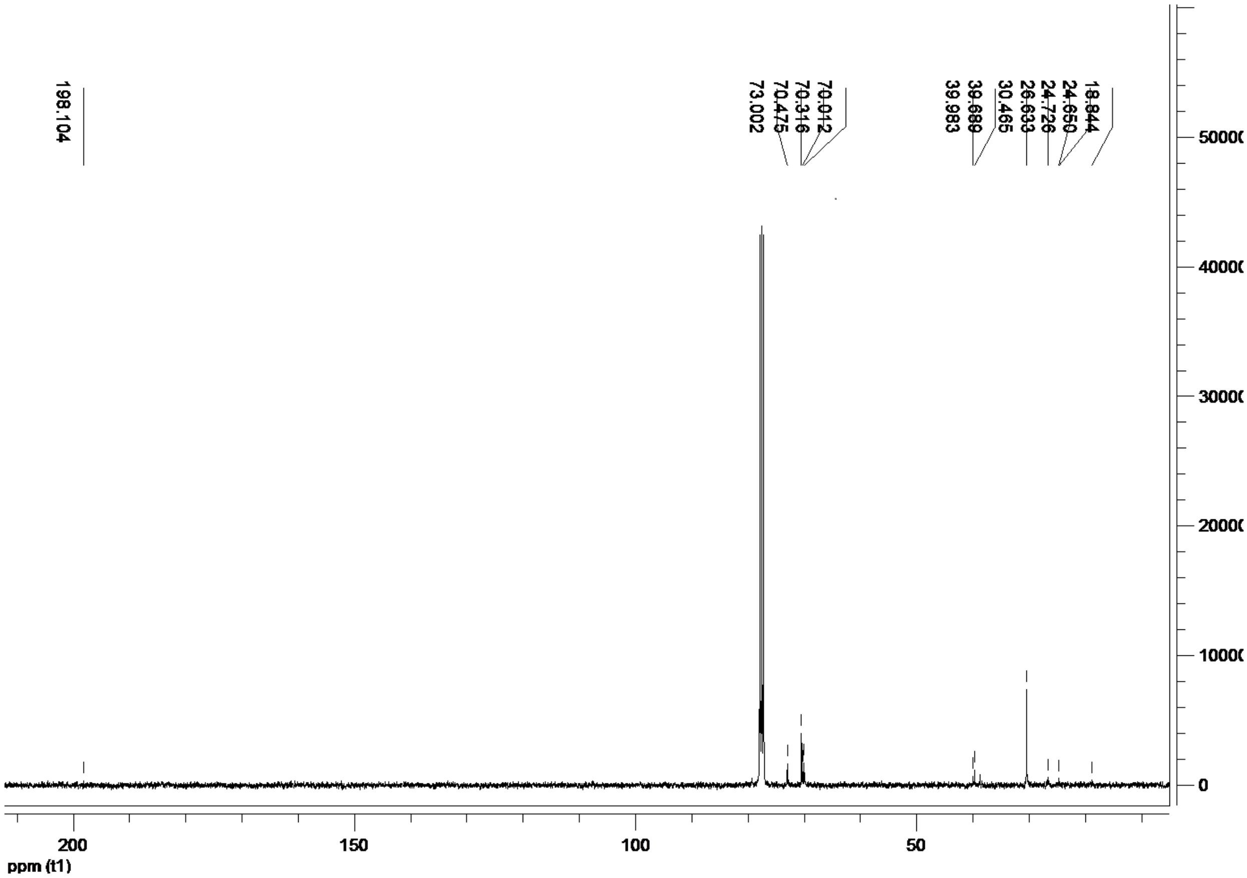

Figure S20. ^{13}C {^{1}H} NMR spectra of the tri-*tert*-butyl(5-ferrocenyl-5-oxopentyl)phosphonium tetrafluoroborate **4b**

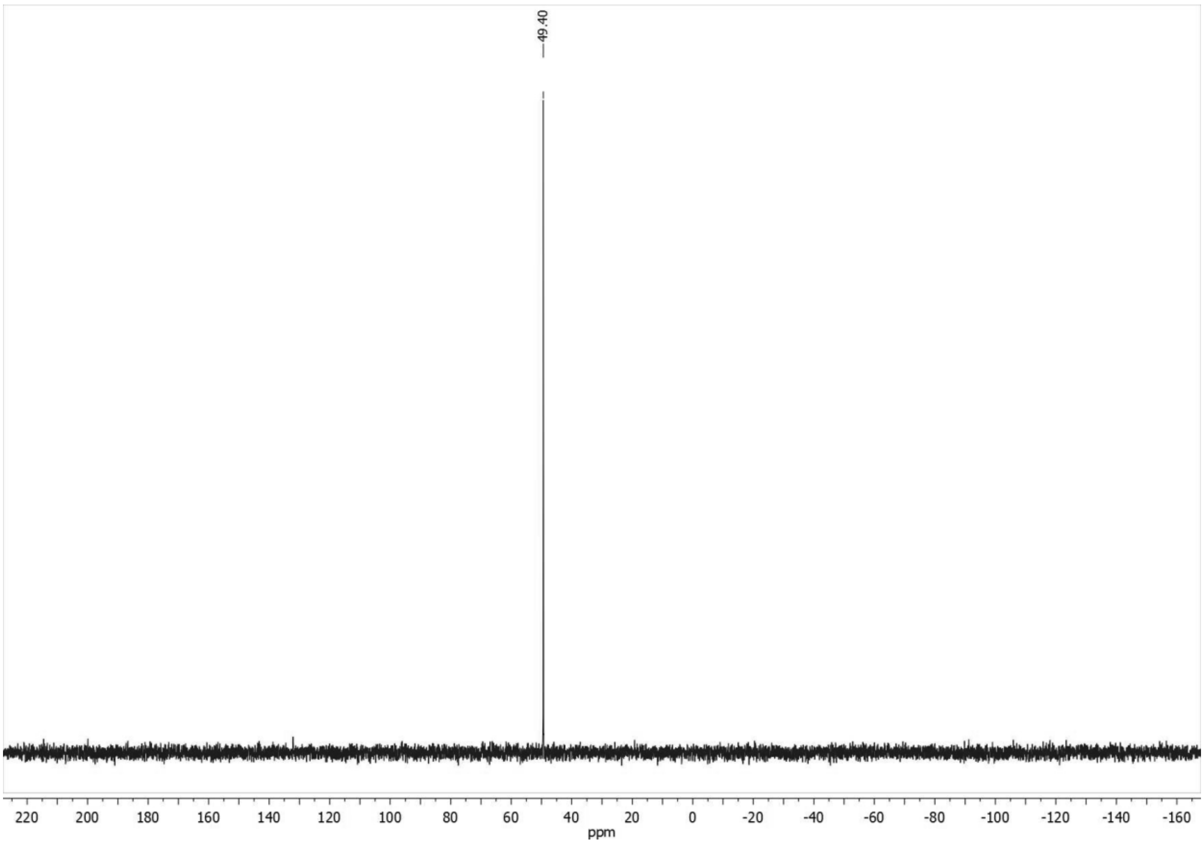

Figure S21. ^{31}P NMR spectra of the tri-*tert*-butyl(6-ferrocenyl-6-oxohexyl)phosphonium tetrafluoroborate **4c**

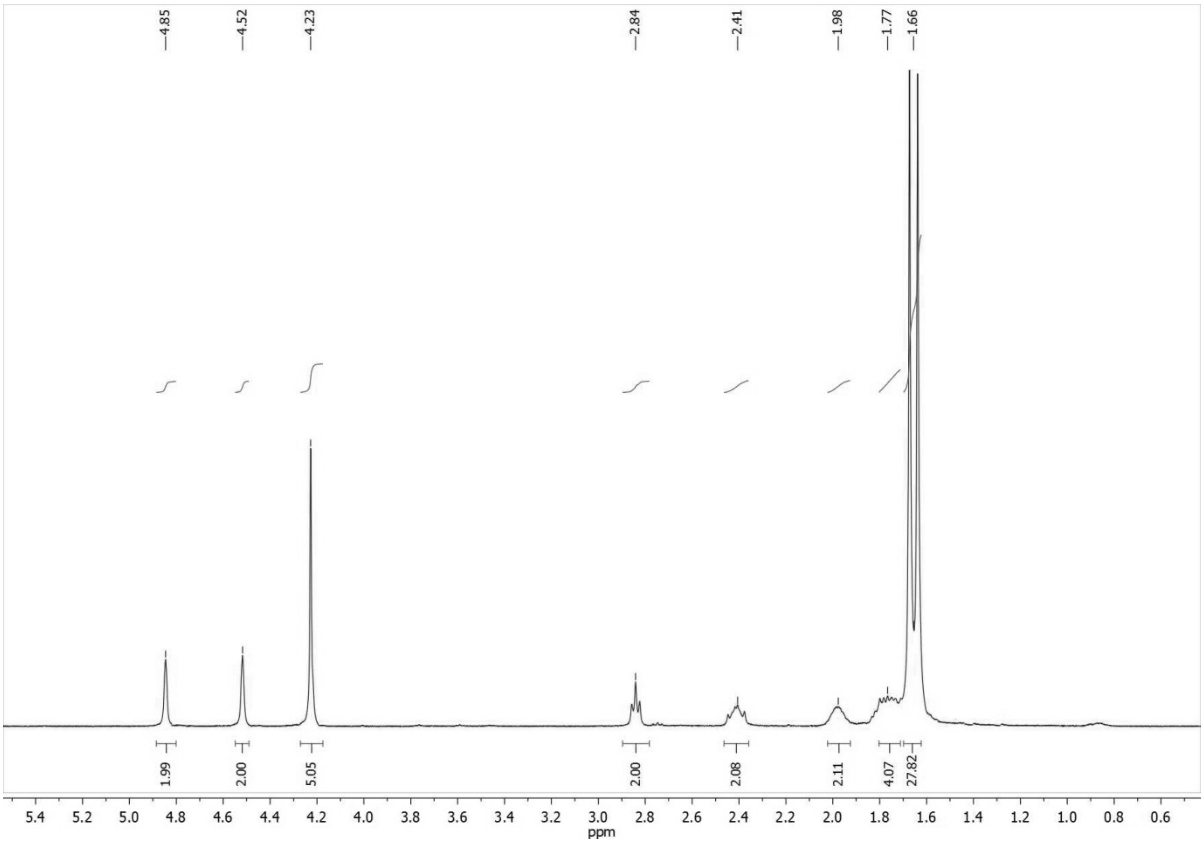

Figure S22. ^{1}H NMR spectra of the tri-*tert*-butyl(6-ferrocenyl-6-oxohexyl)phosphonium tetrafluoroborate **4c**

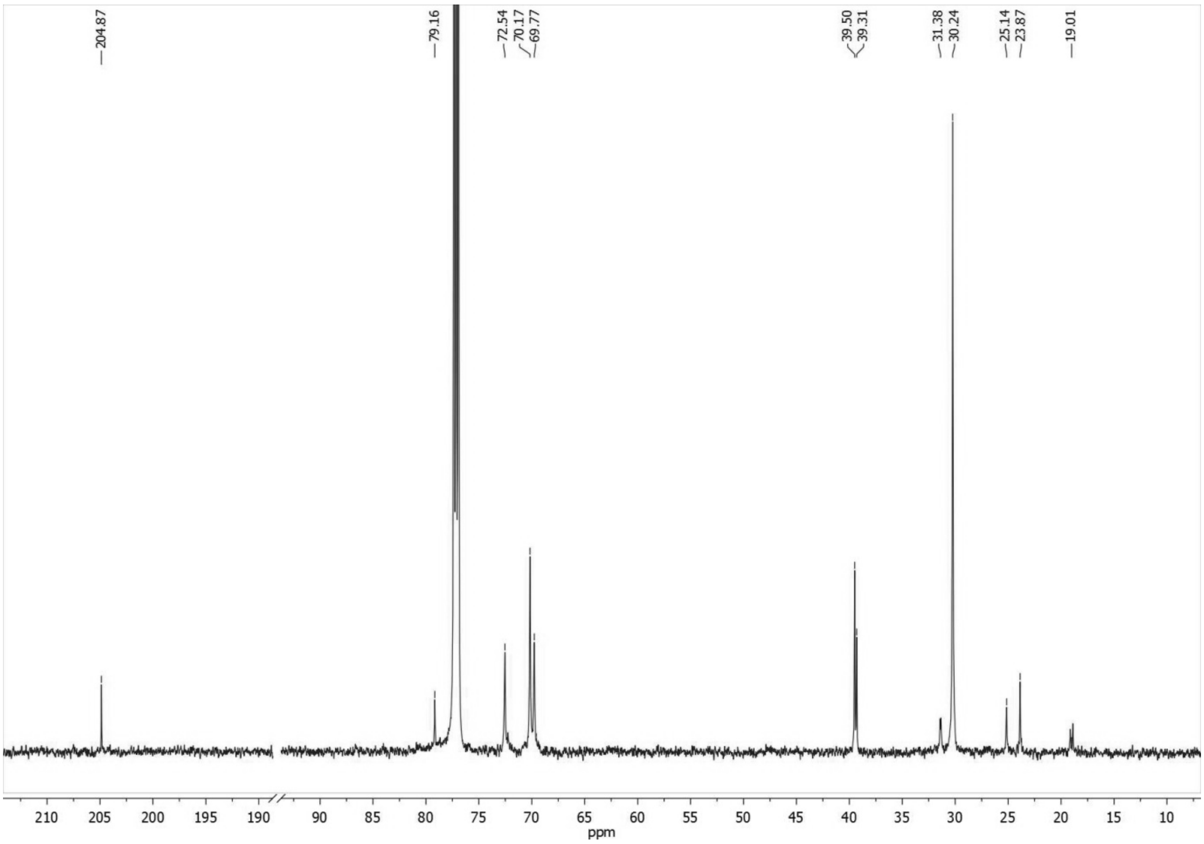

Figure S23. $^{13}C\{^{1}H\}$ NMR spectra of the tri-*tert*-butyl(6-ferrocenyl-6-oxohexyl)phosphonium tetrafluoroborate **4c**

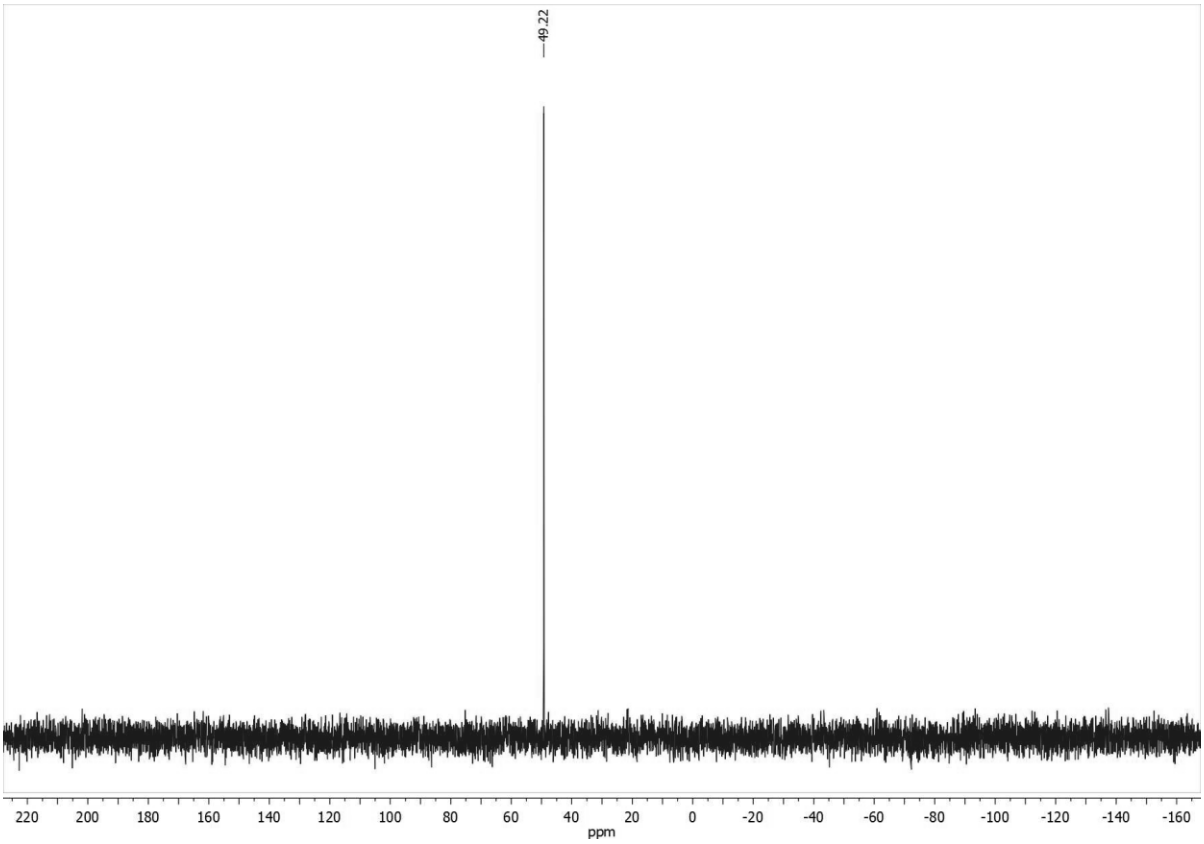

Figure S24. ^{31}P NMR spectra of the tri-*tert*-butyl(11-ferrocenyl-11-oxoundecyl)phosphonium tetrafluoroborate **4d**

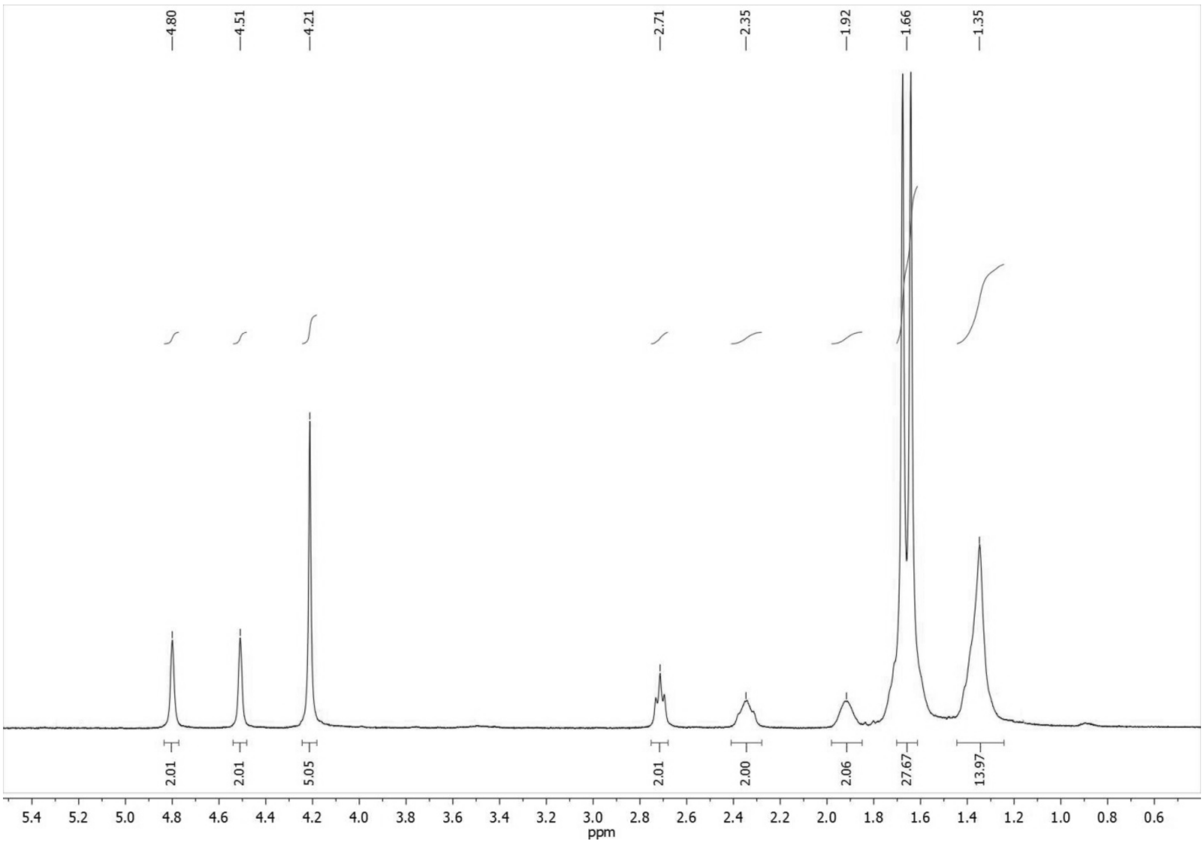

Figure S25.^{1}H NMR spectra of the tri-*tert*-butyl(11-ferrocenyl-11-oxoundecyl)phosphonium tetrafluoroborate **4d**

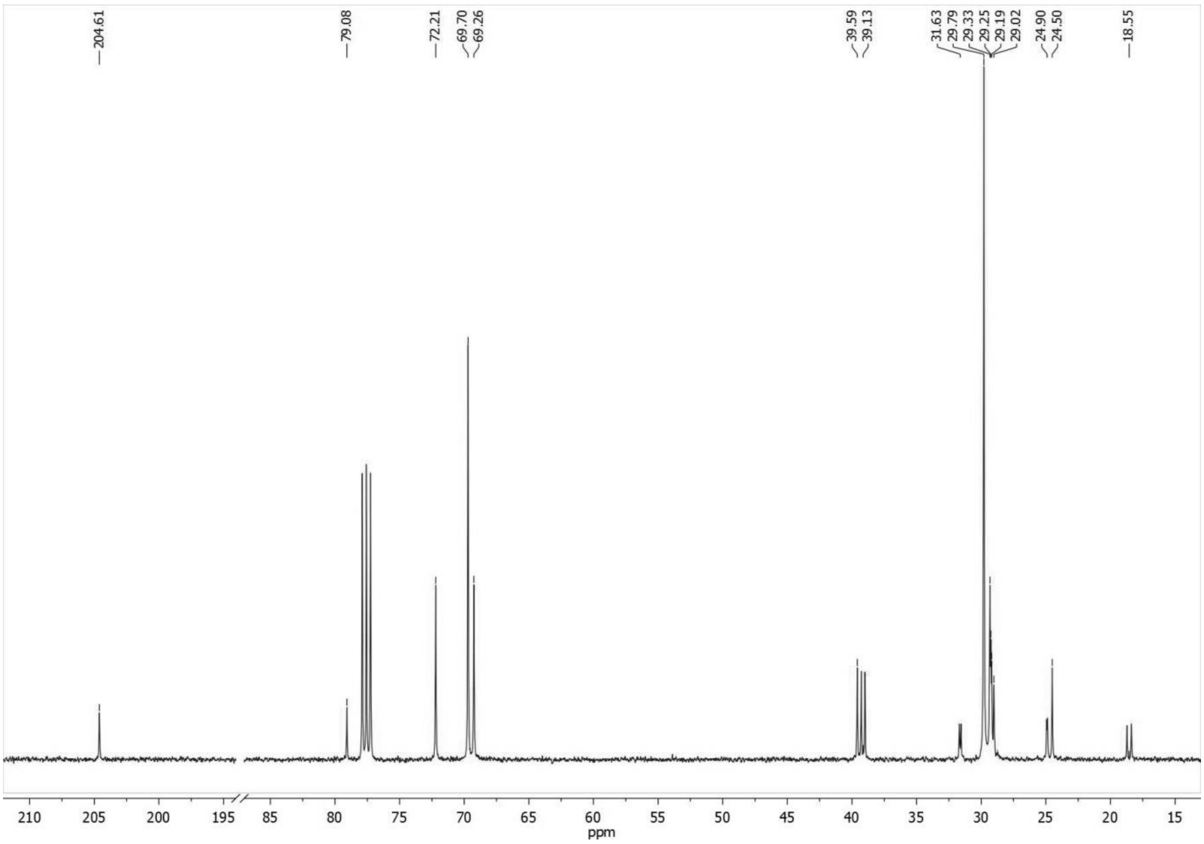

Figure S26. ^{13}C{^{1}H} NMR spectra of the tri-*tert*-butyl(11-ferrocenyl-11-oxoundecyl)phosphonium tetrafluoroborate **4d**

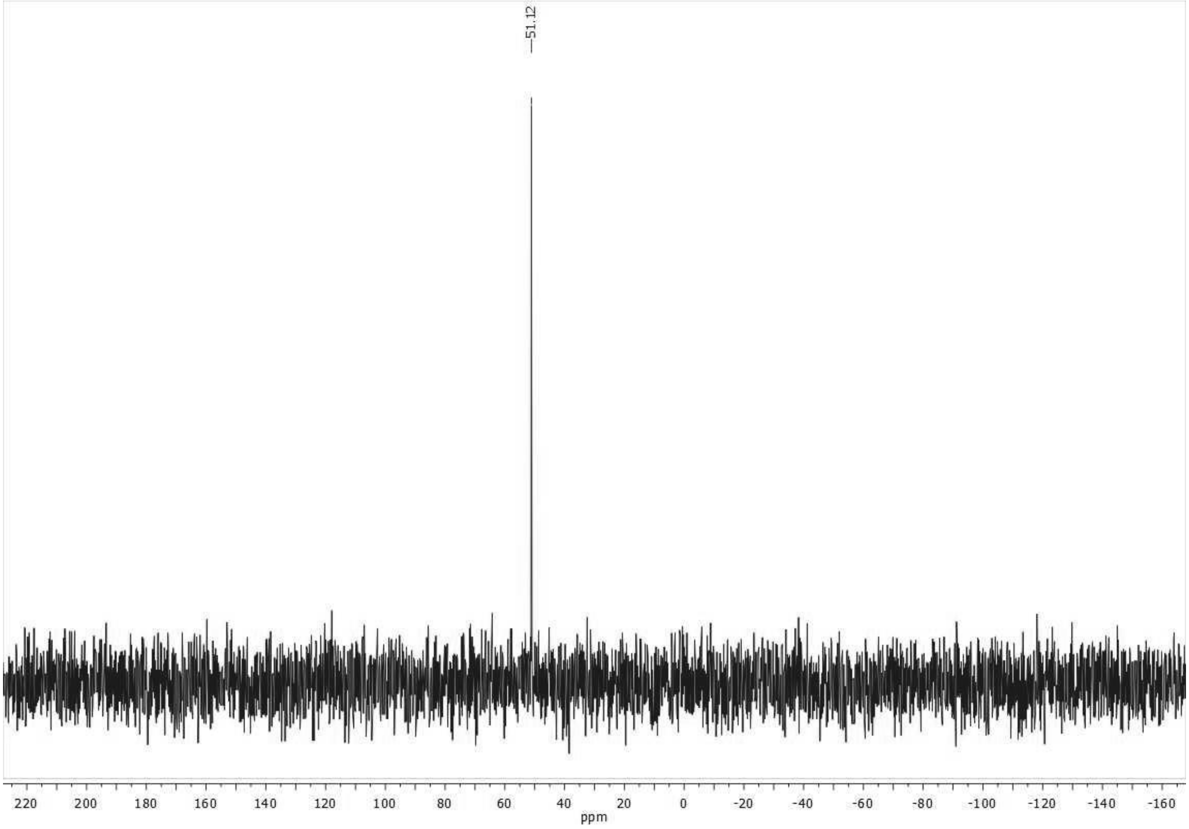

Figure S27. ^{31}P NMR spectra of the tri-*tert*-butyl(3-ferrocenyl-3-oxopropyl)phosphonium bis(trifluoromethanesulfonyl)imide **5a**

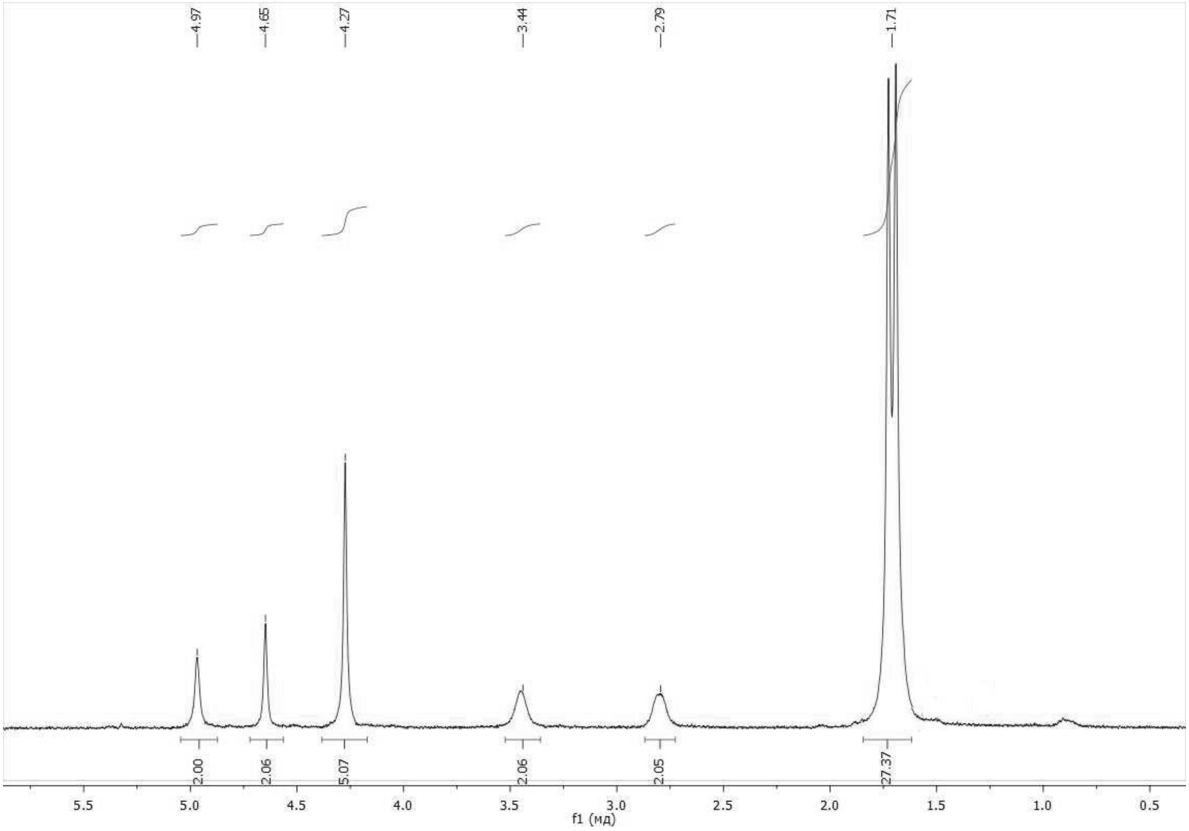

Figure S28. ^{1}H NMR spectra of the tri-*tert*-butyl(3-ferrocenyl-3-oxopropyl)phosphonium bis(trifluoromethanesulfonyl)imide **5a**

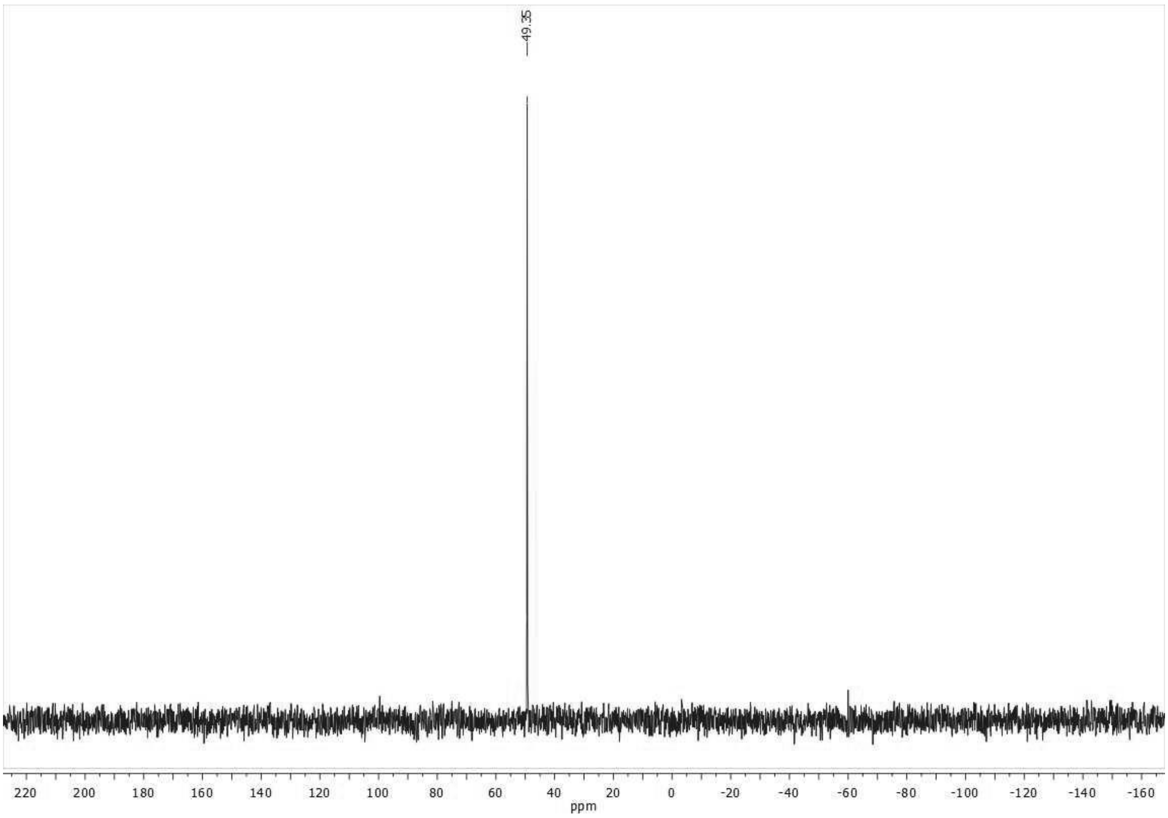

Figure S29. ^{31}P NMR spectra of the tri-*tert*-butyl(5-ferrocenyl-5-oxopentyl)phosphonium bis(trifluoromethanesulfonyl)imide **5b**

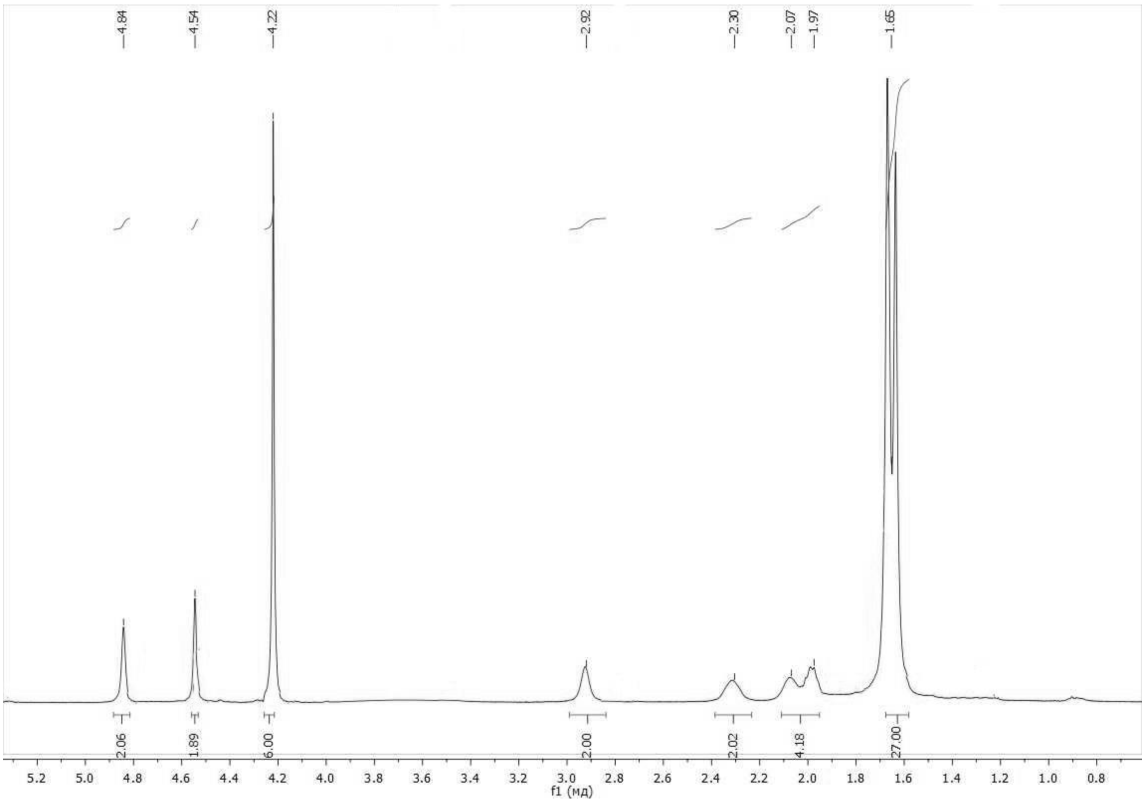

Figure S30. ^{1}H NMR spectra of the tri-*tert*-butyl(5-ferrocenyl-5-oxopentyl)phosphonium bis(trifluoromethanesulfonyl)imide **5b**

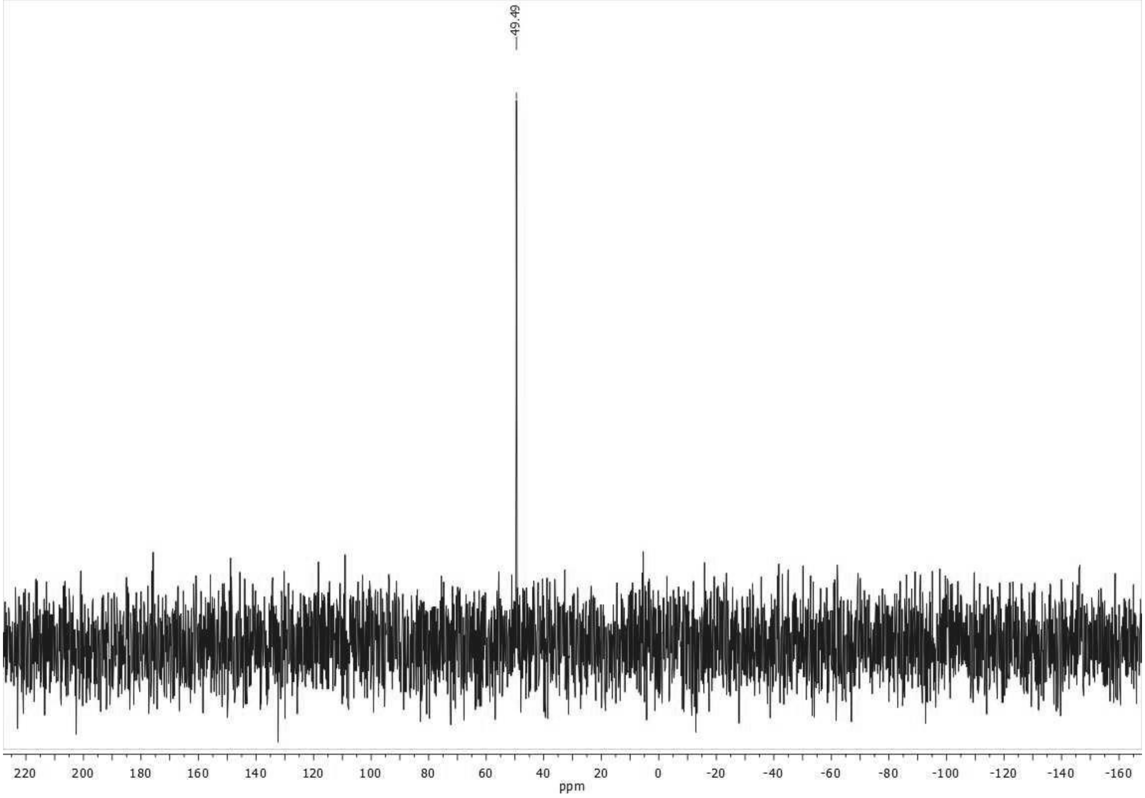

Figure S31. ^{31}P NMR spectra of the tri-*tert*-butyl(6-ferrocenyl-6-oxohexyl)phosphonium bis(trifluoromethanesulfonyl)imide **5c**

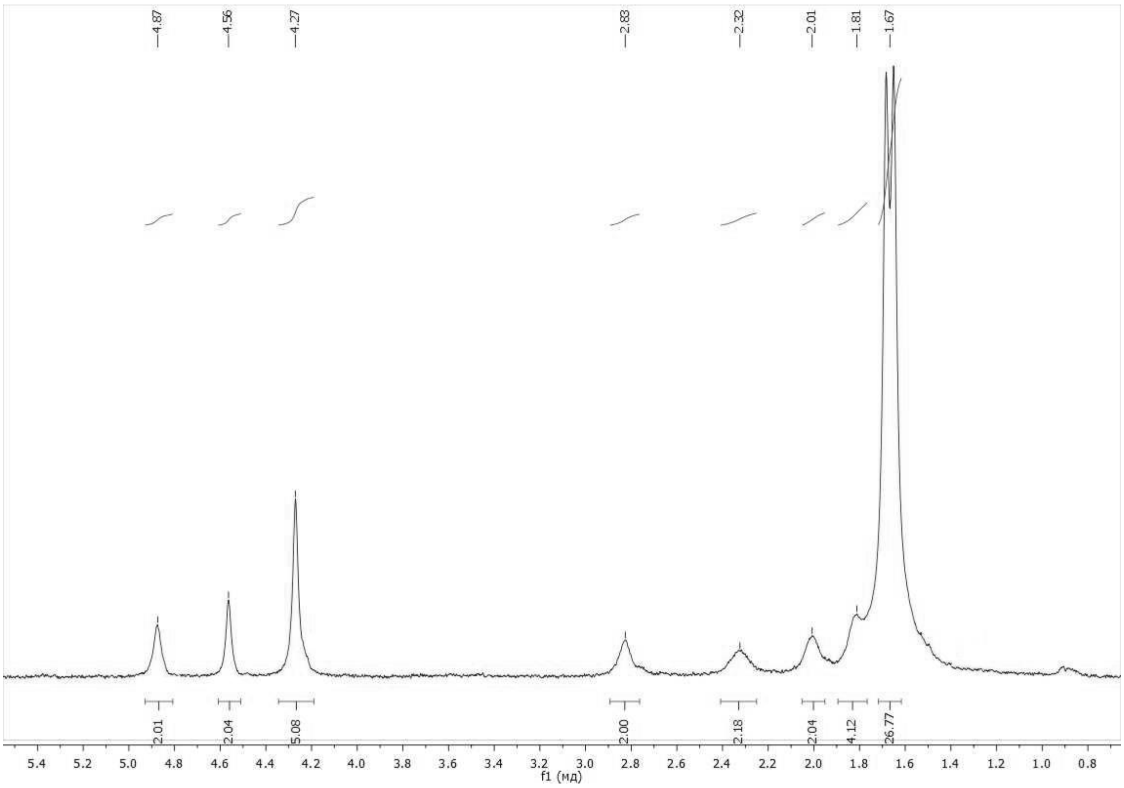

Figure S32. ^{1}H NMR spectra of the tri-*tert*-butyl(6-ferrocenyl-6-oxohexyl)phosphonium bis(trifluoromethanesulfonyl)imide **5c**

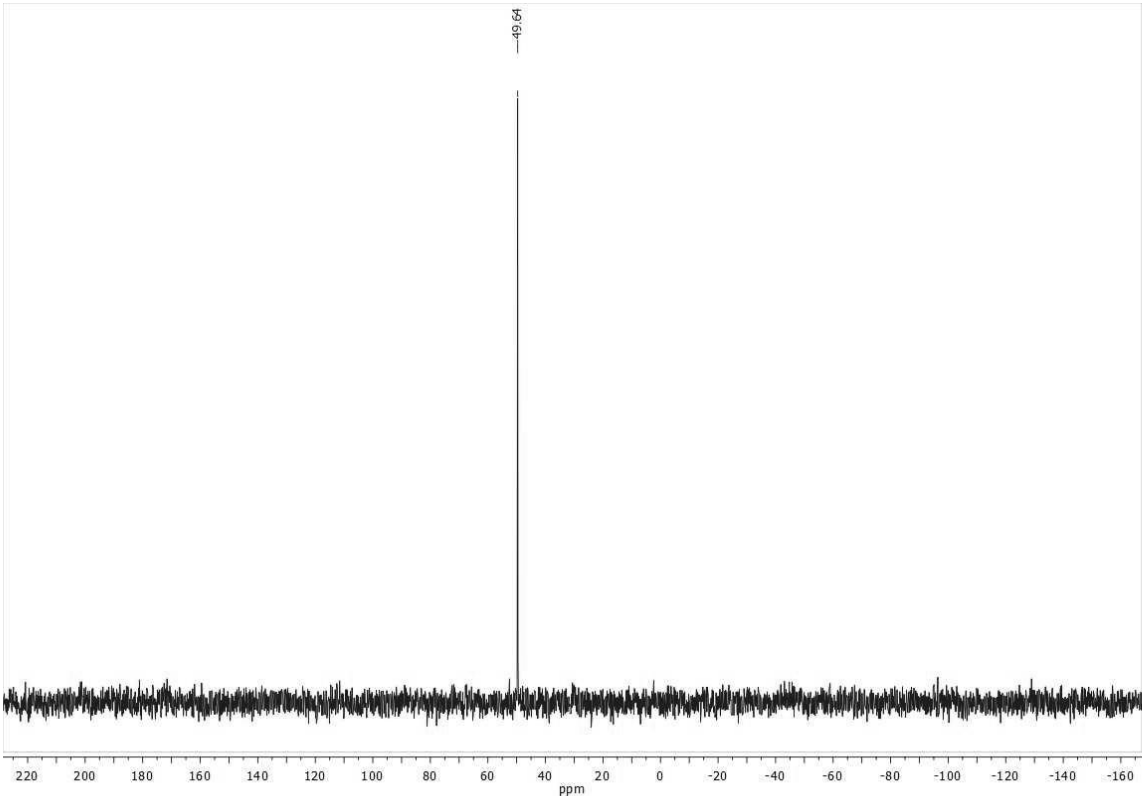

Figure S33. ^{31}P NMR spectra of the tri-*tert*-butyl(11-ferrocenyl-11-oxoundecyl)phosphonium bis(trifluoromethanesulfonyl)imide **5d**

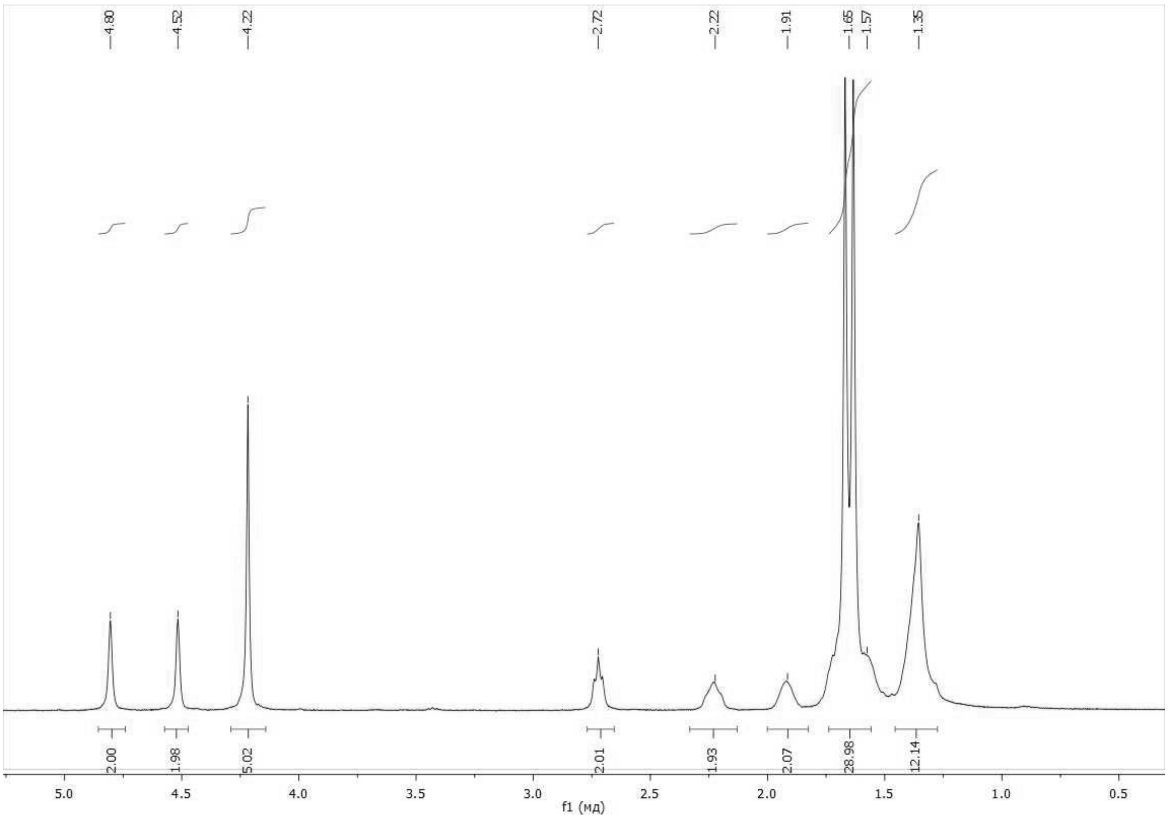

Figure S34. ^{1}H NMR spectra of the tri-*tert*-butyl(11-ferrocenyl-11-oxoundecyl)phosphonium bis(trifluoromethanesulfonyl)imide **5d**

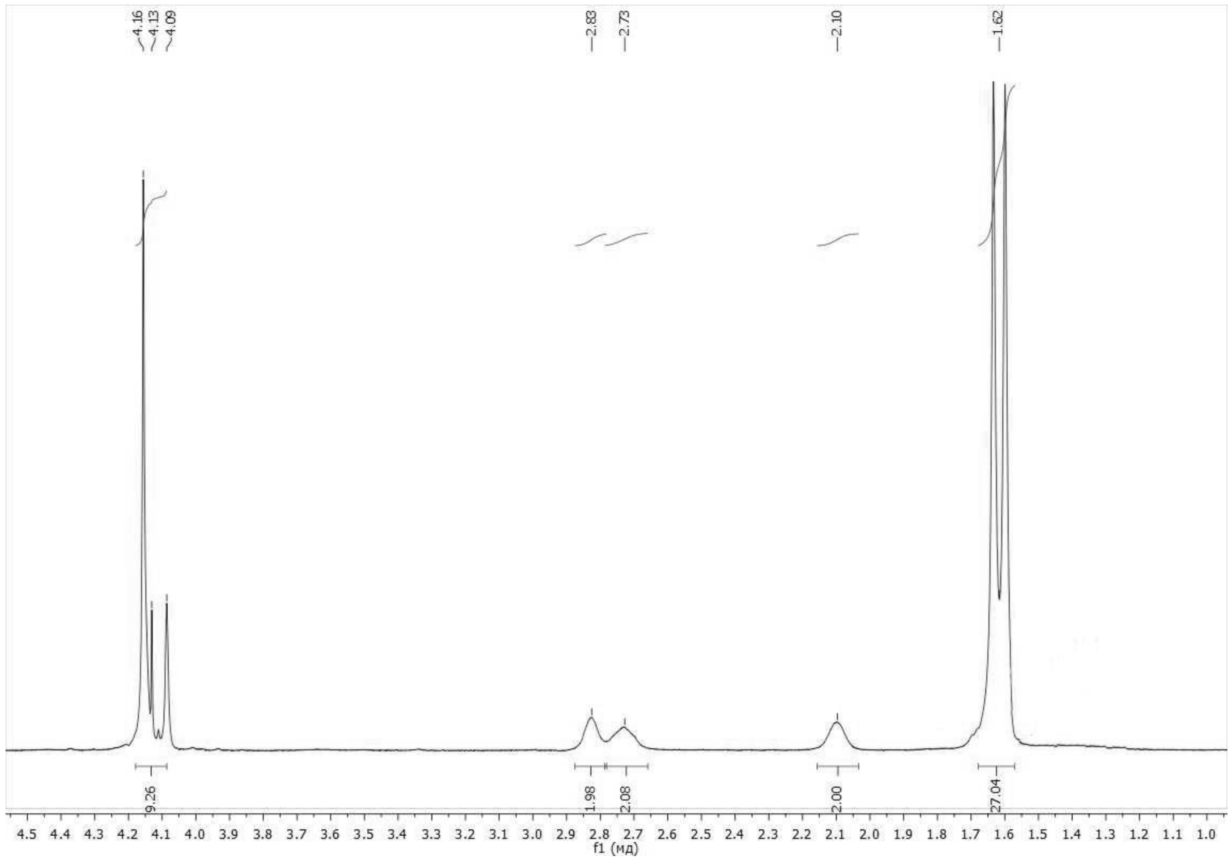

Figure S35. ^{1}H NMR spectra of the tri-*tert*-butyl(3-ferrocenylpropyl)phosphonium bromide **7a**

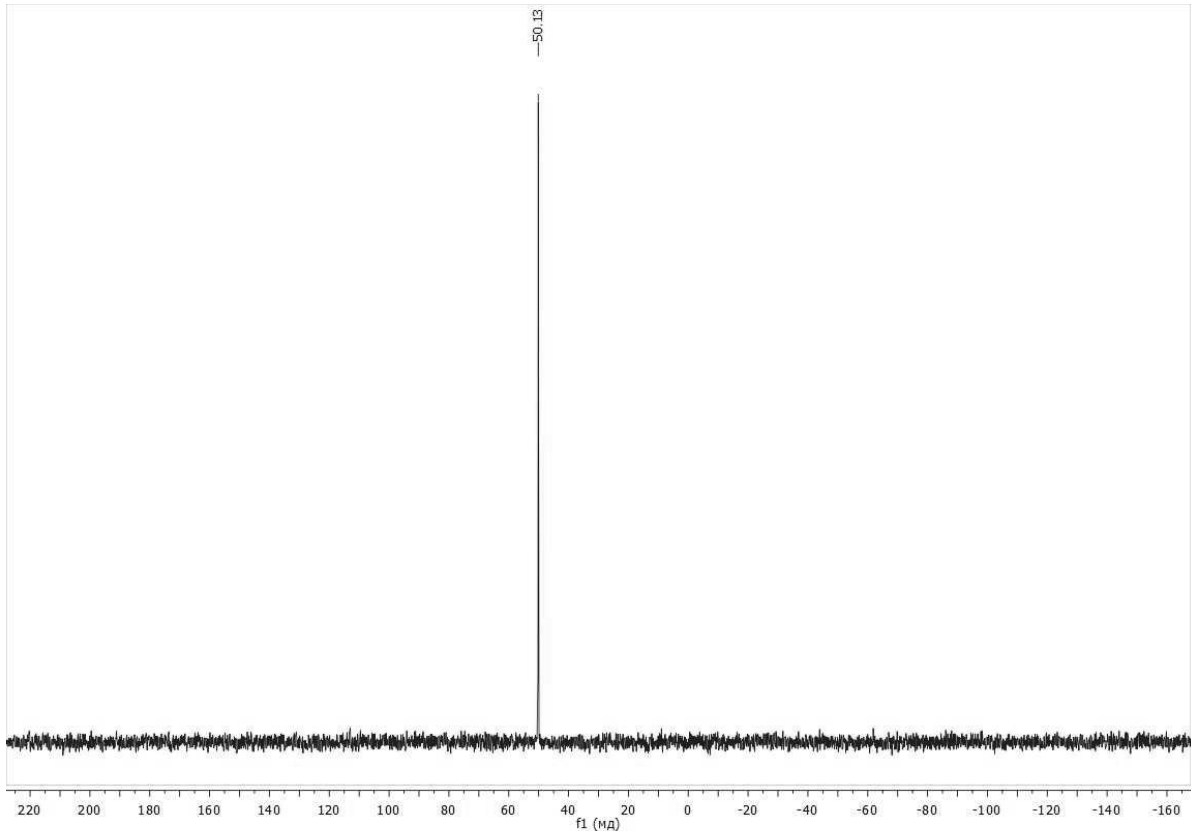

Figure S36. ^{31}P NMR spectra of the tri-*tert*-butyl(3-ferrocenylpropyl)phosphonium bromide **7a**

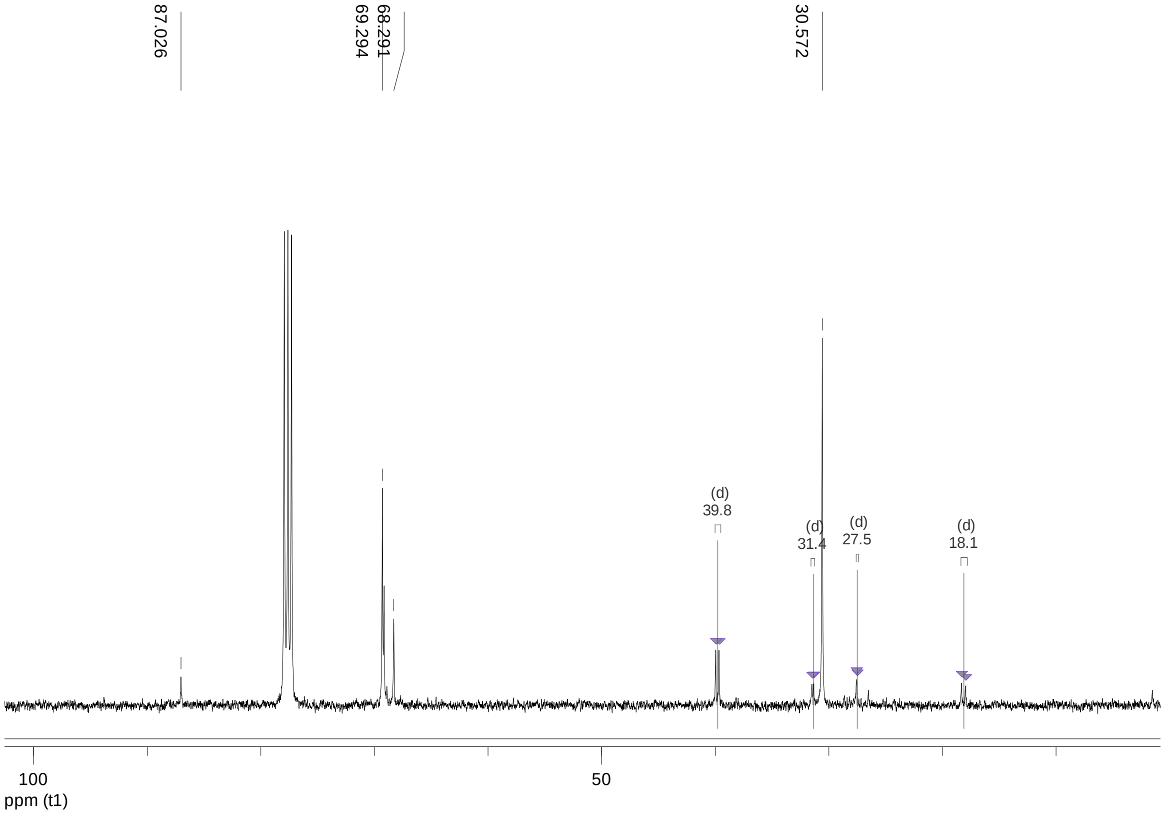

Figure S37. $^{13}C\{^{1}H\}$ NMR spectra of the tri-*tert*-butyl(3-ferrocenylpropyl)phosphonium bromide **7a**

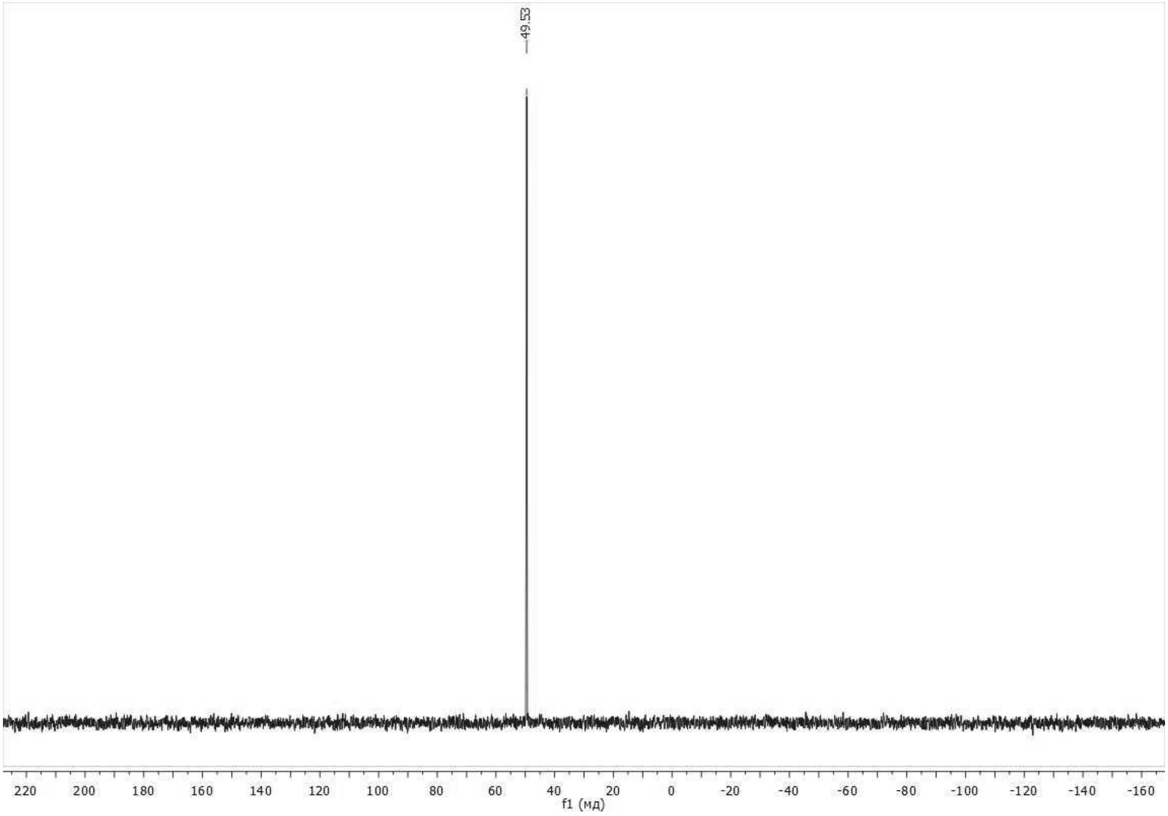

Figure S38. ^{31}P NMR spectra of the tri-*tert*-butyl(5-ferrocenylpentyl)phosphonium bromide **7b**

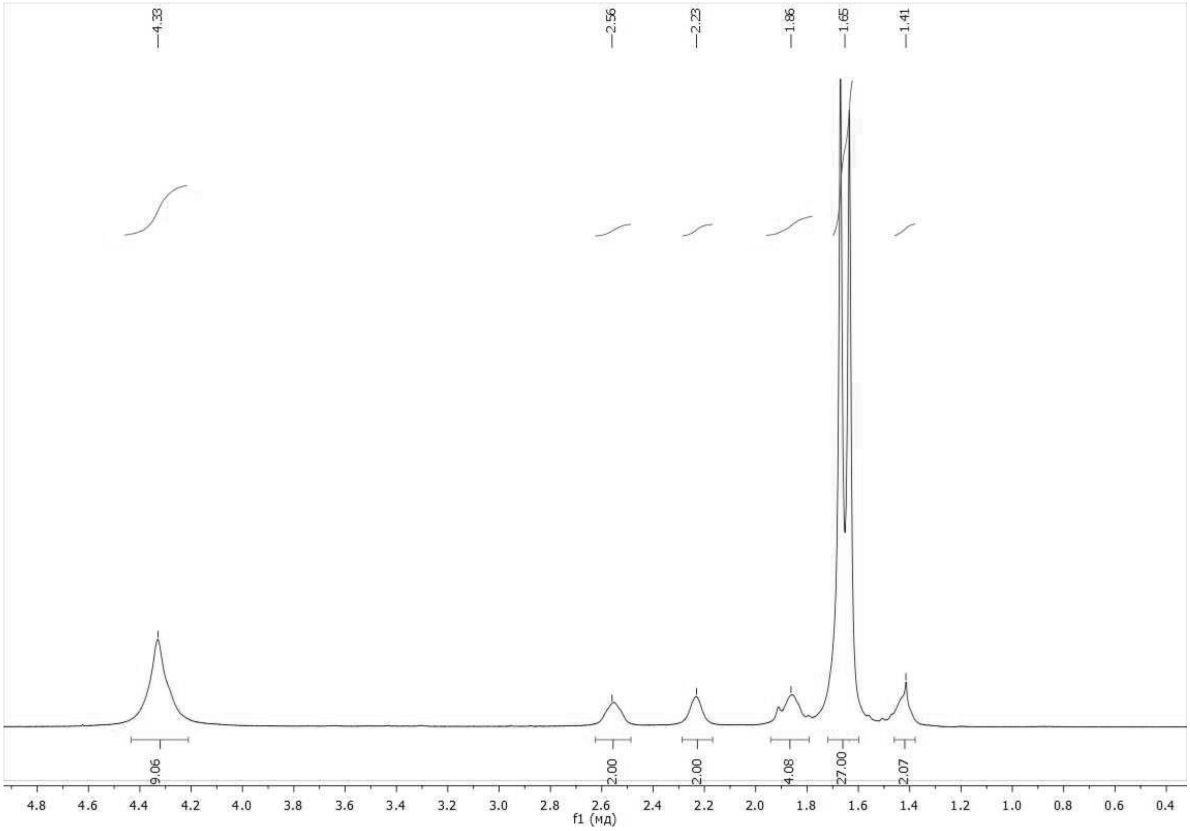

Figure S39. ^{1}H NMR spectra of the tri-*tert*-butyl(5-ferrocenylpentyl)phosphonium bromide **7b**

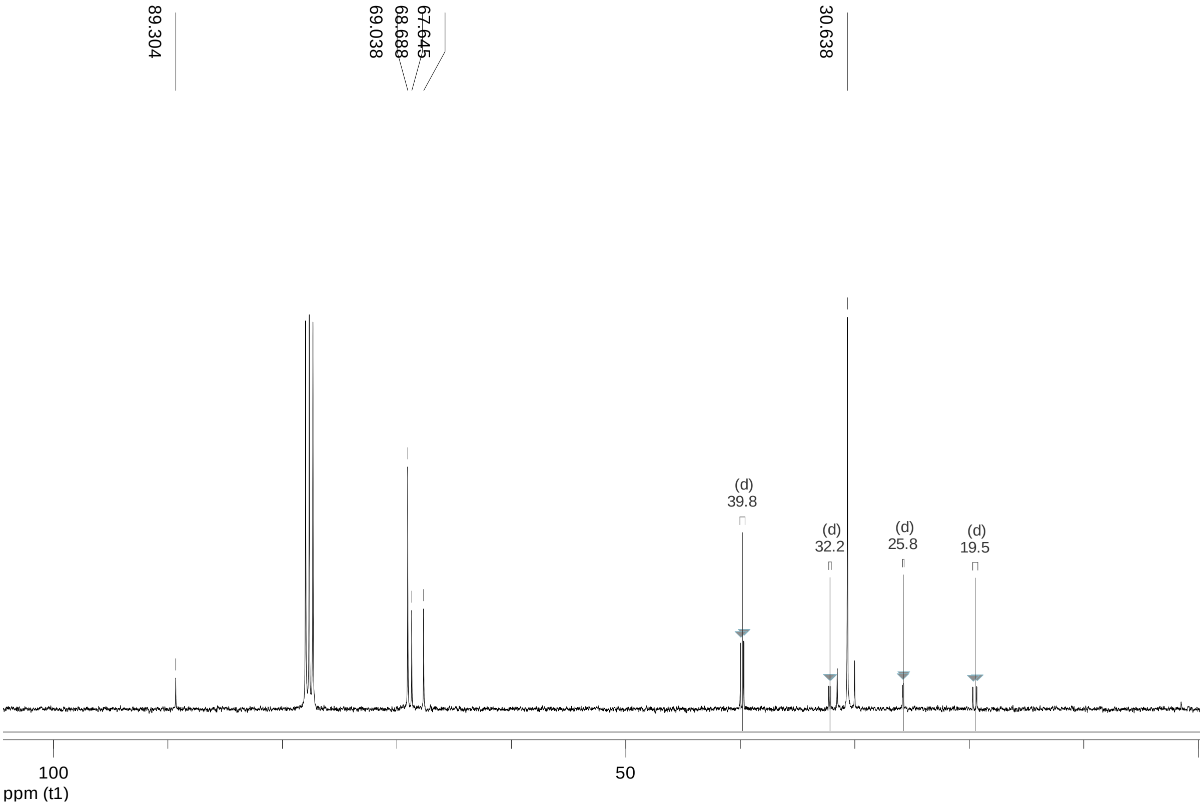

Figure S40. $^{13}C\{^{1}H\}$ NMR spectra of the tri-*tert*-butyl(5-ferrocenylpentyl)phosphonium bromide **7b**

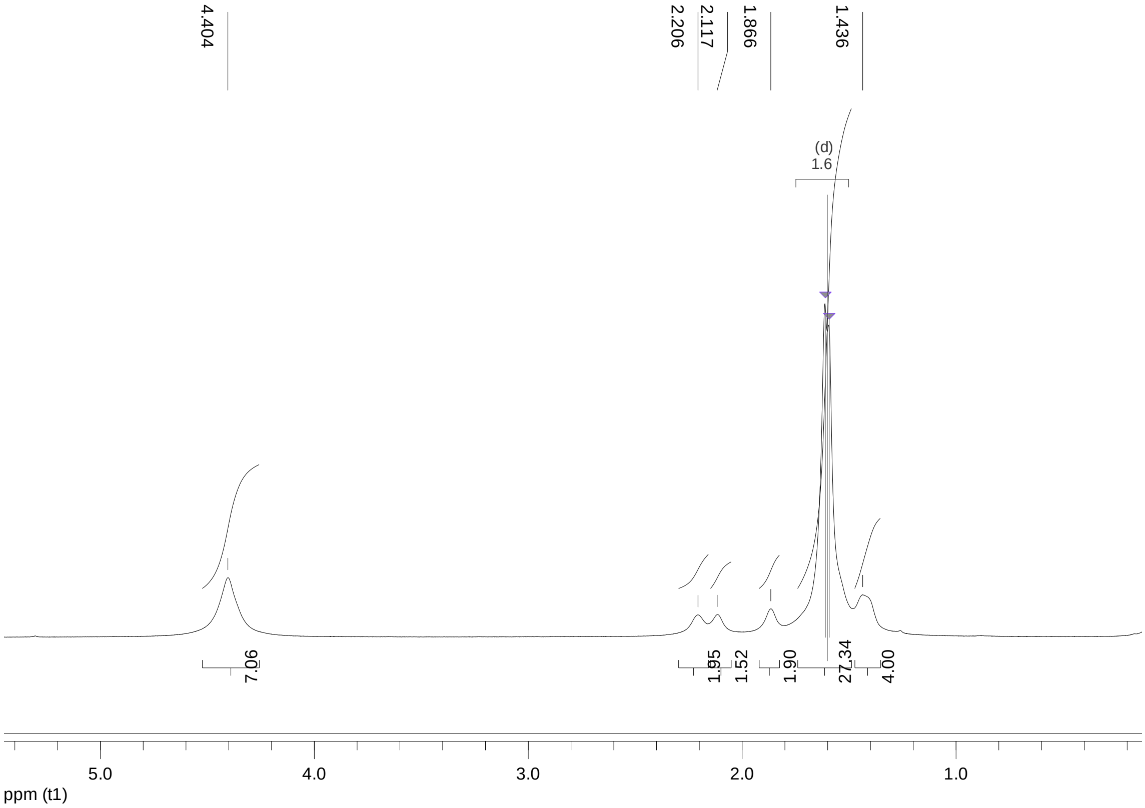

Figure S41.^{1}H NMR spectra of the tri-*tert*-butyl(6-ferrocenylhexyl)phosphonium bromide **7c**

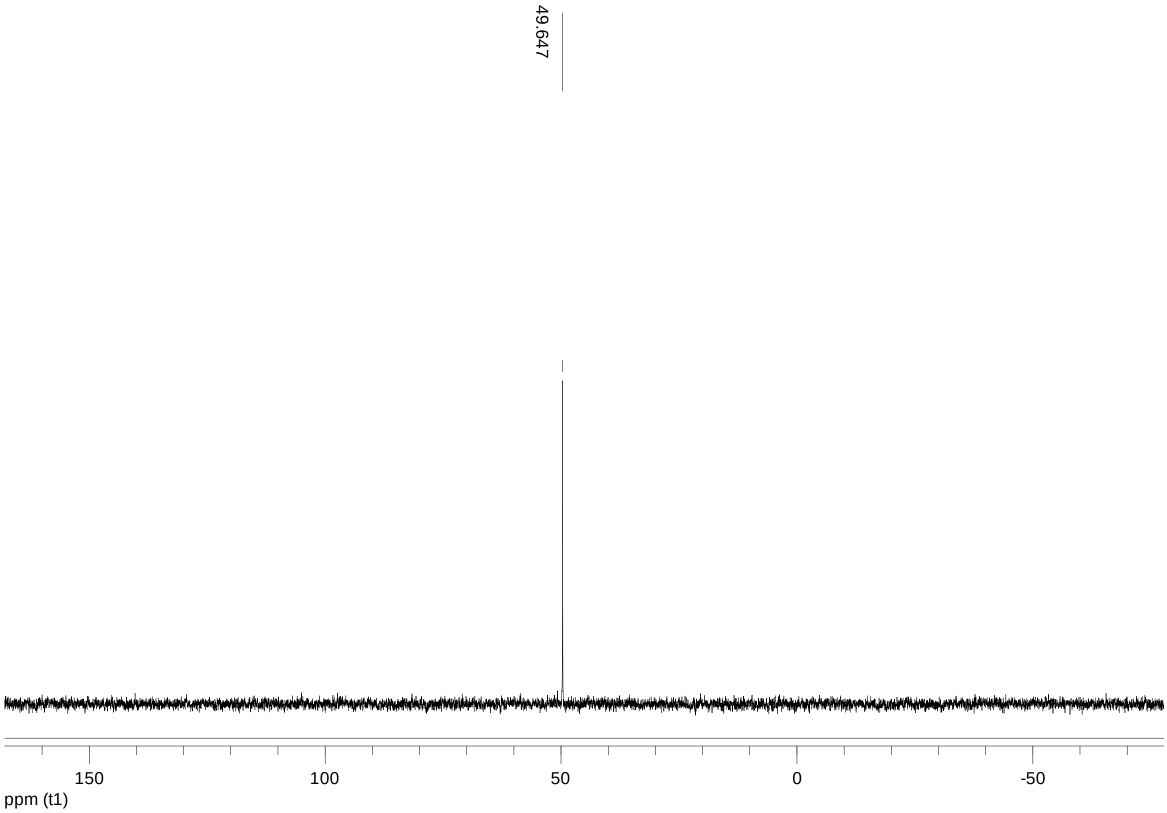

Figure S42.^{31}P NMR spectra of the tri-*tert*-butyl(6-ferrocenylhexyl)phosphonium bromide **7c**

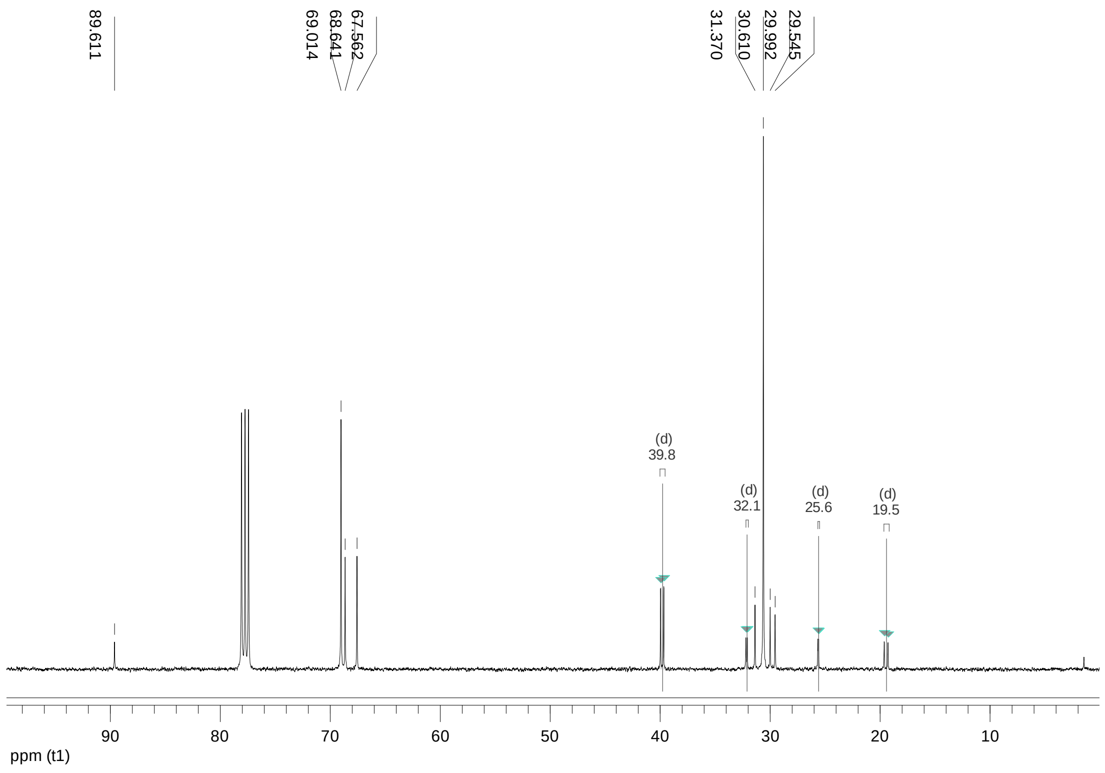

Figure S43. $^{13}C\{^{1}H\}$ NMR spectra of the tri-*tert*-butyl(6-ferrocenylhexyl)phosphonium bromide **7c**

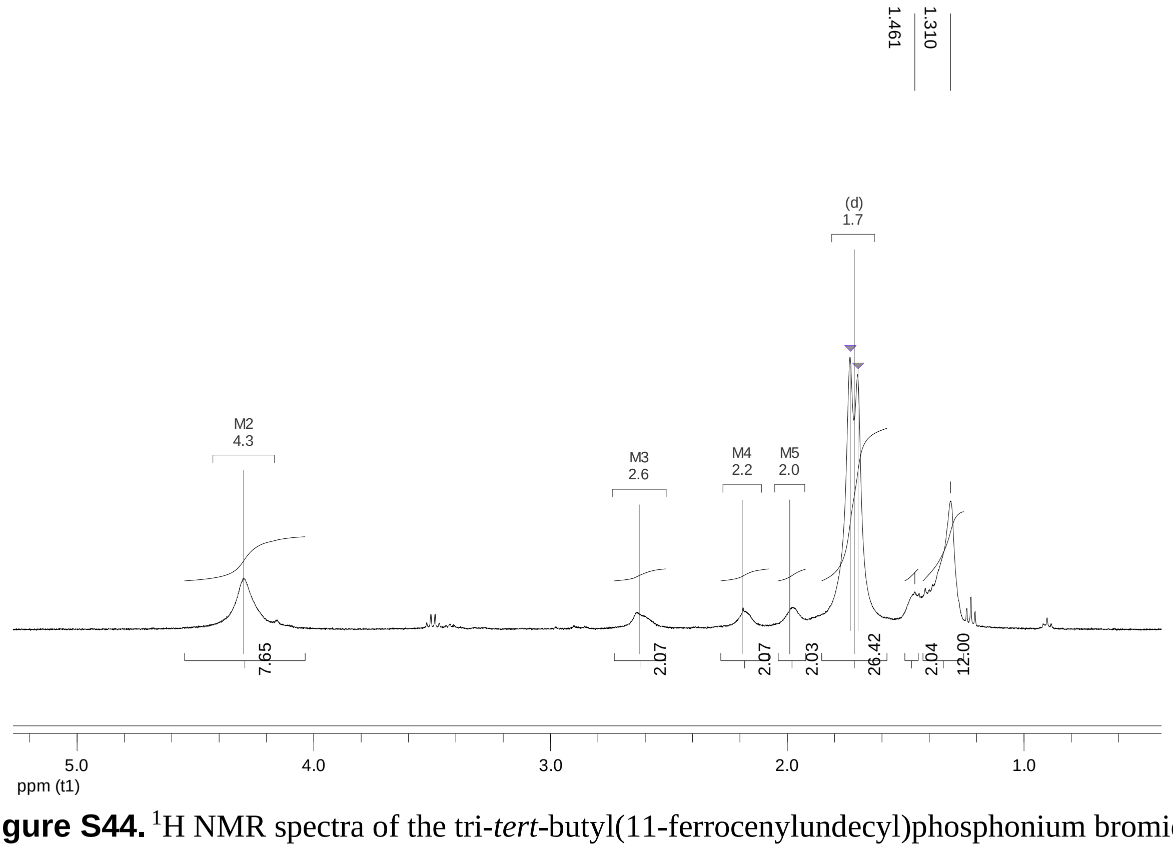

Figure S44. ^{1}H NMR spectra of the tri-*tert*-butyl(11-ferrocenylundecyl)phosphonium bromide **7d**

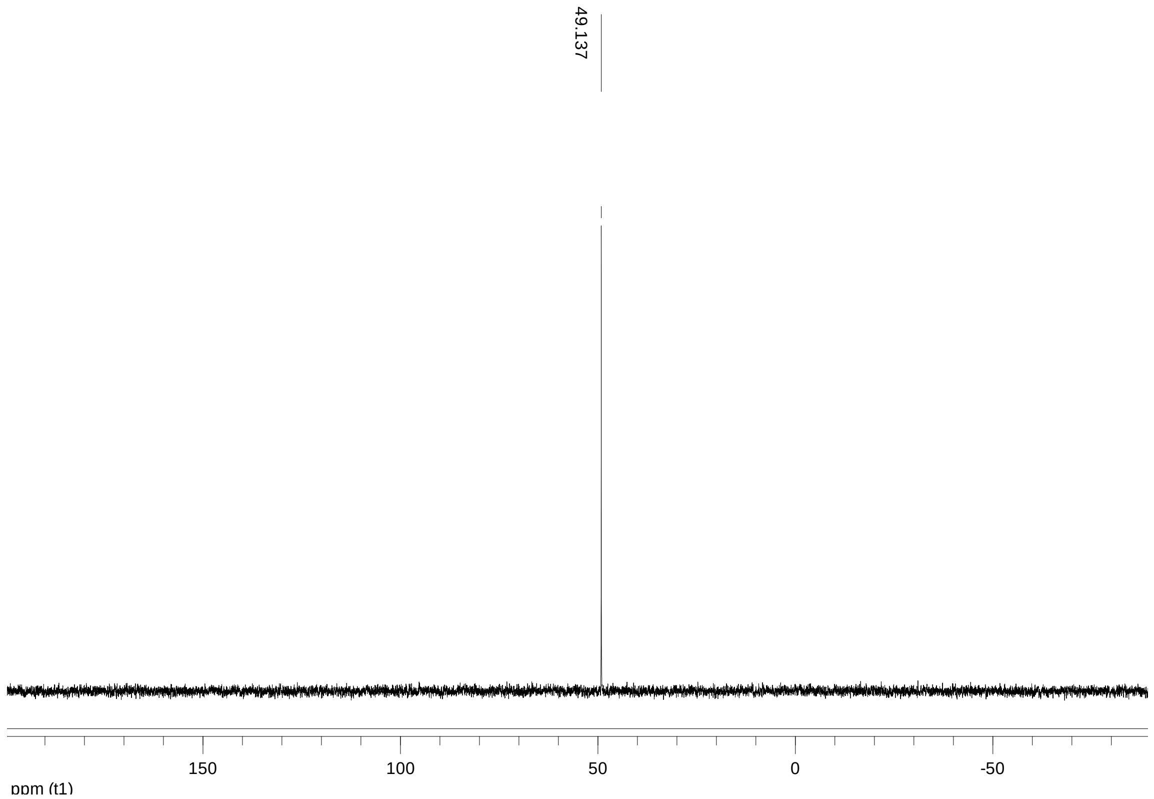

Figure S45. ^{31}P NMR spectra of the tri-*tert*-butyl(11-ferrocenylundecyl)phosphonium bromide **7d**

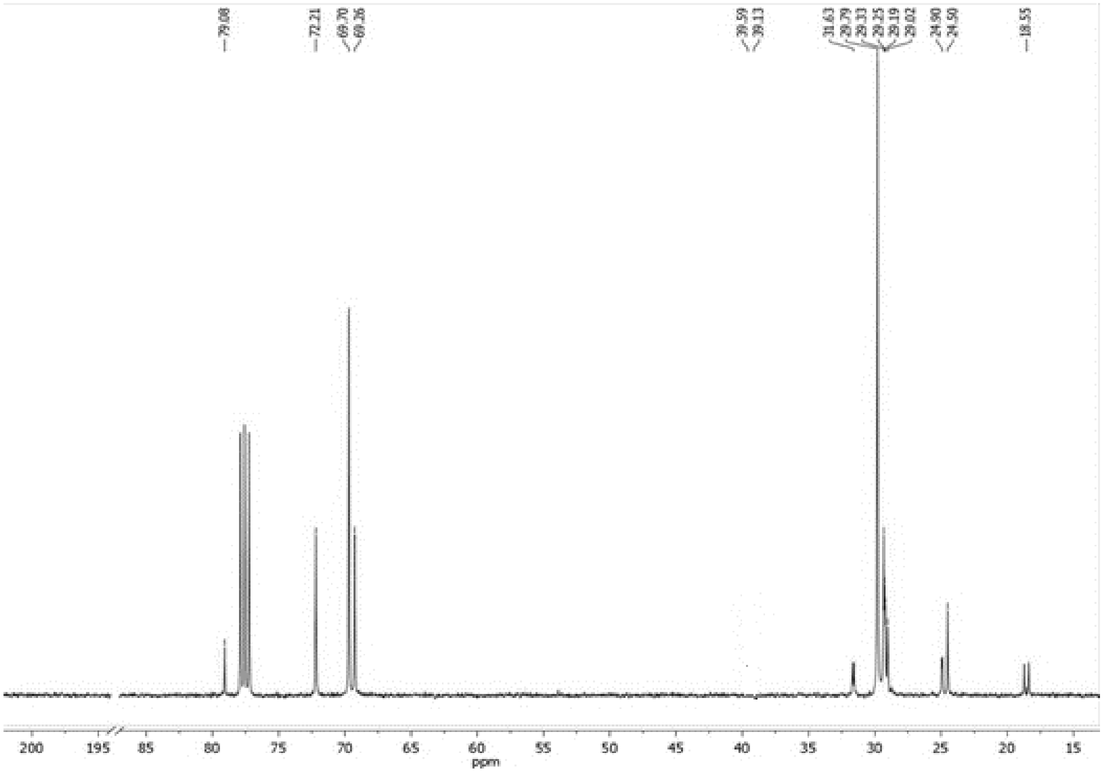

Figure S46. $^{13}C\{^{1}H\}$ NMR spectra of the tri-*tert*-butyl(11-ferrocenylundecyl)phosphonium bromide **7d**

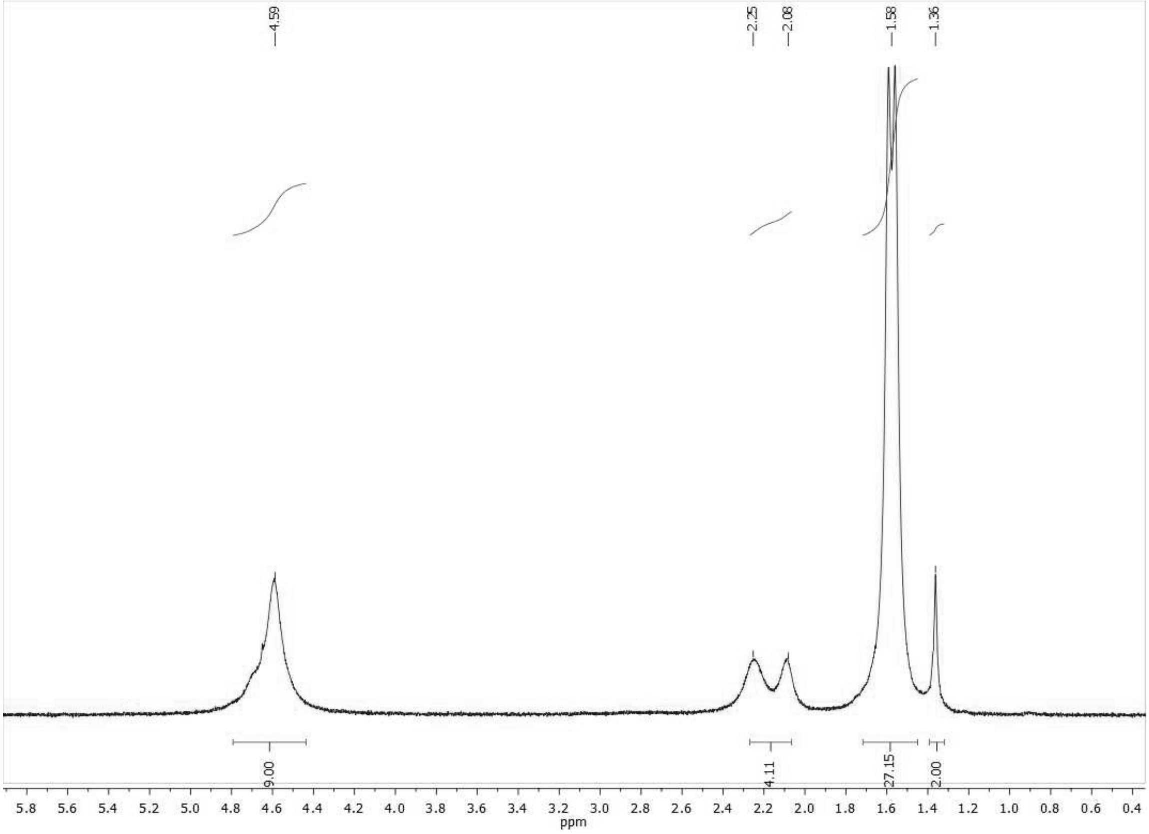

Figure S47. ^{1}H NMR spectra of the tri-*tert*-butyl(3-ferrocenylpropyl)phosphonium tetrafluoroborate **8a**

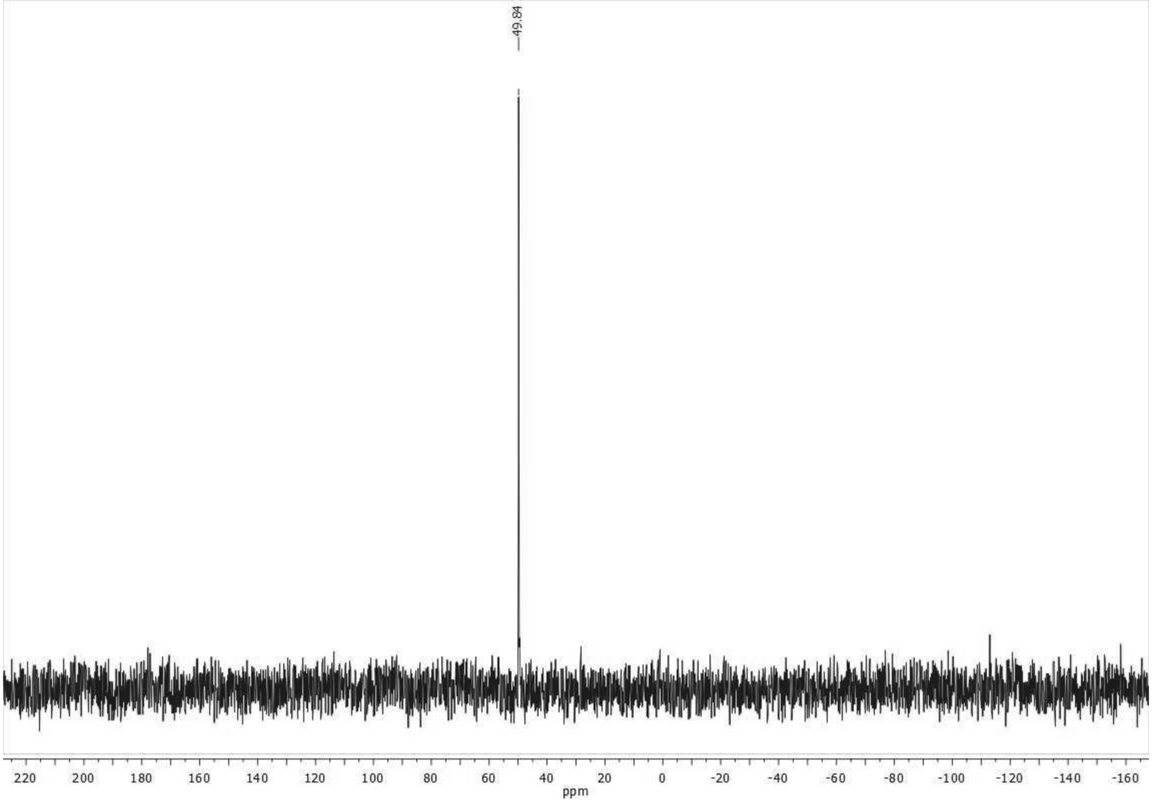

Figure S48. ^{31}P NMR spectra of the tri-*tert*-butyl(3-ferrocenylpropyl)phosphonium tetrafluoroborate **8a**

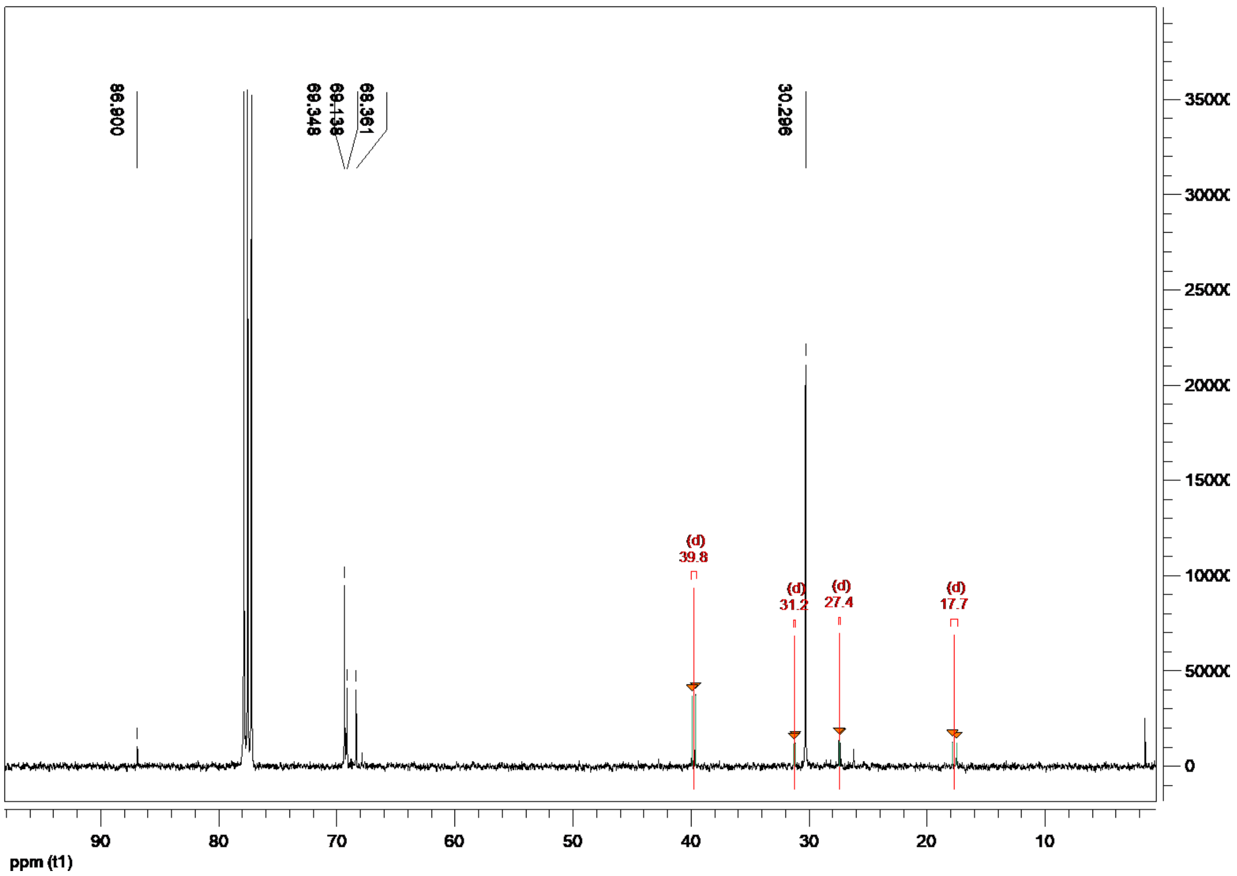

Figure S49.$^{13}C\{^{1}H\}$ NMR spectra of the tri-*tert*-butyl(3-ferrocenylpropyl)phosphonium tetrafluoroborate **8a**

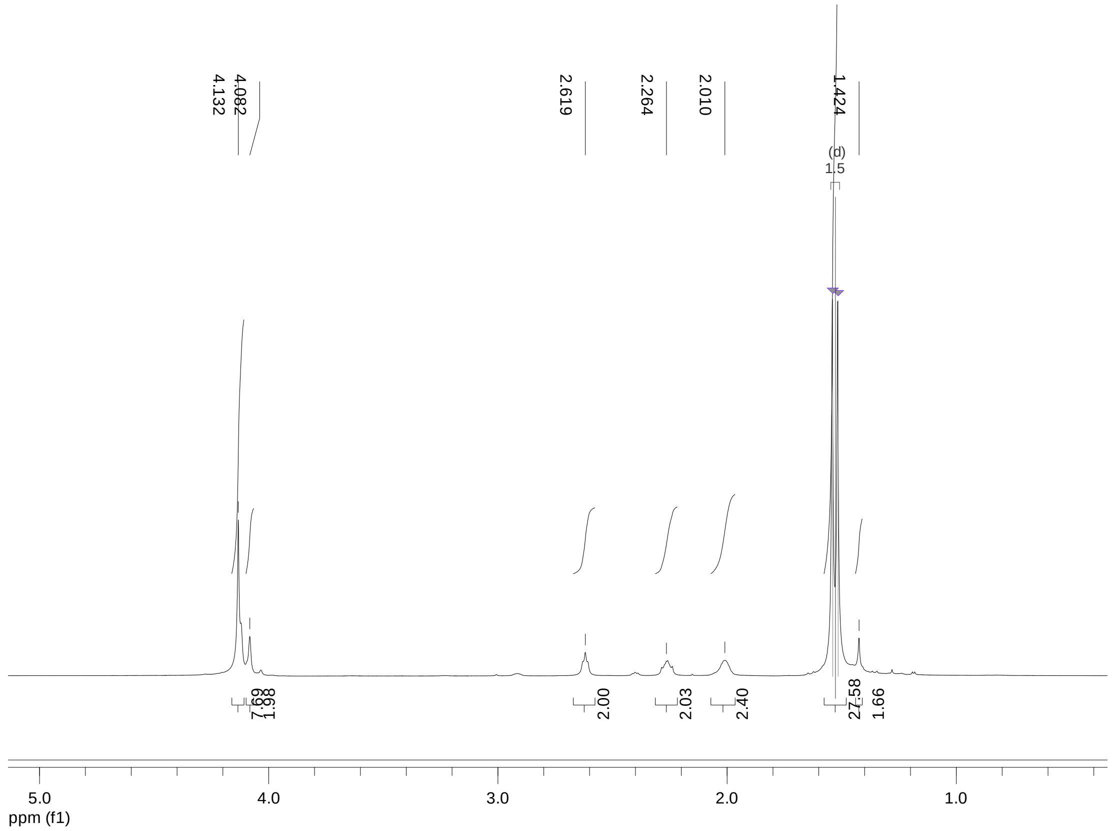

Figure S50.^{1}H NMR spectra of the tri-*tert*-butyl(5-ferrocenylpentyl)phosphonium tetrafluoroborate **8b**

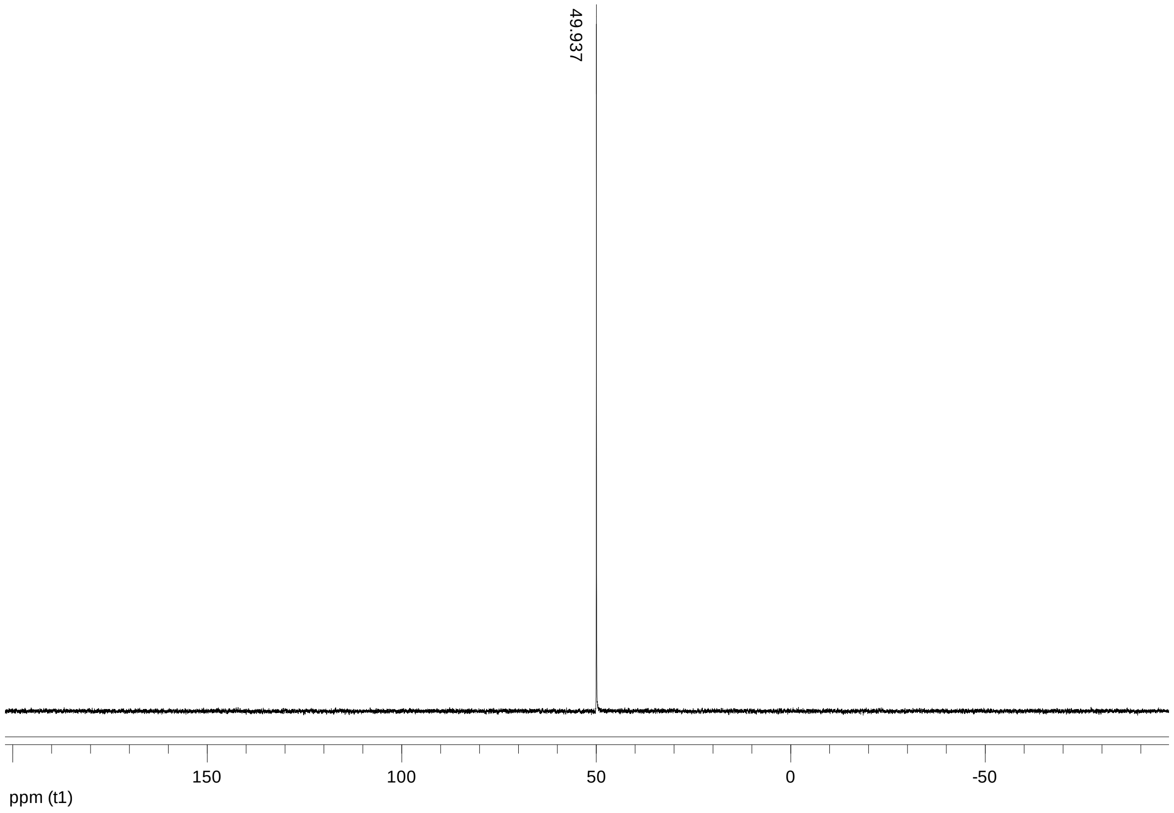

Figure S51. ^{31}P NMR spectra of the tri-*tert*-butyl(5-ferrocenylpentyl) phosphonium tetrafluoroborate **8b**

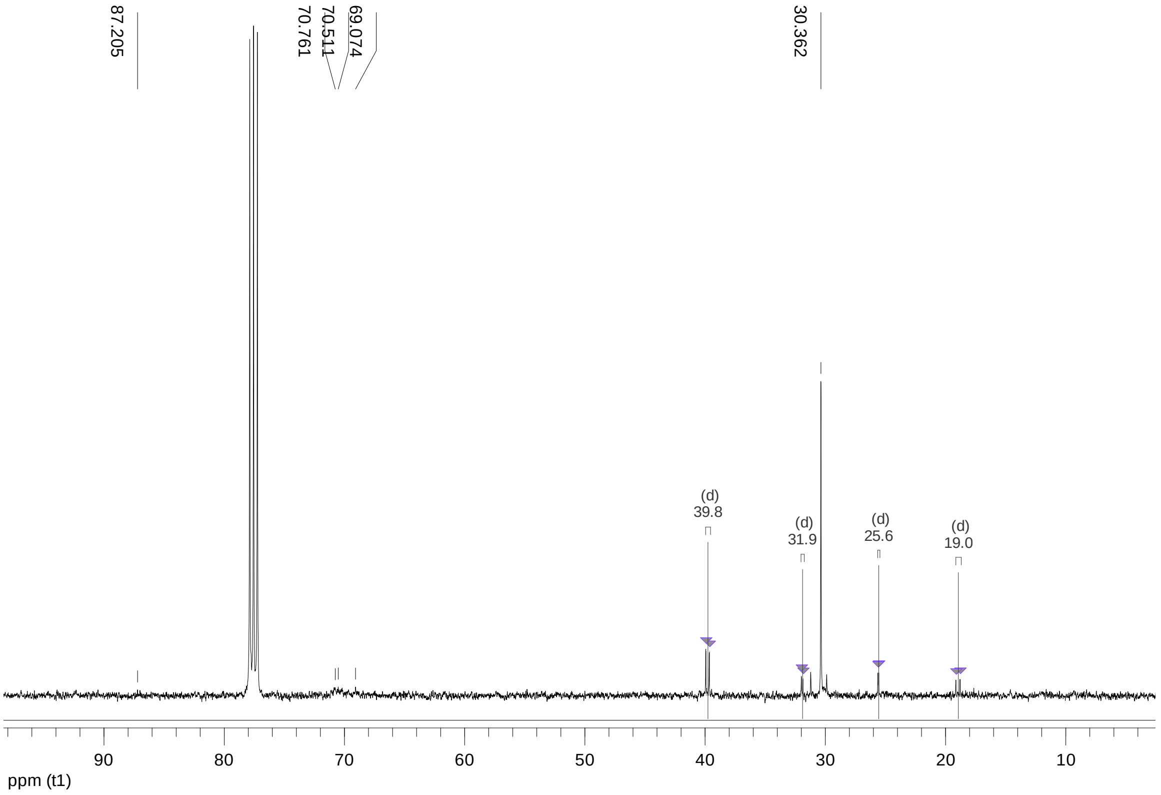

Figure S52. ^{13}C{^{1}H} NMR spectra of the tri-*tert*-butyl(5-ferrocenylpentyl)phosphonium tetrafluoroborate **8b**

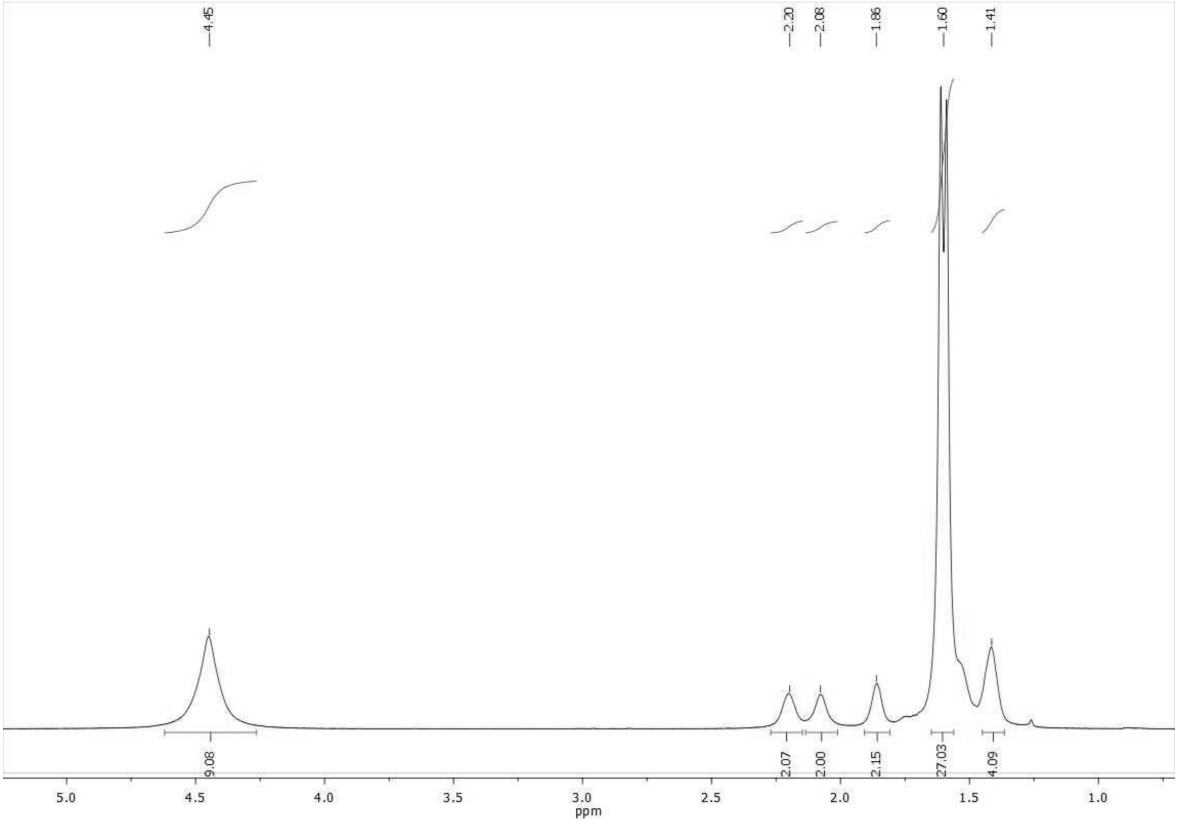

Figure S53. ^{1}H NMR spectra of the tri-*tert*-butyl(6-ferrocenylhexyl)phosphonium tetrafluoroborate **8c**

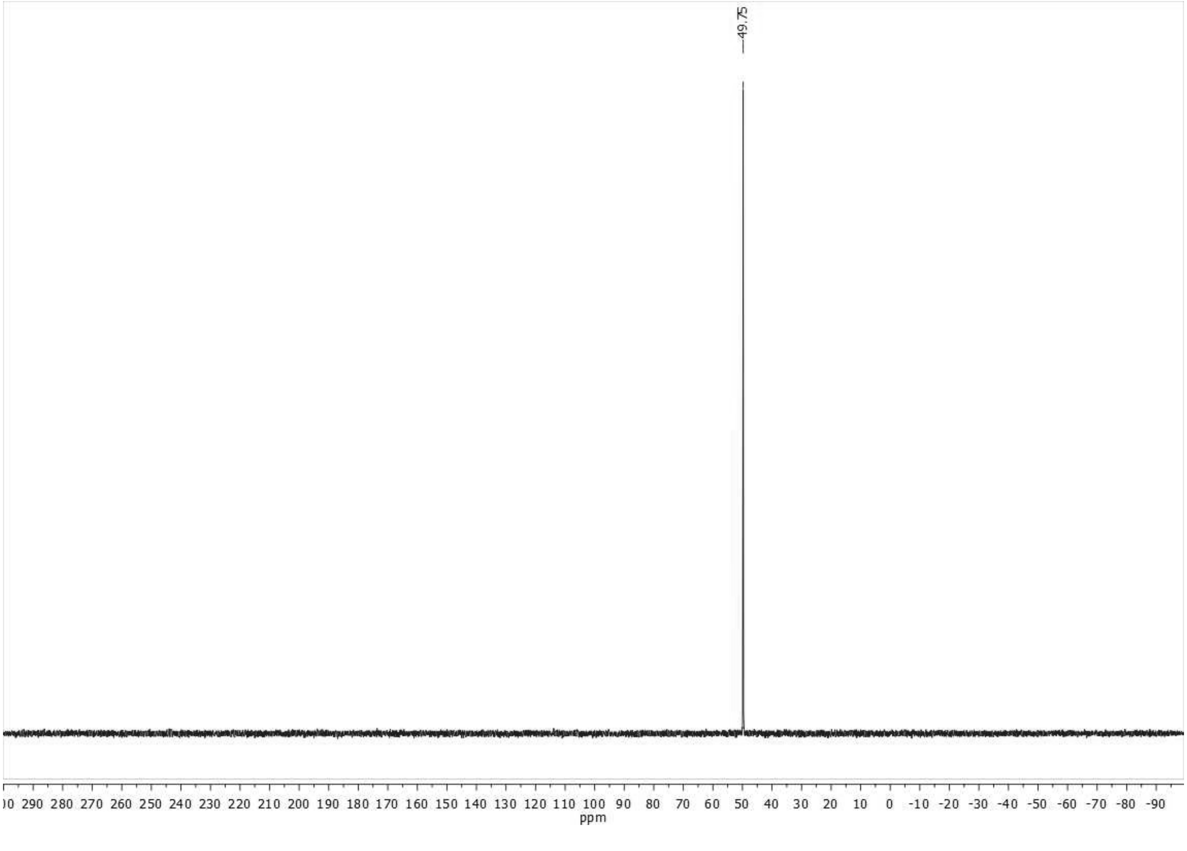

Figure S54. ^{31}P NMR spectra of the tri-*tert*-butyl(6-ferrocenylhexyl)phosphonium tetrafluoroborate **8c**

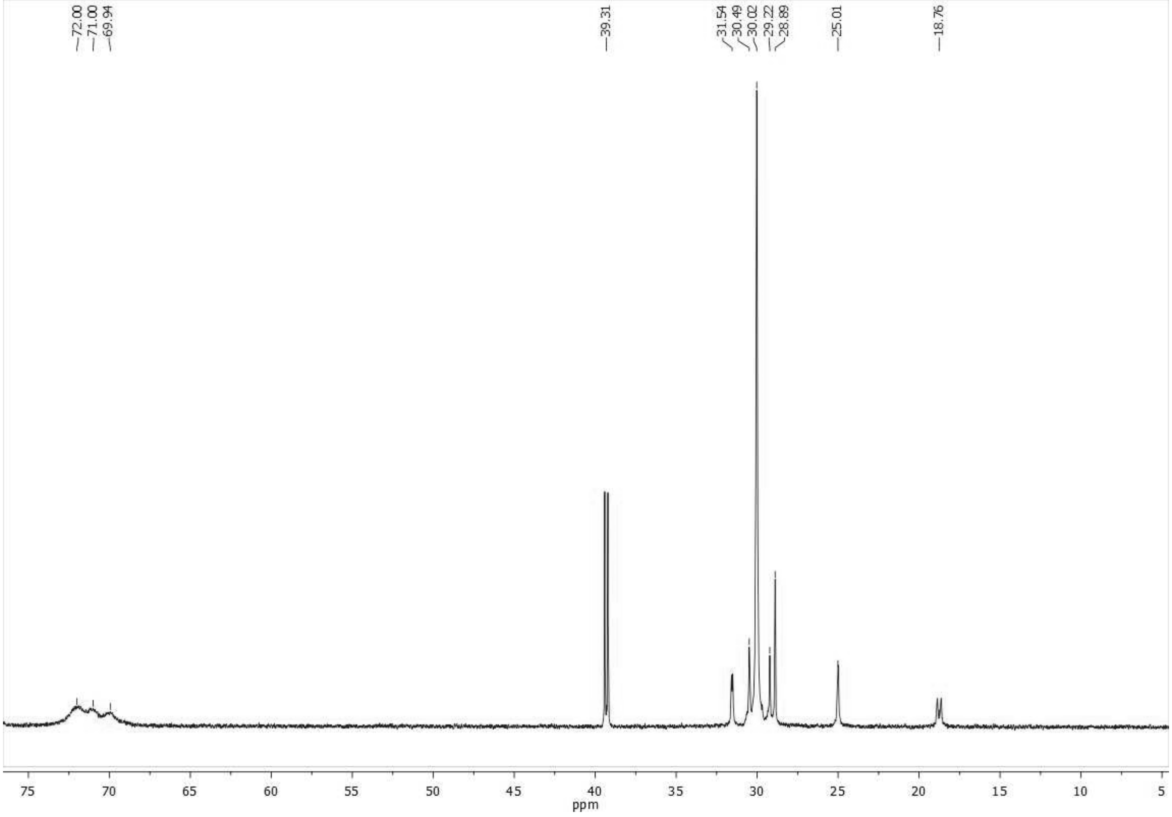

Figure S55. $^{13}C\{^1H\}$ NMR spectra of the tri-*tert*-butyl(6-ferrocenylhexyl)phosphonium tetrafluoroborate **8c**

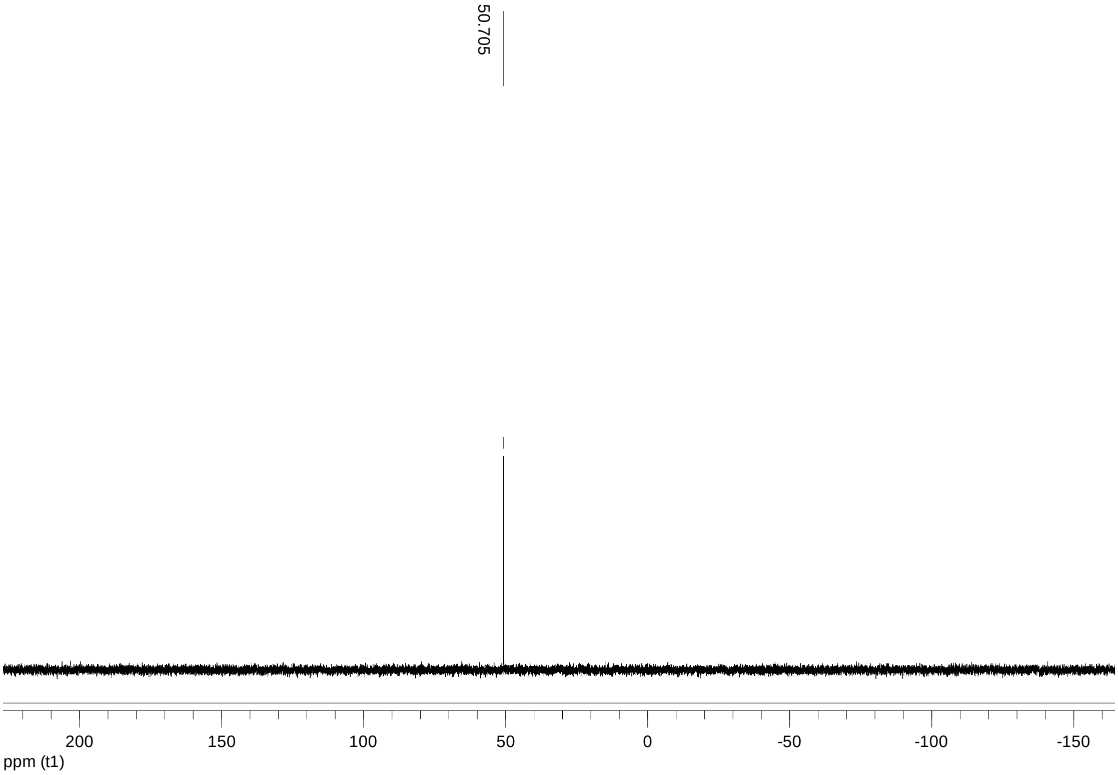

Figure S56. ^{31}P NMR spectra of the tri-*tert*-butyl(11-ferrocenyundecyl)phosphonium tetrafluoroborate **8d**

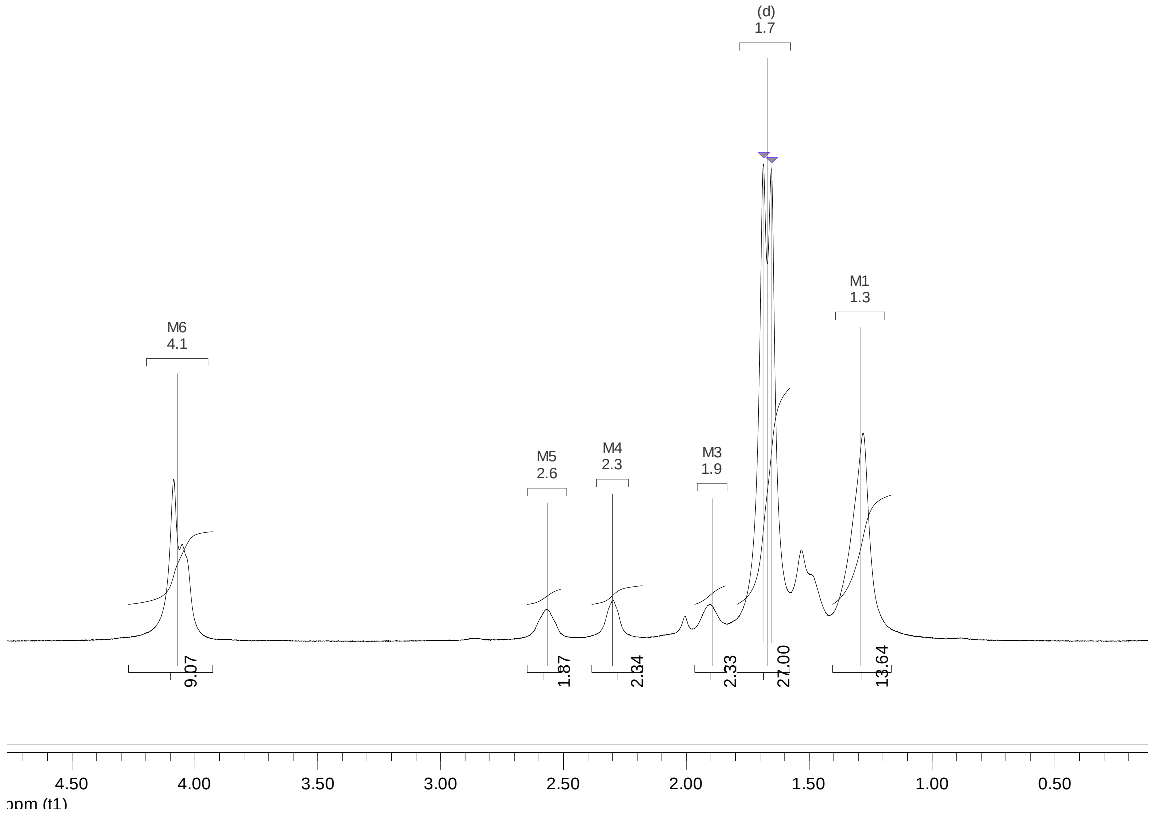

Figure S57.^{1}H NMR spectra of the tri-*tert*-butyl(11-ferrocenyundecyl)phosphonium tetrafluoroborate **8d**

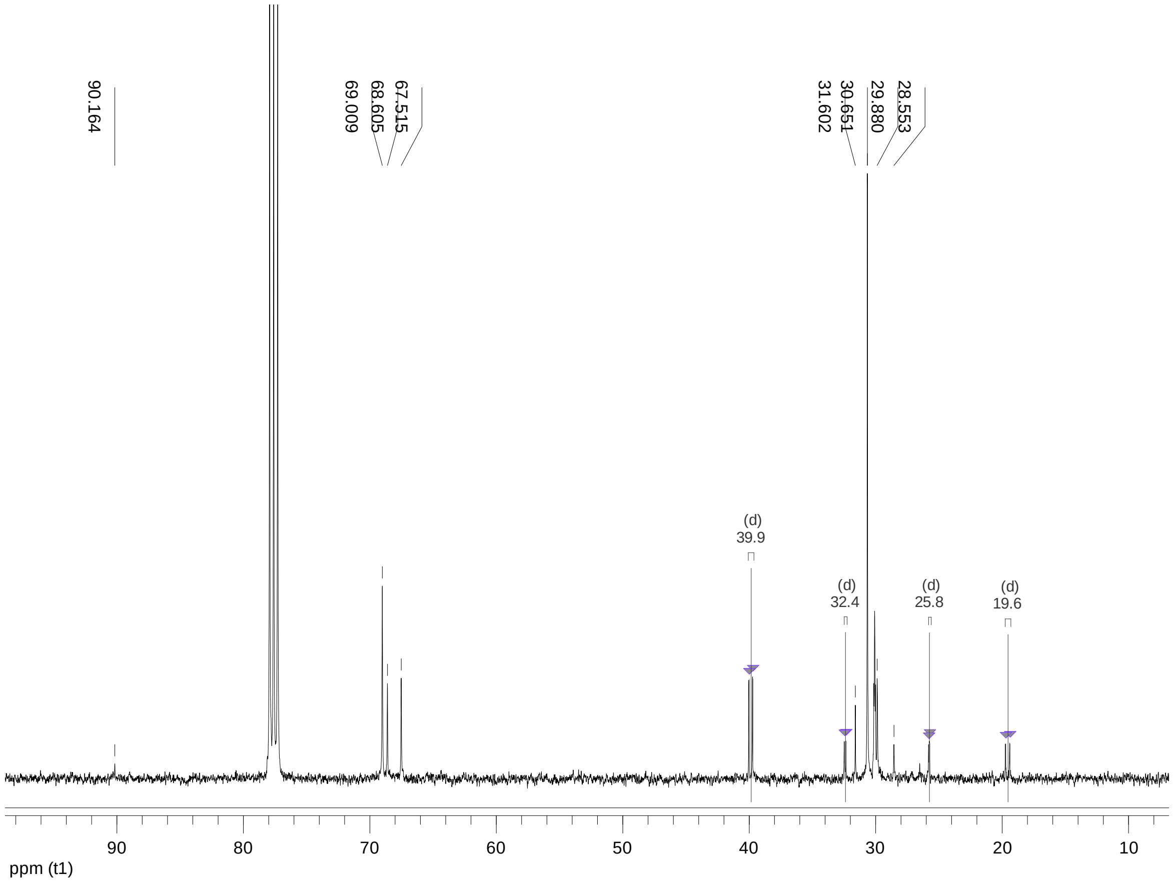

Figure S58. $^{13}C\{^{1}H\}$ NMR spectra of the tri-*tert*-butyl(11-ferrocenyundecyl)phosphonium tetrafluoroborate **8d**

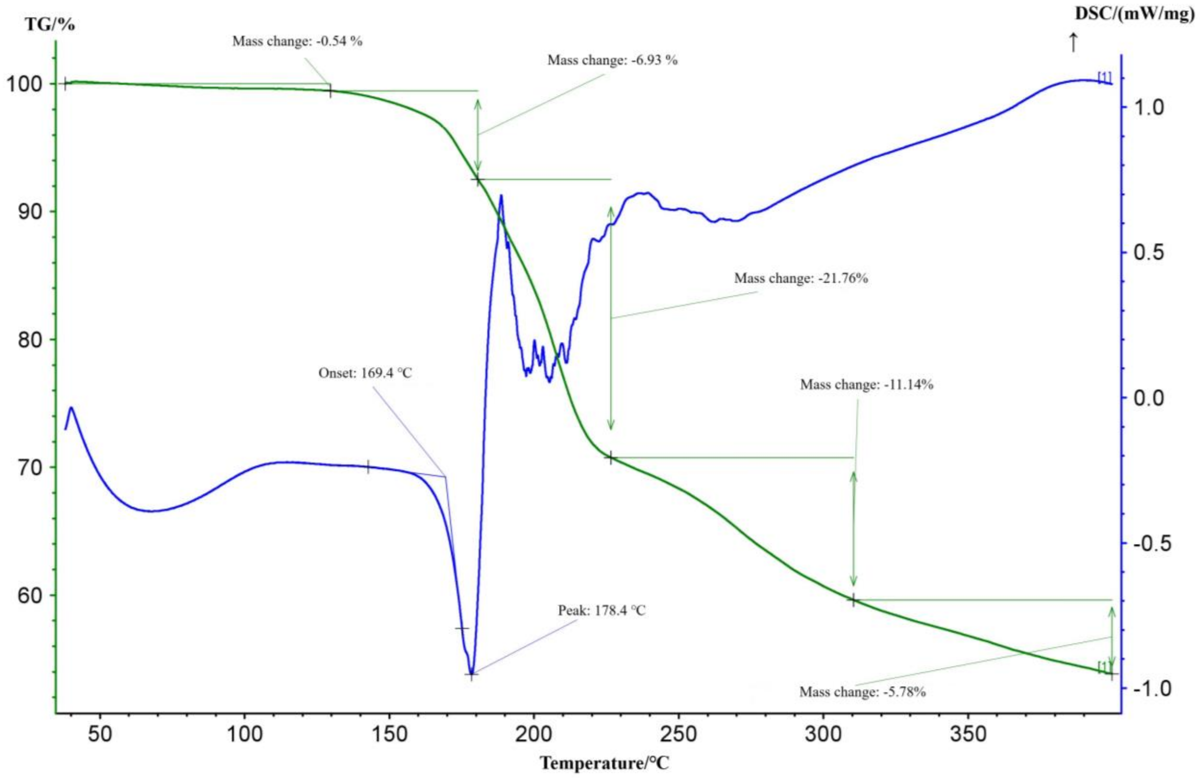

Figure S59. TG-DSC curve of the tri-*tert*-butyl(3-ferrocenyl-3-oxopropyl)phosphonium bromide **3a**

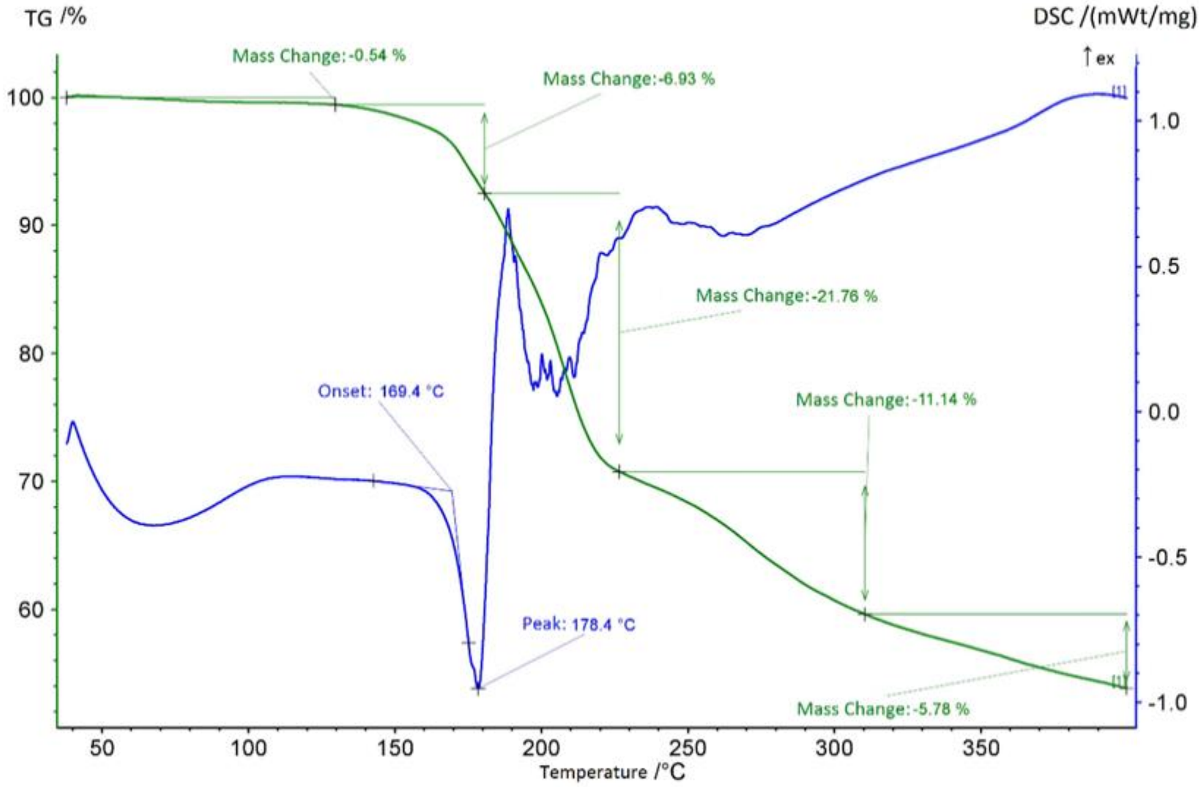

Figure S60. TG-DSC curve of the tri-*tert*-butyl(3-ferrocenyl-3-oxopropyl)phosphonium tetrafluoroborate **4a**

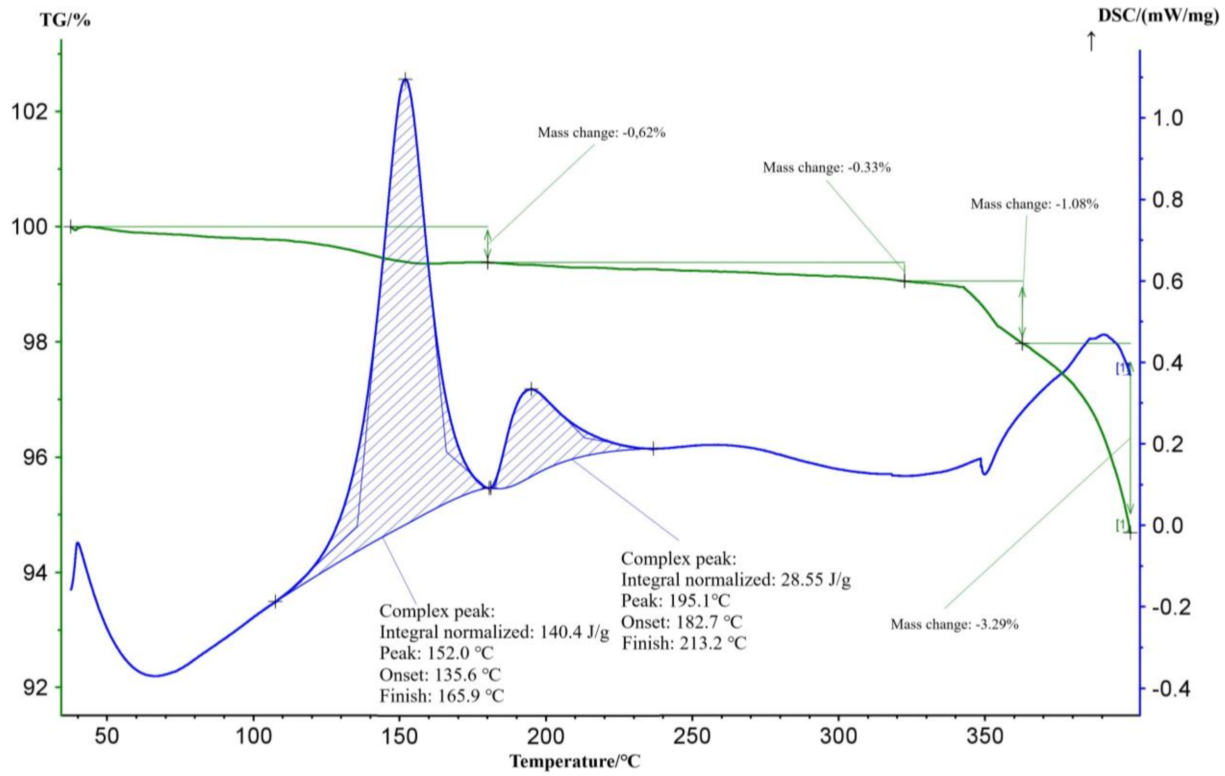

Figure S61.TG-DSC curve of the tri-*tert*-butyl(5-ferrocenyl-5-oxopentyl)phosphonium bromide **3b**

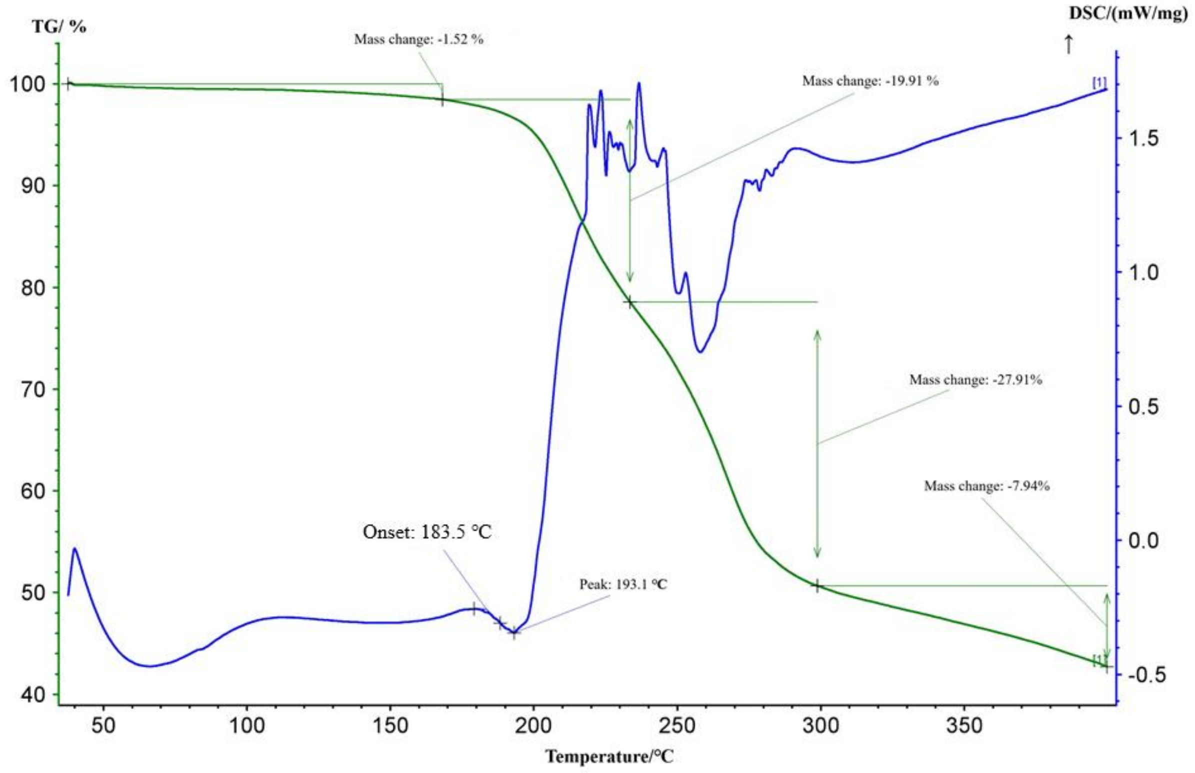

Figure S62. TG-DSC curve of the tri-*tert*-butyl(5-ferrocenyl-5-oxopentyl)phosphonium tetrafluoroborate **4b**

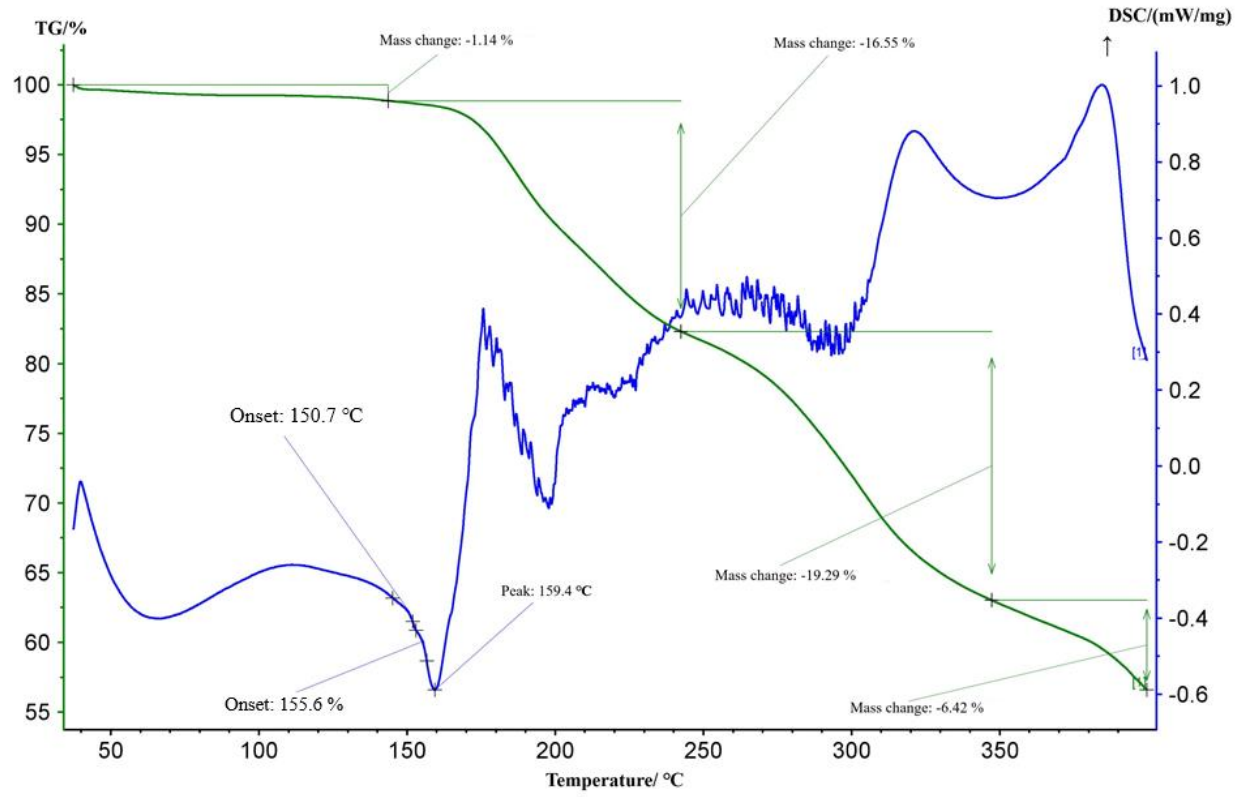

Figure S63. TG-DSC curve of the tri-*tert*-butyl(6-ferrocenyl-6-oxohexyl)phosphonium bromide **3c**

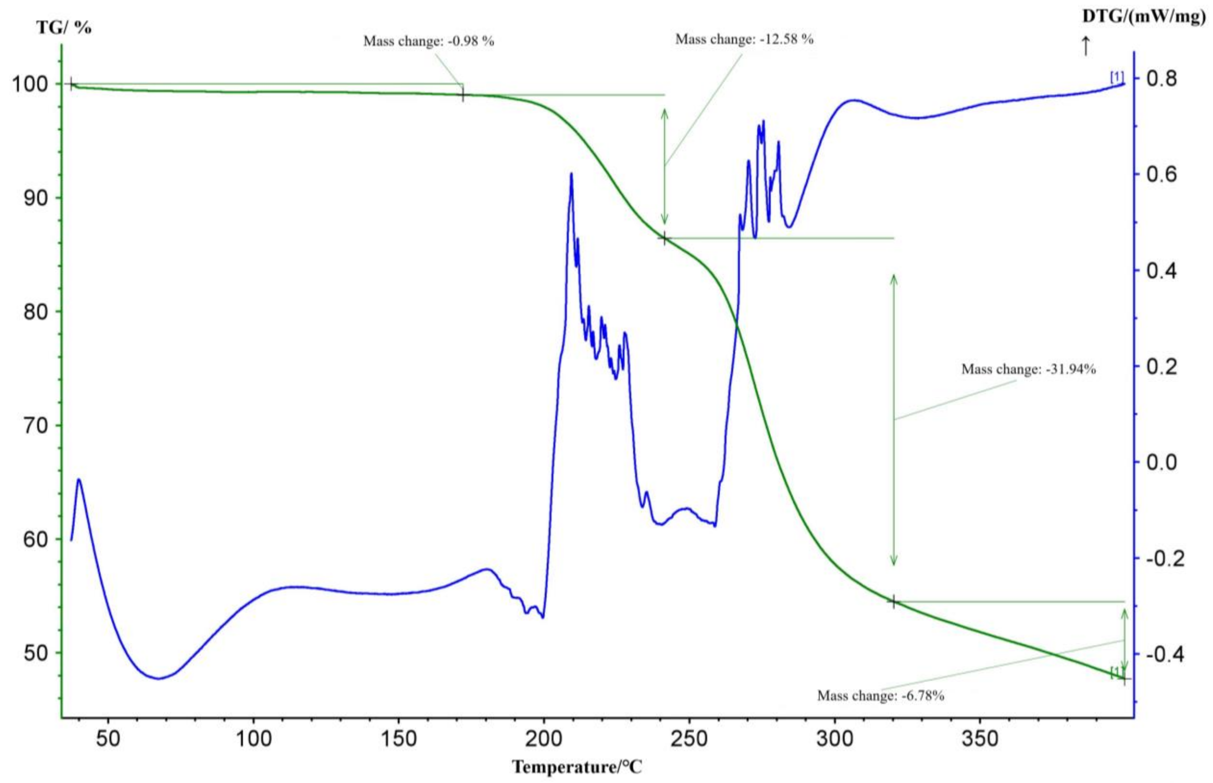

Figure S64. TG-DSC curve of the tri-*tert*-butyl(6-ferrocenyl-6-oxohexyl)phosphonium tetrafluoroborate **4c**

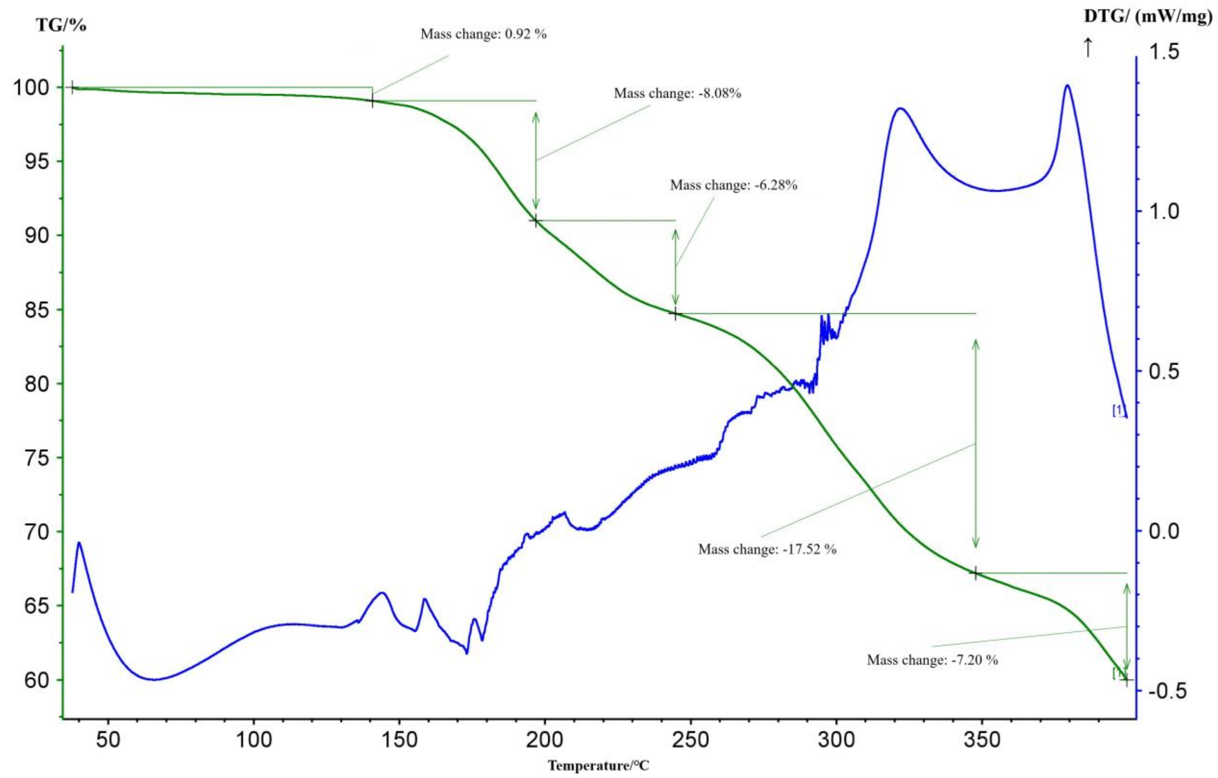

Figure S65. TG-DSC curve of the tri-*tert*-butyl(11-ferrocenyl-11-oxoundecyl)phosphonium bromide **3d**

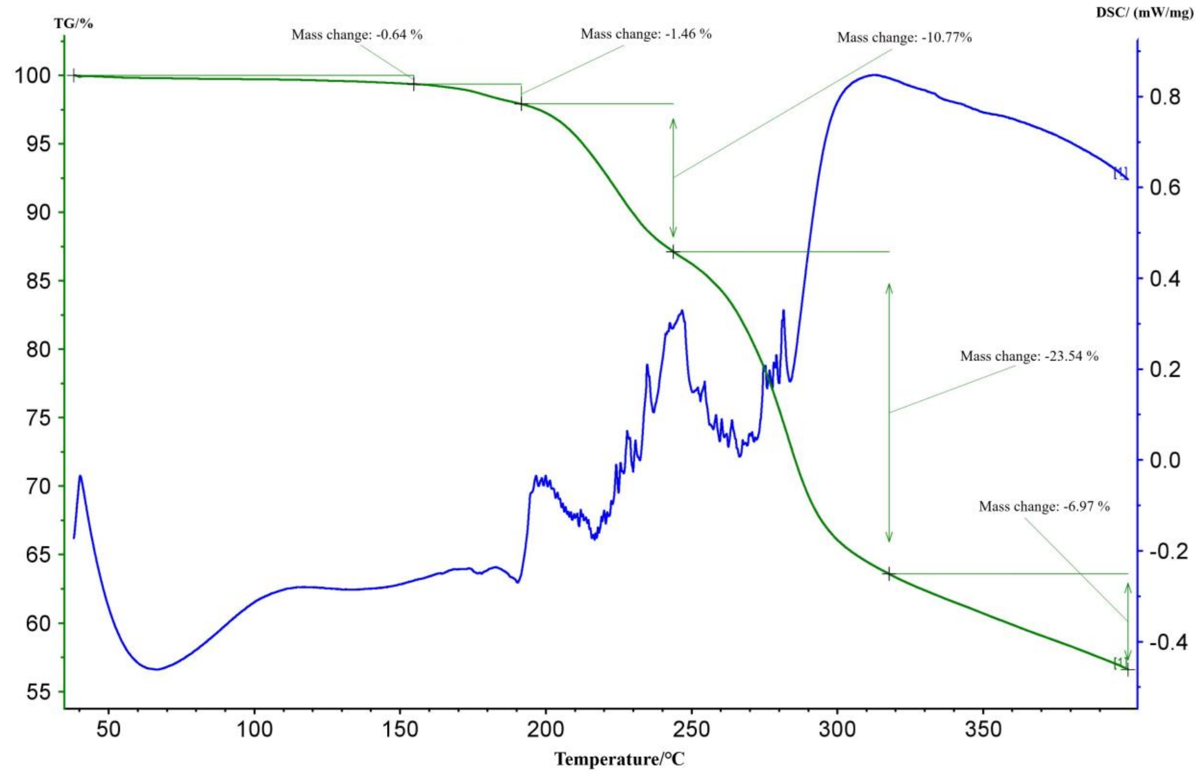

Figure S66. TG-DSC curve of the tri-*tert*-butyl(11-ferrocenyl-11-oxoundecyl)phosphonium tetrafluoroborate **4d**

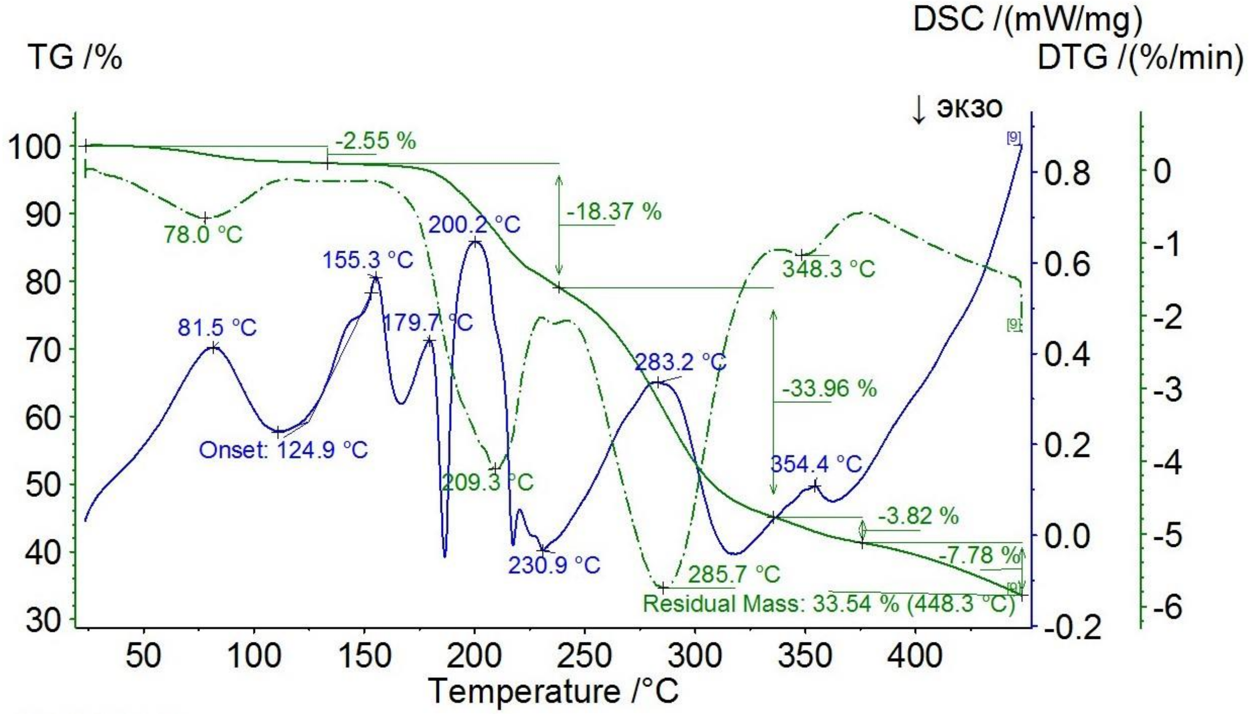

Figure S67. TG-DSC curve of the tri-*tert*-butyl(3-ferrocenylpropyl) phosphonium bromide **7a**

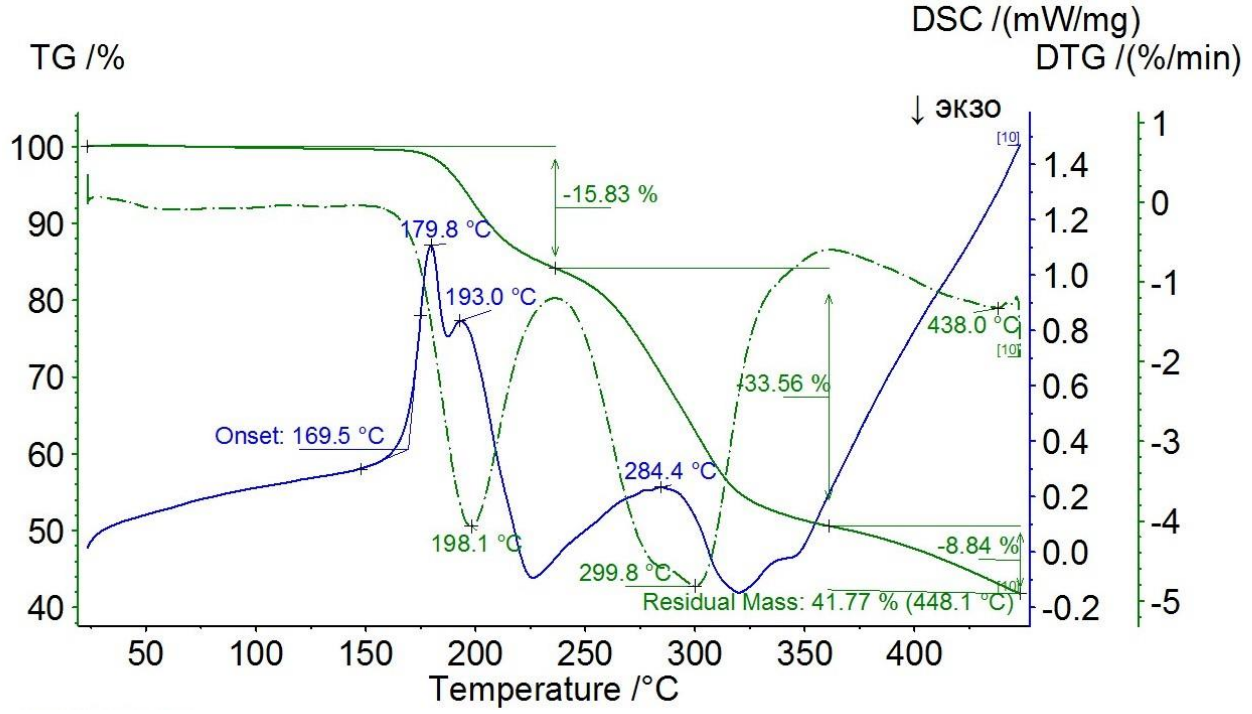

Figure S68. TG-DSC curve of the tri-*tert*-butyl(5-ferrocenylpentyl) phosphonium bromide **7b**

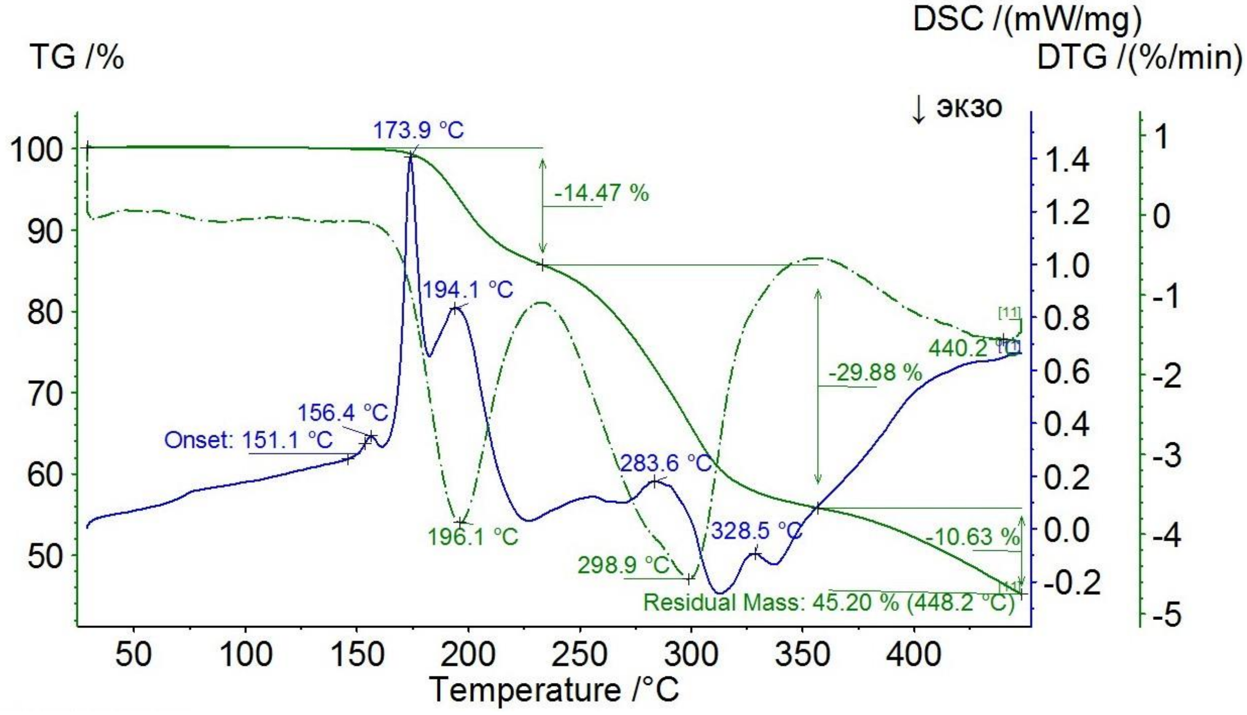

Figure S69. TG-DSC curve of the tri-*tert*-butyl(6-ferrocenylhexyl) phosphonium bromide **7c**

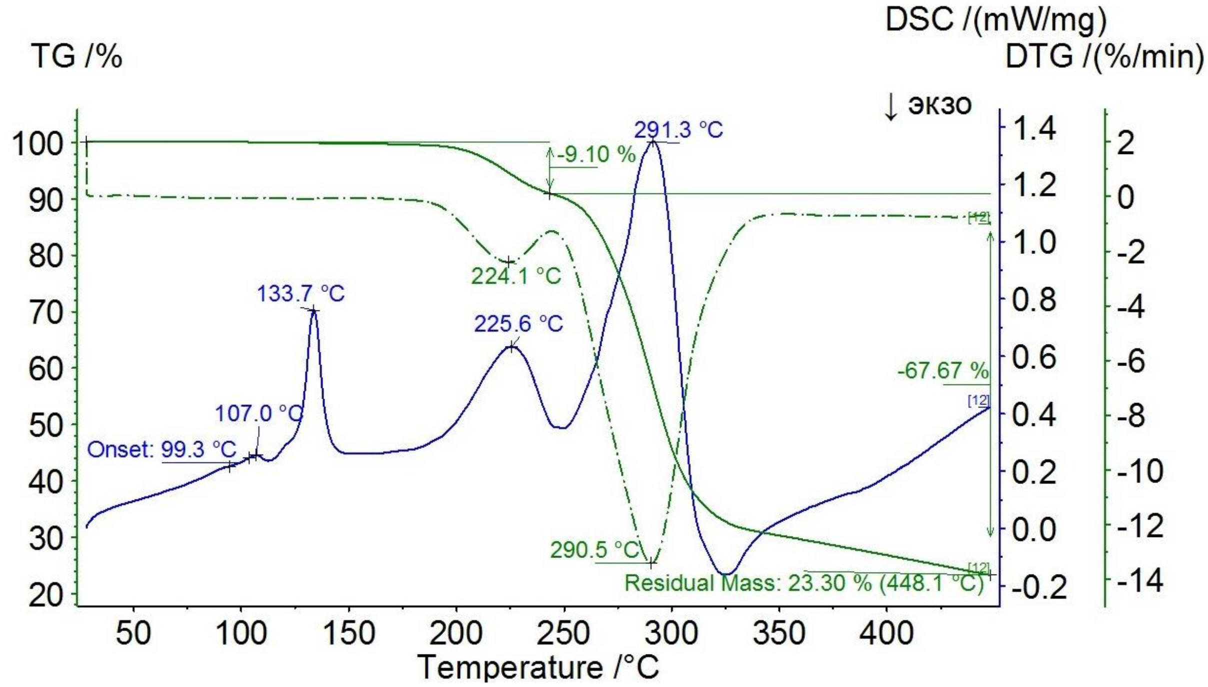

Figure S70. TG-DSC curve of the tri-*tert*-butyl(5-ferrocenylpentyl) phosphonium tetrafluoroborate **8b**

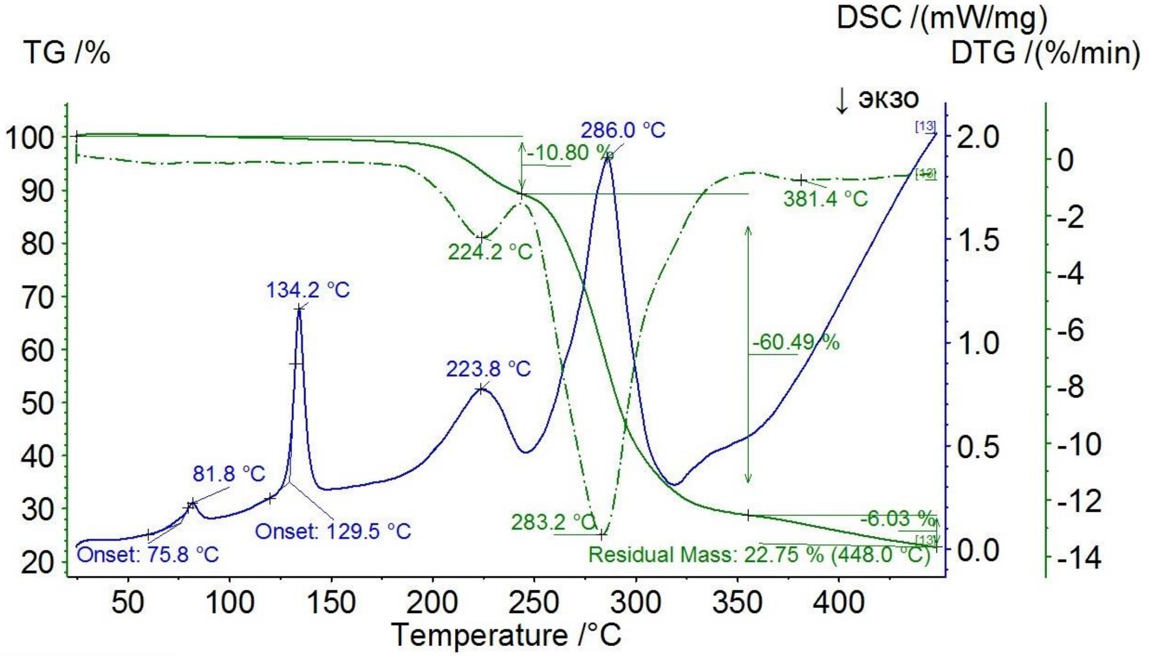

Figure S71. TG-DSC curve of the tri-*tert*-butyl(6-ferrocenylhexyl) phosphonium tetrafluoroborate **8c**

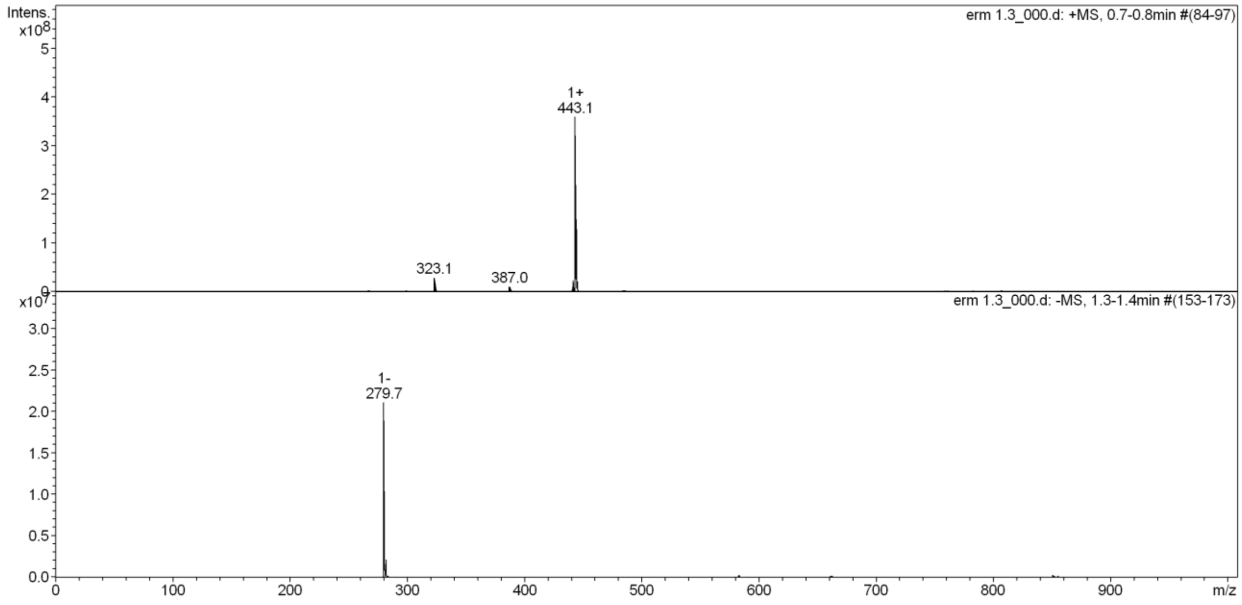

. **Figure S72.** ESI-MS spectra of the tri-*tert*-butyl(3-ferrocenyl-3-oxopropyl)phosphonium bis(trifluoromethanesulfonyl)imide **5a**

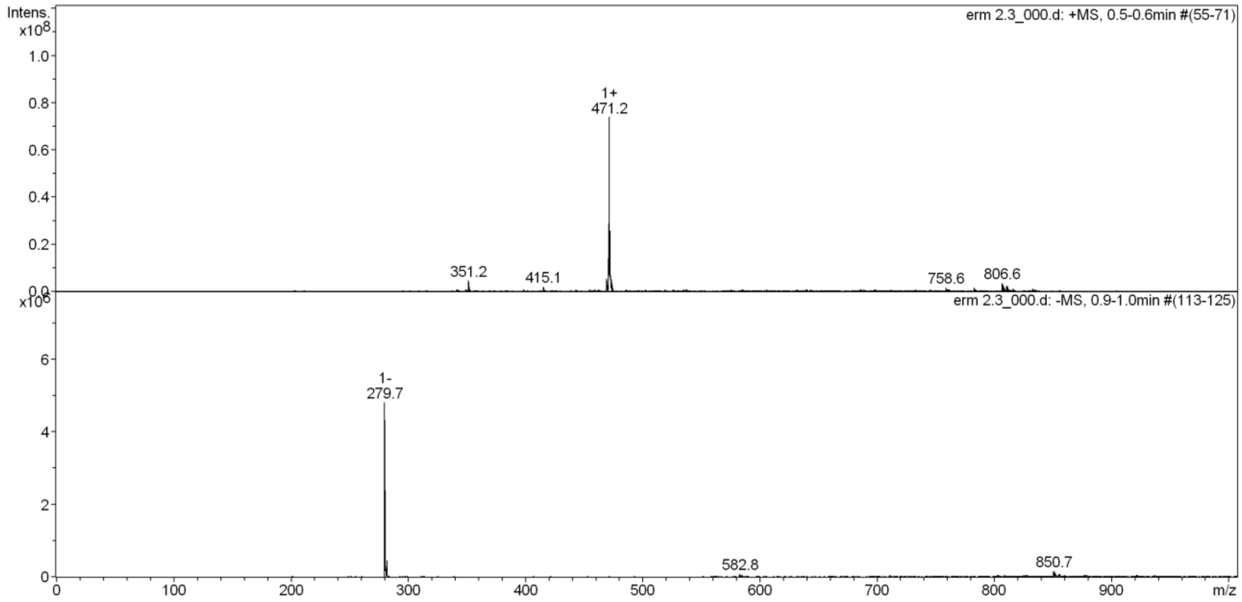

Figure S73. ESI-MS spectra of the tri-*tert*-butyl(5-ferrocenyl-5-oxopentyl) phosphonium bis(trifluoromethanesulfonyl)imide **5b**

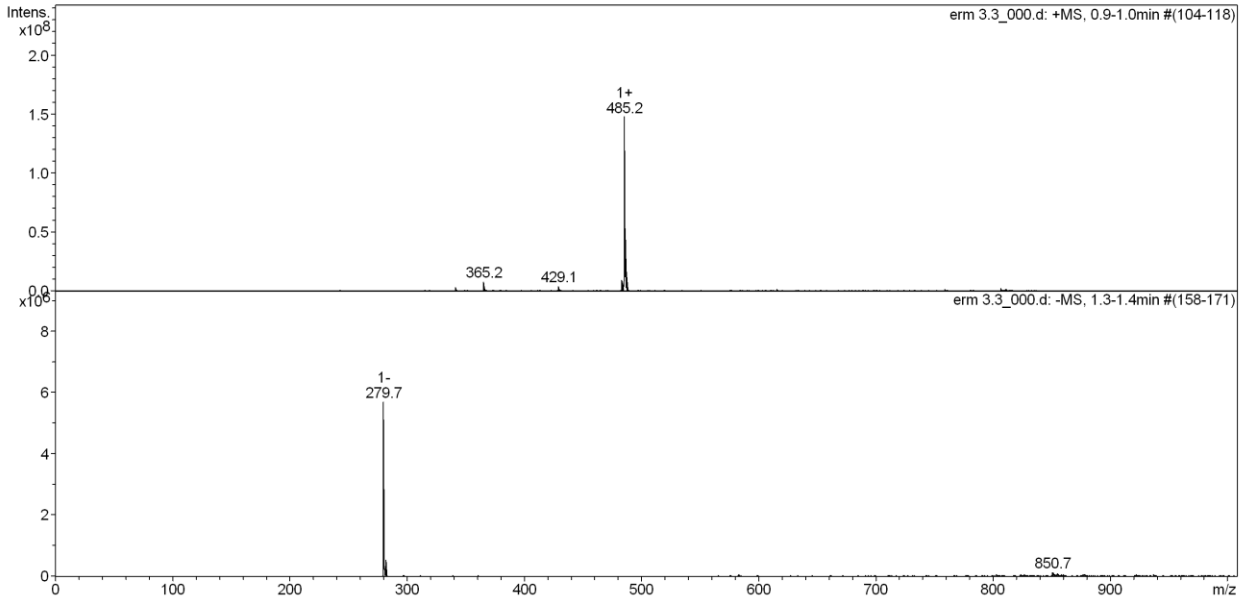

Figure S74. ESI-MS spectra of the tri-*tert*-butyl(6-ferrocenyl-6-oxohexyl) phosphonium bis(trifluoromethanesulfonyl)imide **5c**

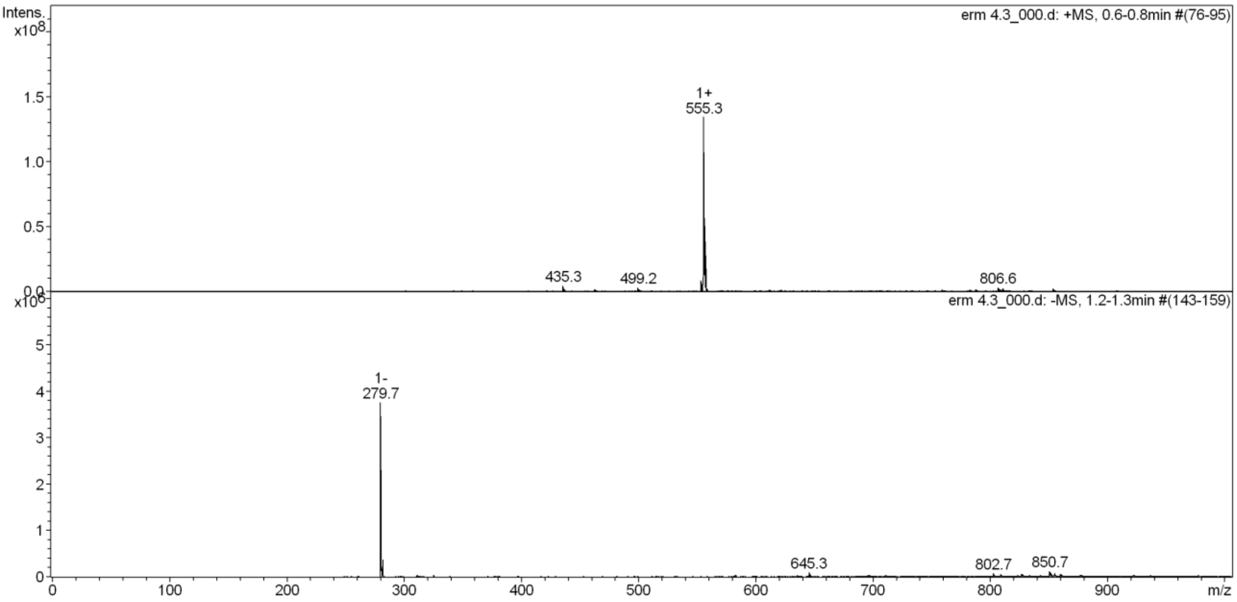

Figure S75. ESI-MS spectra of the tri-*tert*-butyl(11-ferrocenyl-11-oxoundecyl) phosphonium bis(trifluoromethanesulfonyl)imide **5d**

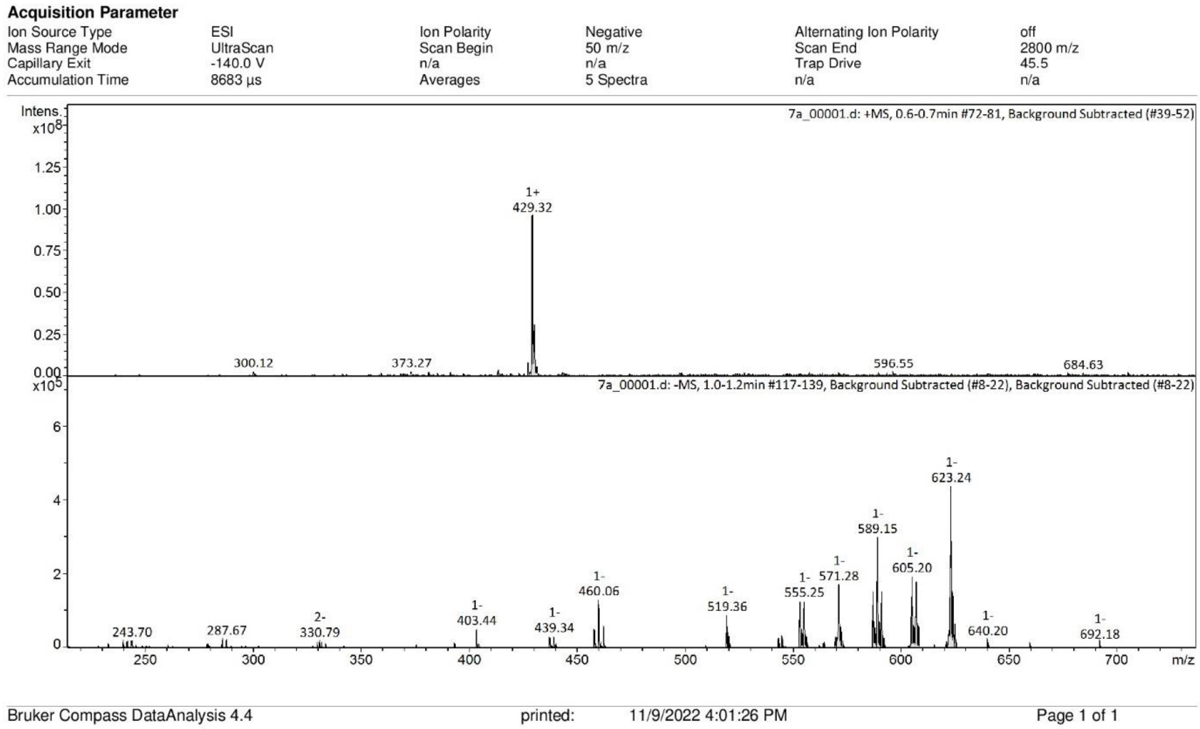

Figure S76. ESI-MS spectral of the tri-*tert*-butyl(3-ferrocenylpropyl) phosphonium bromide **7a**

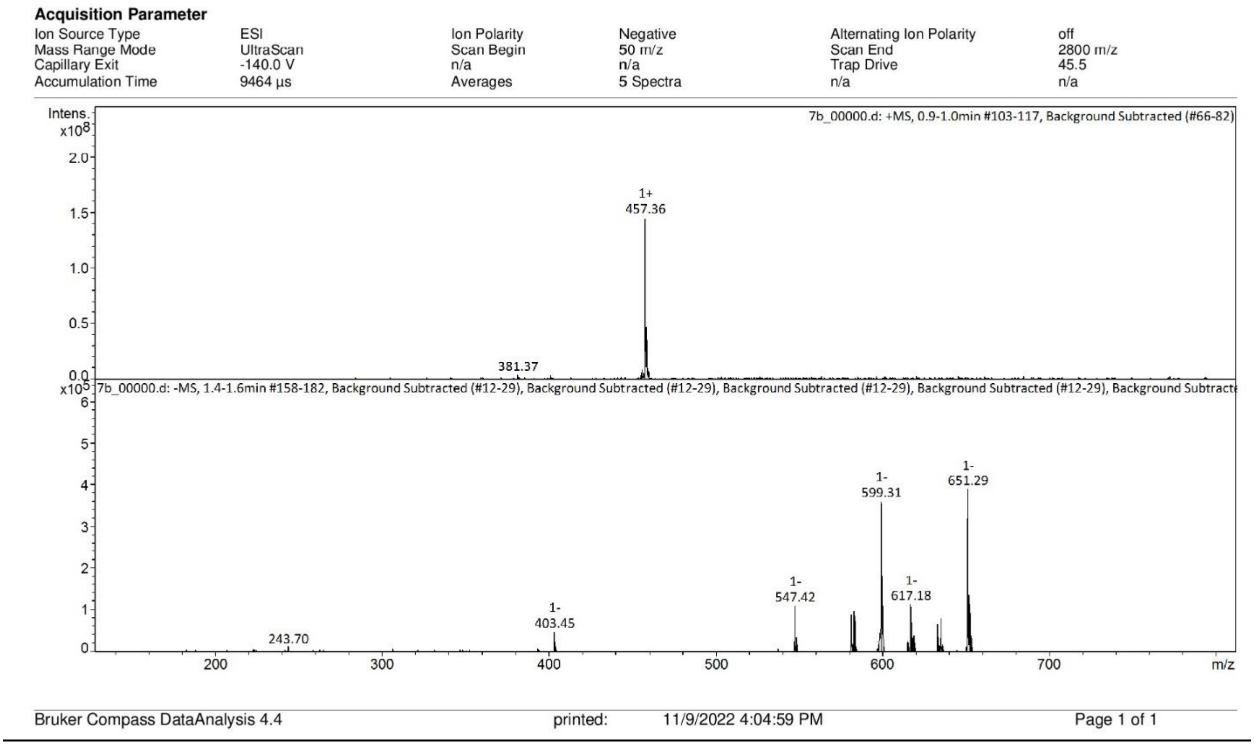

Figure S77. ESI-MS spectral of the tri-*tert*-butyl(5-ferrocenylpentyl) phosphonium bromide **7b**

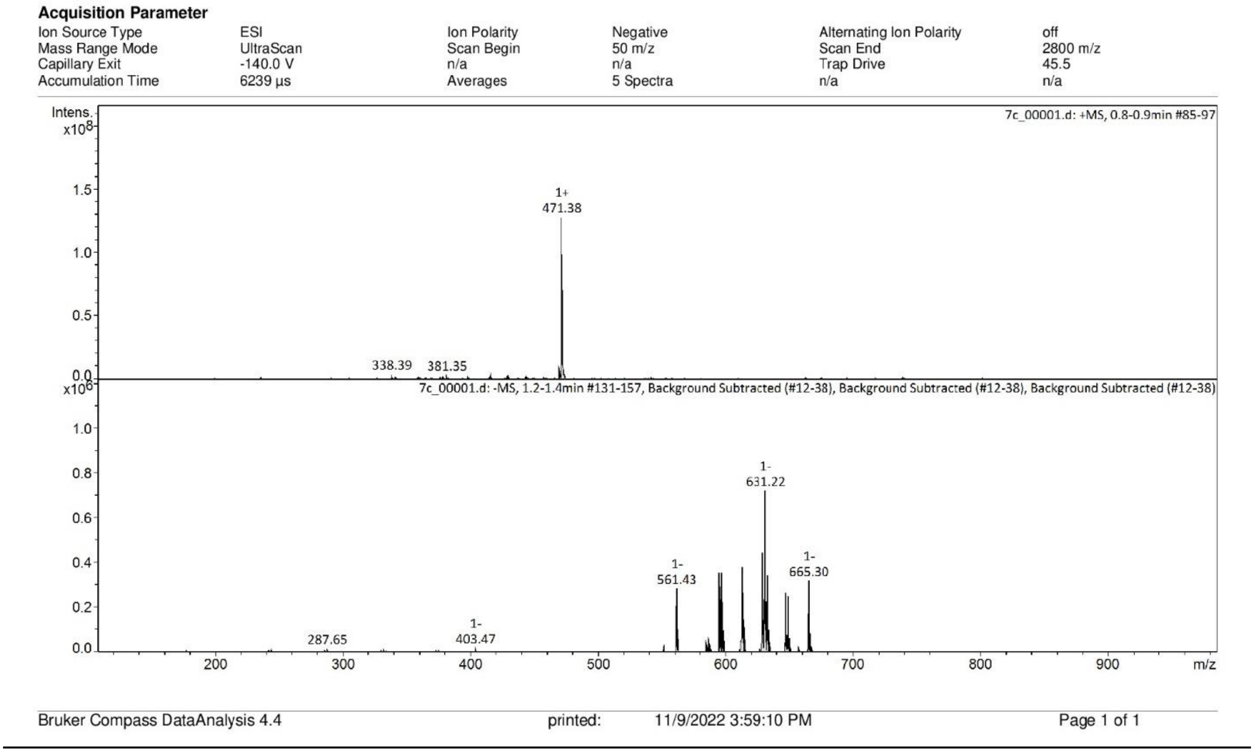

Figure S78. ESI-MS spectral of the tri-*tert*-butyl(6-ferrocenylhexyl) phosphonium bromide **7c**

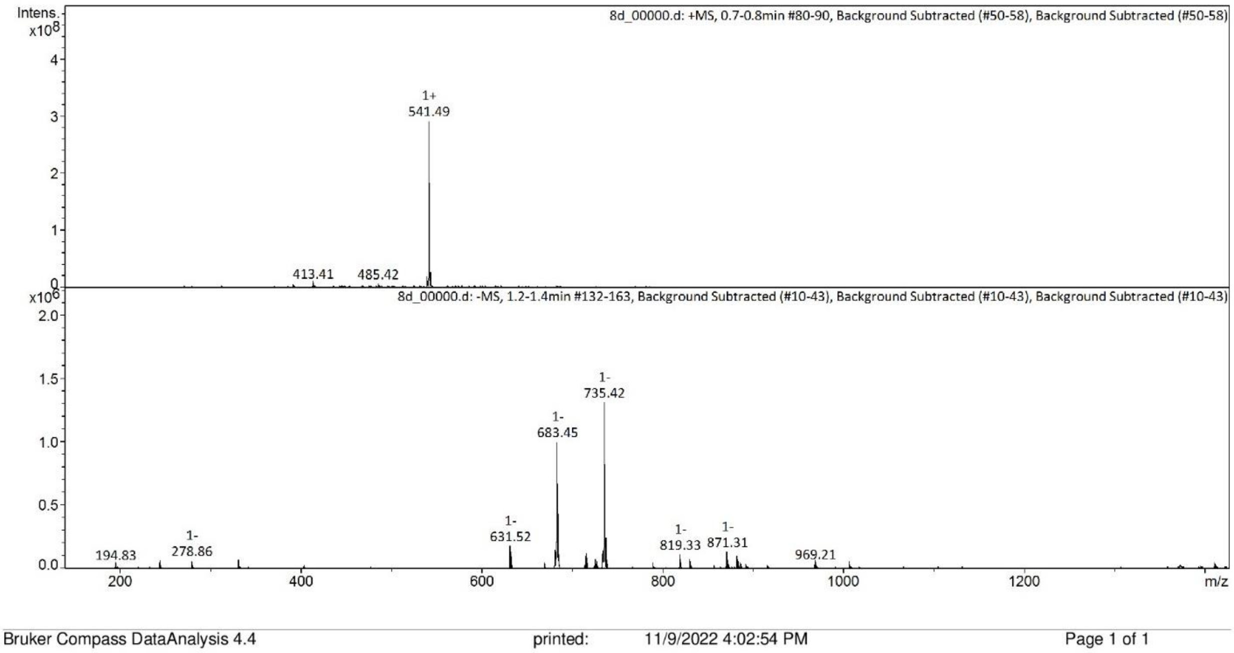

Figure S79. ESI-MS spectral of the tri-*tert*-butyl(11-ferrocenylundecyl) phosphonium bromide **7d**

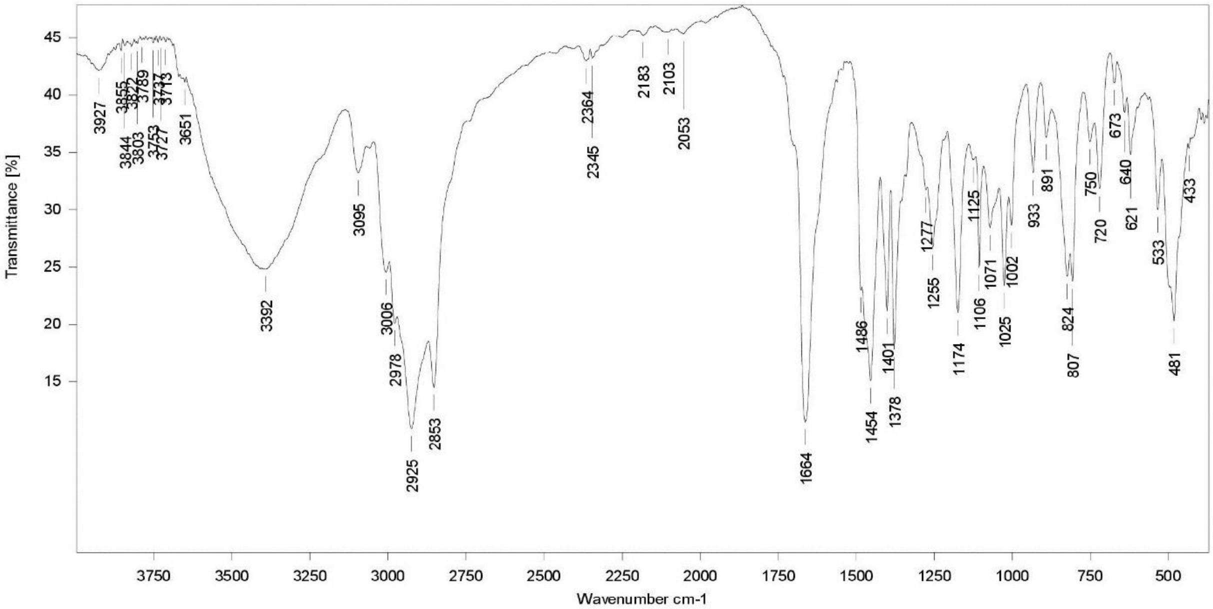

Figure S80. IR spectral of the tri-*tert*-butyl(11-ferrocenyl-11-oxoundecyl) phosphonium bromide **3d**

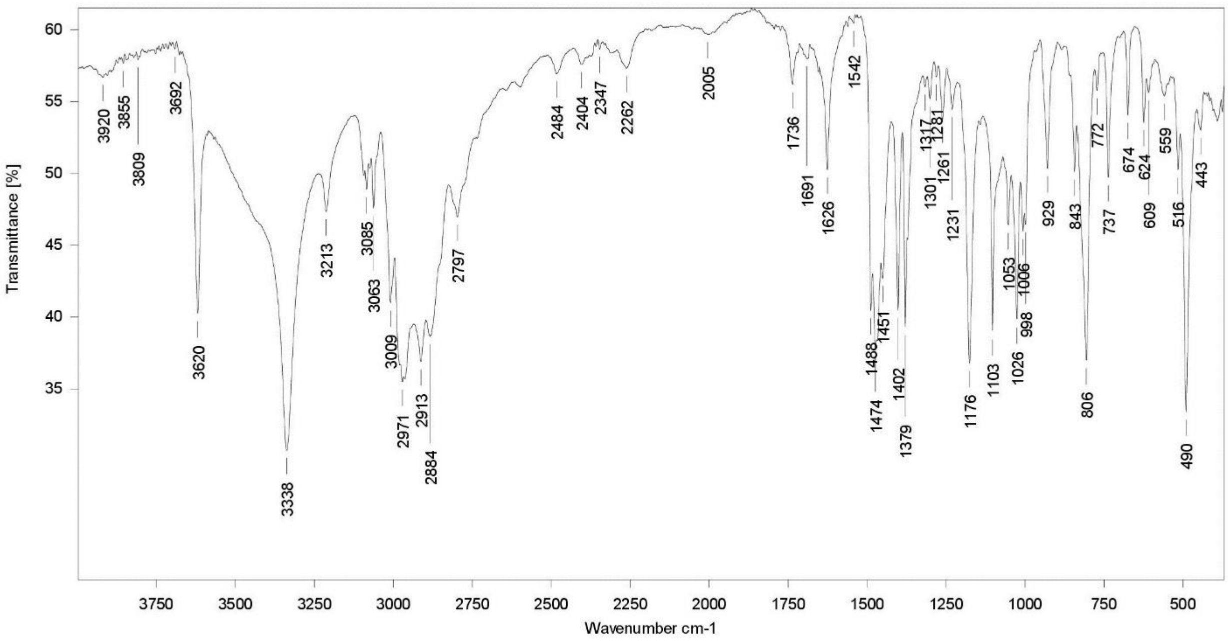

Figure S81. IR spectral of the tri-*tert*-butyl(3-ferrocenylpropyl) phosphonium bromide **7a**

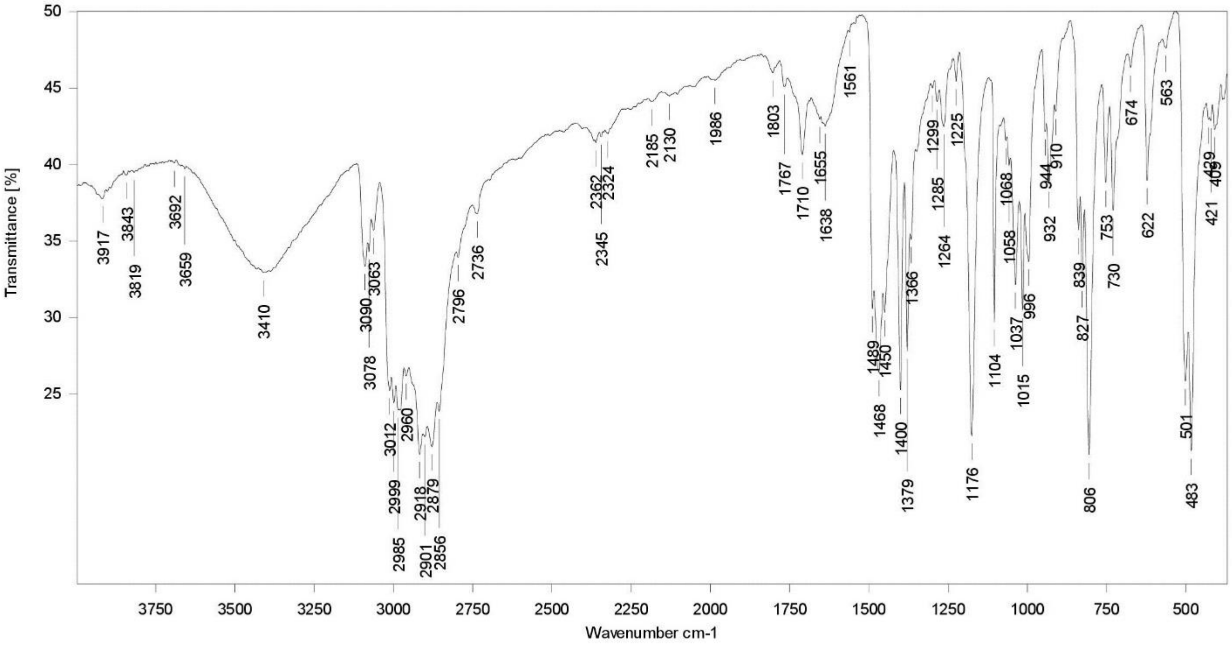

Figure S82. IR spectral of the tri-*tert*-butyl(5-ferrocenylpentyl) phosphonium bromide **7b**

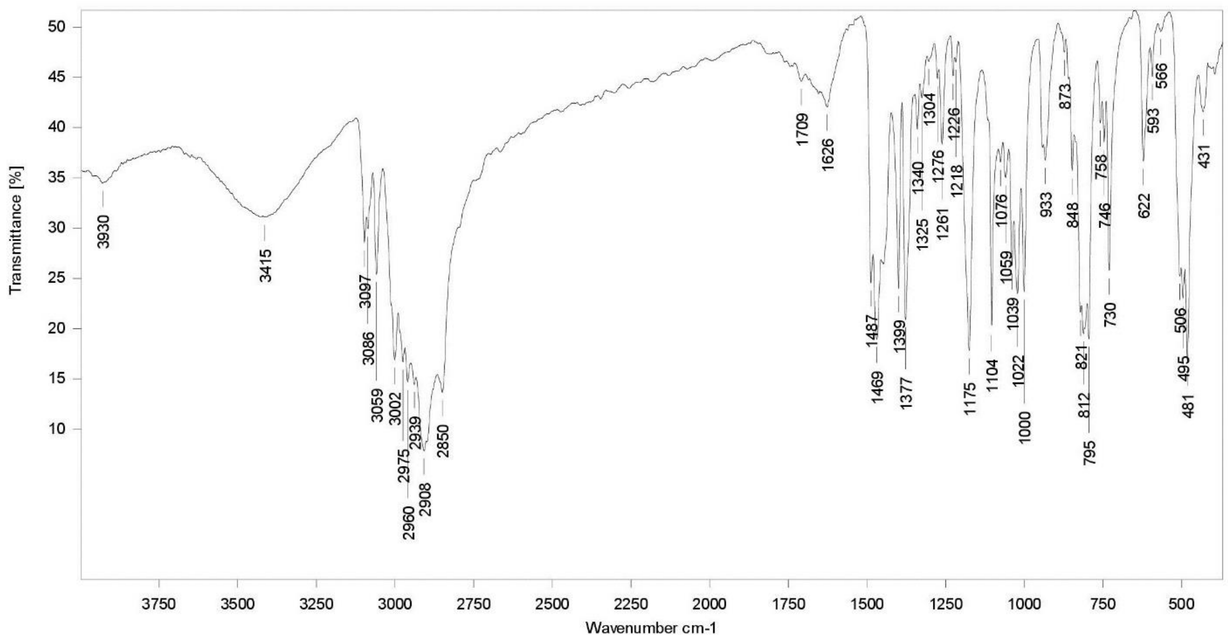

Figure 83. IR spectral of the tri-*tert*-butyl(6-ferrocenylhexyl) phosphonium bromide **7c**

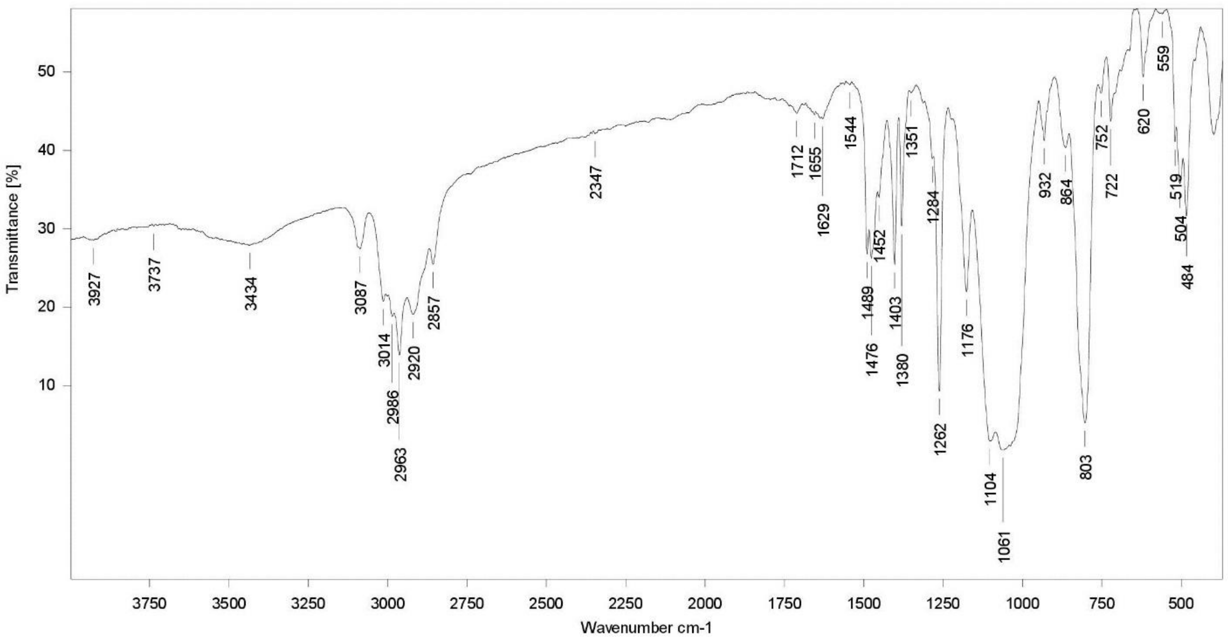

Figure S84. IR spectral of the tri-*tert*-butyl(5-ferrocenylpentyl) phosphonium tetrafluoroborate **8b**

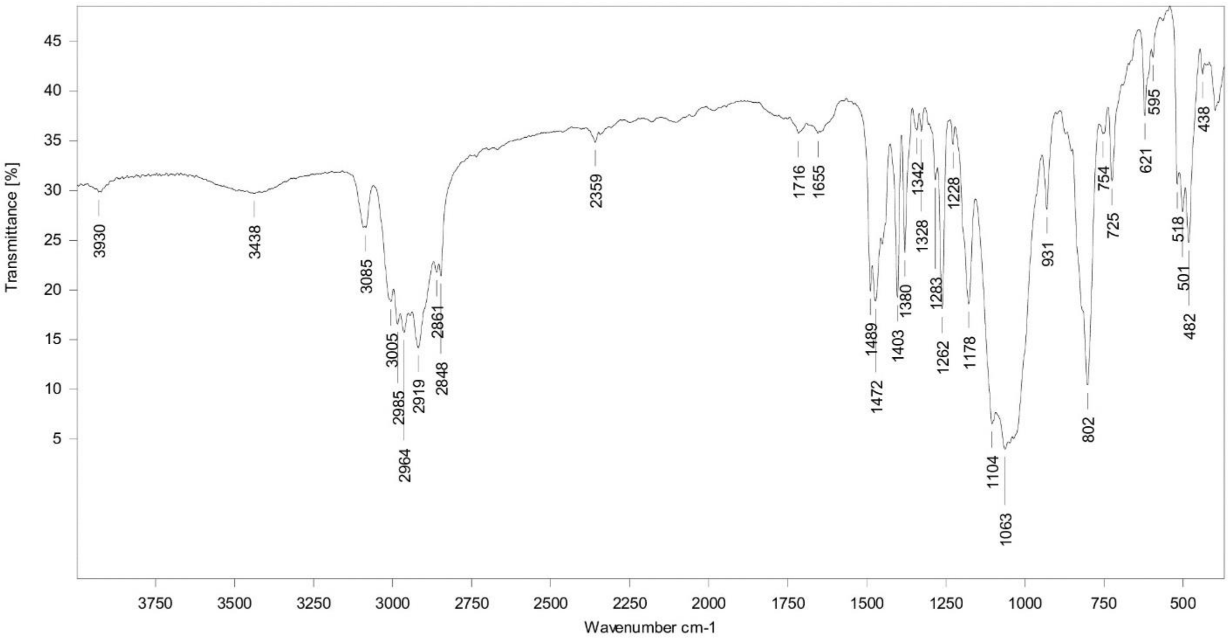

Figure S85. IR spectral of the tri-*tert*-butyl(6-ferrocenylhexyl) phosphonium tetrafluoroborate **8c**

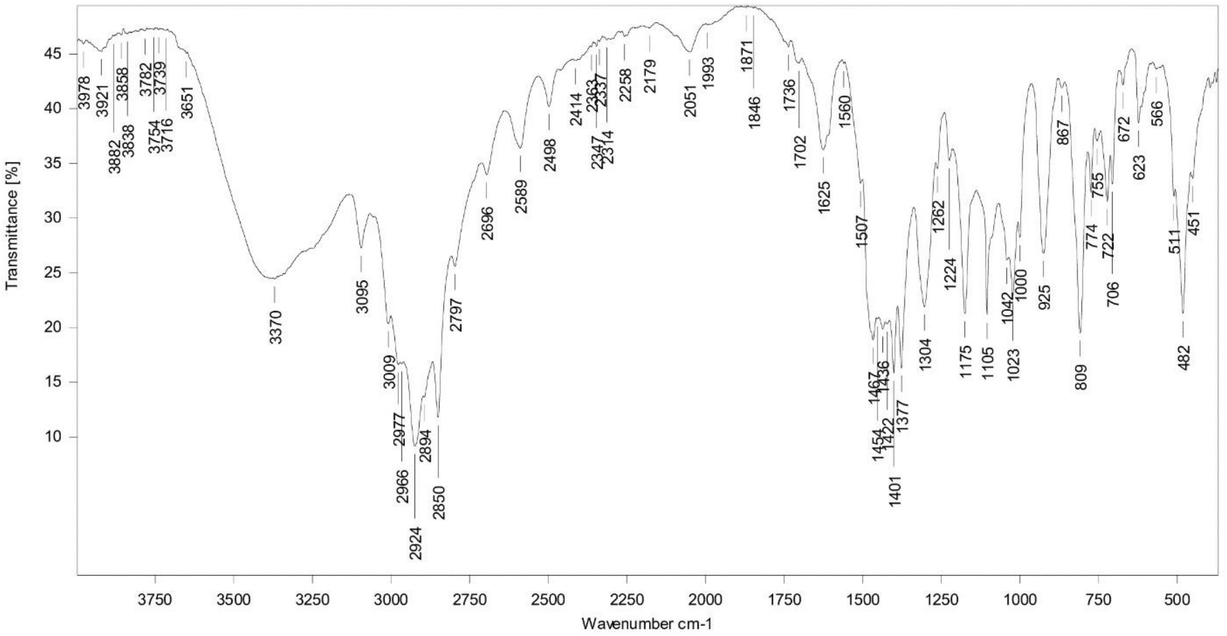

. **Figure S86.** IR spectral of the tri-*tert*-butyl(11-ferrocenyundecyl) phosphonium tetrafluoroborate **8d**
